# Antimicrobial Terpenoids from South China Sea Soft Coral *Lemnalia* sp.

**DOI:** 10.3390/md19060294

**Published:** 2021-05-22

**Authors:** Xia Yan, Han Ouyang, Wei Wang, Jing Liu, Te Li, Bin Wu, Xiaojun Yan, Shan He

**Affiliations:** 1 Li Dak Sum Yip Yio Chin Kenneth Li Marine Biopharmaceutical Research Center, Department of Marine Pharmacy, College of Food and Pharmaceutical Sciences, Ningbo University, Ningbo 315800, China; yanxia@nbu.edu.cn (X.Y.); liujingsally@163.com (J.L.); telinbu@163.com (T.L.); yanxiaojun@nbu.edu.cn (X.Y.); 2 Institute of Drug Discovery Technology, Ningbo University, Ningbo 315211, China; 3 Key Laboratory of Marine Drugs, Chinese Ministry of Education, School of Medicine and Pharmacy, Ocean University of China, Qingdao 266003, China; wwwakin@ouc.edu.cn; 4 Ocean College, Zhejiang University, Hangzhou 310058, China; wubin@zju.edu.cn

**Keywords:** soft coral, *Lemnalia* sp., terpenoids, antimicrobial activity, cytotoxicity

## Abstract

Chemical investigation of the South China Sea soft coral *Lemnalia* sp. afforded 13 structurally diverse terpenoids, including three new neolemnane sesquiterpene lineolemnenes E–G (**1**–**3**); a new aristolane-type sesquiterpenoid, 2-acetoxy-aristolane (**4**); four new decalin-type bicyclic diterpenes, named biofloranates A−D (**5**−**8**); a new serrulatane, named euplexaurene D (**9**); and a new aromadendrane-type diterpenoid cneorubin K (**10**), together with three known related compounds (**11**−**13**). The structures of the new compounds were elucidated by NMR spectroscopy, the Mosher’s method, and ECD analysis. Compounds **1**–**13** were tested in a wide panel of biological assays. Lineolemnene J (**3**) showed weak cytotoxicity against the CCRF-CEM cancer cell line. The isolated new diterpenes, except euplexaurene D (**9**), demonstrated moderate antimicrobial activity against *Bacillus subtilis* and *Staphylococcus aureus* with a MIC of 4−64 μg/mL. Compound **2** exhibited a mild inhibitory effect against influenza A H1N1 virus (IC_50_ = 5.9 µM).

## 1. Introduction

In the competition for food and living space for survival, marine organisms accumulate and secrete secondary metabolites that are deterrent, offensive, or even toxic to other organisms—that is, chemical defense substances [1]. Marine invertebrates, such as soft corals, grow slowly and lack physical defense capabilities, but they can survive in competitive marine environments. What they often depend on are chemical defense strategies, which represent a rich source of unexplored chemical structures and biological diversity with great potential for drug discovery [2].

The South China Sea is located in the tropics and subtropics, with a vast sea area and rich marine biological resources. It is a sea area with numerous coral distributions. Since the 1980s, researchers have studied the chemical composition of many kinds of corals collected from the South China Sea [3,4,5,6,7,8]. Many compounds with novel structures have been isolated, including steroids, terpenoids, alkaloids, etc. Among them, the marine soft coral genus *Lemnalia* has afforded structurally diverse novel terpenoids, including sesquiterpenes and diterpene glycosides, with promising biological activity [7].

**Figure 1:**
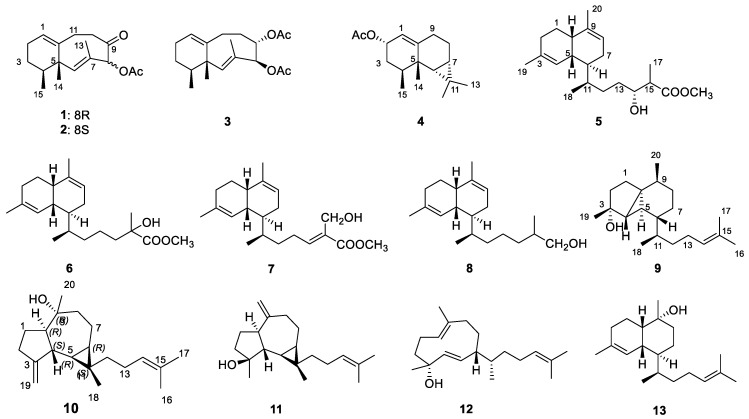
Structures of compounds **1**–**13**.

In the course of our efforts to search for novel and bioactive natural marine products from soft corals and find more diverse and complex terpenoids, we obtained *Lemnalia* sp. (No. XSSC201907), a soft coral collected from the coast of Xisha Islands in the South China Sea. The acetone extracts were chemically investigated, resulting in the discovery of 13 structurally diverse terpenoids, including four new sesquiterpenes (**1**–**4**), six new diterpenoids (**5**–**10**), and three known related compounds (**11**–**13**) (Figure 1). In vitro cytotoxic, antimicrobial, and antiviral activity of the isolated compounds were investigated. Herein, their isolation, structure, and biological activity are described.

## 2. Results and Discussion

**Figure 2:**
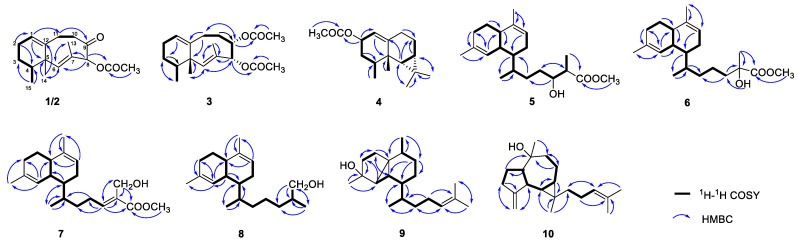
Key ^1^H-^1^H COSY and HMBC correlations of **1**–**10**.

Compound **1** was isolated as a colorless oil; its HR-ESI-MS exhibited an ion peak at *m/z* 299.1616 [M + Na]^+^, corresponding to the molecular formula of C_17_H_24_O_3_Na (six degrees of unsaturation). The ^13^C NMR spectrum of **1** showed 17 signals (Table 1), in combined ^1^H NMR and HSQC experiments, indicating the presence of a ketone group (*δ*_C_ 201.2), an acetyl group (*δ*_C_ 170.1 and *δ*_C_/*δ*_H_ 20.8/2.18), two double bonds (*δ*_C_ 140.4 and *δ*_C_/*δ*_H_ 123.4/5.43; *δ*_C_ 127.8 and *δ*_C_/*δ*_H_ 138.6/5.82), an oxygenated methine (*δ*_C_/*δ*_H_ 85.5/5.66), a nonprotonated carbon (*δ*_C_ 46.2), two methylenes, four methines, and four methyls. The above signals revealed that **1** was a bicyclic structure. The neolemnane skeleton was established by extensive 2D NMR analysis [9]. The ^1^H-^1^H COSY spectrum revealed the connectivity of H-1/H_2_-2/H_2_-3/H-4/H_3_-15 and H_2_-10/H_2_-11 (Figure 2). The vinyl methyl (*δ*_C_/*δ*_H_ 13.0/1.51) attached to C-7 (*δ*_C_ 127.8) was confirmed by HMBC correlations from H_3_-13 to C-6, C-7, and C-8. Together with further HMBC correlations from H-8 to C-6/C-7/C-13 and the carbonyl of the ester group, the planar structure of **1** was established. Moreover, established by HR-ESI-MS, compound **2** was found to possess the same molecular formula as **1**. A comparison of the 1D and 2D NMR spectral data of **2** with those of **1** disclosed that they had the same planar structure.

**Figure 3:**
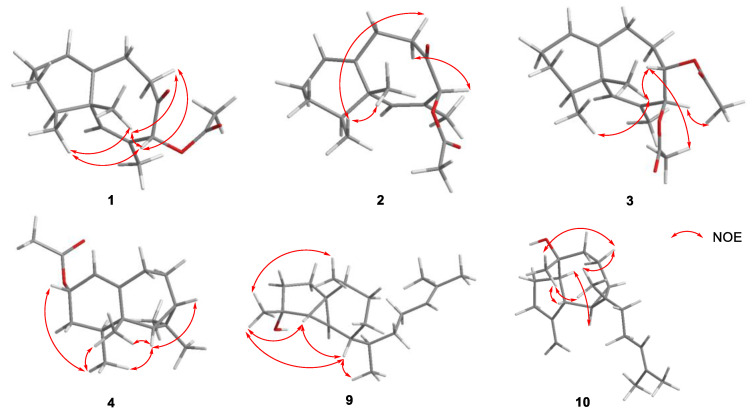
Key NOESY correlations of **1**–**4** and **9**–**10**.

**Figure 4:**
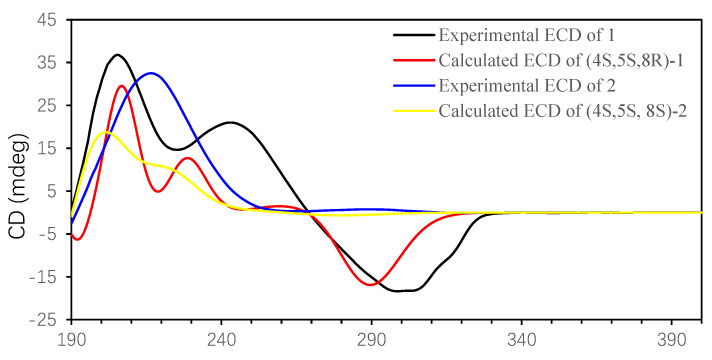
Experimental and calculated ECD spectra of compounds **1** and **2**.

The geometry of the 6,7-double bonds of compounds **1** and **2** was elucidated from NOE correlations (Figure 3). The absence of an NOE correlation for H_3_-13/H-6 suggests an *E* geometry of the 6,7-double bond, which was different from the known lineolemnene A and B [10]. For compound **1**, the NOESY correlations from H_3_-14 to H_3_-15, H-10a (*δ*_H_ 2.74) and from H-8 to H_3_-14 and H-10a (*δ*_H_ 2.75) placed H-8, H_3_-14, and H_3_-15 on the same face. Thus, the relative configuration of **1** was elucidated as 4R*,5R*,8S*. For compound **2**, H_3_-15 was found to show NOE correlations with H_3_-14 and H-10a (*δ*_H_ 2.38), and H-8 showed an NOE correlation with H-10b (*δ*_H_ 2.08) but not with H_3_-14, suggesting that the relative configuration of **2** was 4S*,5S*,8S*. The absolute configurations (AC) of compounds **1** and **2** could be deduced from TDDFT/ECD calculations. A comparison of the experimental ECD spectrum of **1**, **2**， calculated (4*S*,5*S*,8*R*)-**1** and calculated (4*S*,5*S*,8*S*)-**2** (Figure 4) revealed that the AC of **1** and **2** was 4*S*,5*S*,8*R* and 4*S*,5*S*,8*S*, respectively. The NMR calculations were also conducted at the GIAO/mPW1PW91/6-31G (d, p) level. Finally, computed and experimental data were correlated, and the corresponding DP4+ probabilities were estimated. As shown in Figures S1 and S2, the experimental NMR data of compound **1** gave the best match for (4*S*,5*S*,8*R*)-**1**, and compound **2** gave the best match for (4*S*,5*S*,8*S*)-**2**.

The molecular formula of **3**, C_19_H_28_O_4_, was established by HR-ESI-MS, requiring six degrees of unsaturation. The NMR data indicated the presence of two acetyl groups (*δ*_C_/*δ*_H_ 170.1 and 20.9/2.01; *δ*_C_/*δ*_H_ 169.8 and 20.9/2.12), and it was analogous of neolemnane [9]. Two oxygenated methines (*δ*_C_/*δ*_H_ 79.5/4.86; *δ*_C_/*δ*_H_ 73.7/5.67) were found according to the 1D NMR spectra. The placement of two acetyl groups at C-8 and C-9 was confirmed by HMBC correlations from H-8 (*δ*_H_ 5.67) to an acetyl carbon (*δ*_C_ 170.1), and from H-9 (*δ*_H_ 4.86) to another acetyl carbon (*δ*_C_ 169.8). The structure was further confirmed with the assistance of ^1^H-^1^H COSY and HMBC spectra, as shown in Figure 2. The *E* geometry of 6,7-double bond was deduced in the same manner as compounds **1** and **2**. The relative stereochemistry of **3** was also deduced by the key NOE interactions. The observed NOE correlation (Figure 3) between H_3_-14/H_3_-15, H_3_-14/H-9, H-9/8-OAc, and H-8/9-OAc and no NOE correlation with H_3_-14/H-8 demonstrated that 8-OAc, H-9, H_3_-14, and H_3_-15 were close in space while H-8 and 9-OAc were distant, with an antiperiplanar orientation. As a result, the AC of **3** was proposed to be 4*S*,5*S*,8*S*,9*S* according to the NOE correlations and considering biosynthetic properties [9]. Compound **3** was the C-8 and C-9 epimer of lineolemnene C [10].

Compound **4** was also obtained as a colorless oil. The molecular formula was established as C_17_H_26_O_2_ by HR-ESI-MS (*m/z* 285.1824 [M + Na]^+^). The NMR signals (*δ*_C_/*δ*_H_ 118.1/5.17 and 145.9) (Table 1) showed the presence of a trisubstituted double bond. The ^1^H–^1^H COSY spectrum revealed the connectivity of H-1/H-2/H_2_-3/H-4/H_3_-15 and H-6/H-7/H_2_-8/H_2_-9, indicating that it was an aristolane-type sesquiterpenoid [11]. The location of an acetyl group (*δ*_C_/*δ*_H_ 171.1 and 21.5/2.04) at position C-2 (*δ*_C_/*δ*_H_ 68.9/5.35) was revealed by the relevant HMBC correlations. The third ring was a cyclopropane due to the key H_3_-12/C-6/C-7/C-11 and H_3_-13/C-6/C-7/C-11 HMBC correlations. The NOE correlations between H-2/H_3_-15, H_3_-15/H_3_-14, and H-6/H_3_-14 revealed the relative configuration of **4**. ECD calculation was performed (Figure S3) to determine the absolute configuration of compound **4** as 2*S*,4*S*,5*S*,6*R*,7*S*. Compound **4** was named 2-acetoxy-aristolane. The acetoxy group was of natural origin but not artifact, which was confirmed by LC-HRMS analysis of the methanol extract of *Lemnalia* sp. (Figure S37). The protonated- molecular ion peak[M + H]^+^ of **4** was extracted successfully (Figure S37).

**Figure 5:**
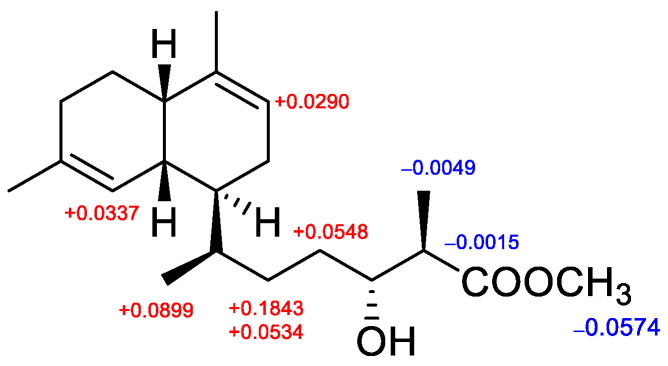
Δ*δ*_H_ values (Δ*δ* (in ppm) = *δ*_S_-*δ*_R_) obtained for (*S*)- and (*R*)-MTPA esters of compound **5** in pyridine-*d*_5_.

Biofloranate A (**5**) was also a colorless oil, and the molecular formula was established as C_21_H_34_O_3_ (HR-ESI-MS: *m/z* 357.2400 [M + Na]^+^). The 1D NMR and HSQC spectra of **5** (Table 2 and Table 3) disclosed two trisubstituted olefinic bonds (*δ*_C_/*δ*_H_ 134.7 and 123.6/5.47; *δ*_C_/*δ*_H_ 136.7 and 121.2/5.39), an acetate group (*δ_C_*/*δ_H_* 176.7 and 51.7/3.71), and an oxygenated methine (*δ*_C_/*δ*_H_ 72.1/3.85), which also indicated a bicyclic structure. A comparison of the ^1^H and ^13^C NMR data of **5** with the aglycone of lemnabourside [12] showed that they had identical ring systems, including stereochemistry, and similar side chains. The planar structure was further established by the analysis of COSY and HMBC correlations. The orientation of 14-OH of **5** was assigned via Mosher’s method. Esterification of **5** with (*R*)- and (*S*)-MTPA chloride occurred at the C-14 hydroxyl group to give (*S*)- and (*R*)-MTPA ester derivatives. The observed Δ*δ*_H_ (*S*-*R*) value distribution pattern (Figure 5, Figures S82 and S83) established the 14*R* absolute configuration for **5**. However, the configuration of C-15 was difficult to determine.

Compound **6** shared the same molecular formula as **5**. The ^1^H and ^13^C NMR spectra of 6 (Table 2 and Table 3) were also similar to those of **5**, except that the oxygenated carbon was quaternary (*δ*_C_ 74.8) instead of a methine. As a result, the hydroxy was connected to C-15 instead of C-14. The orientation of 15-OH present in **3** was hard to determine, leading to the unknown relative configuration of C-15. Compound **6** was given the name biofloranate B.

Biofloranate C (**7**) had a molecular formula of C_21_H_32_O_3_ as determined by HR-ESI-MS data, requiring six degrees of unsaturation. Comparing the molecular formula of **7** with **5** and **6**, two hydrogens were missing, corresponding to the presence of one more trisubstituted double bond (*δ*_C_/*δ*_H_ 130.4 and 146.1/6.88). An oxygenated methylene H-17 (*δ*_C_/*δ*_H_ 57.2/4.33) showed HMBC correlations with C-14, C-15, and C-16. Key H_2_-13/H-14 ^1^H-^1^H COSY correlation and H-14/C-12/C-13/C-15/C-16/C-17 HMBC correlations (Figure 2) suggested that the extra double bond was C-14−C-15. The H-14/16-OCH_3_ NOESY correlation placed H-14 and the methoxy group on the same side (Figure S59). Thus, the structure of **7** was established unambiguously. Biofloranate D (**8**) was obtained as a colorless oil and the formula was assigned as C_20_H_34_O by HR-ESI-MS (*m/z* 313.2336, [M + Na]^+^). The 1D NMR data of **8** (Table 2 and Table 3) also corresponded closely to those of aglycone of lemnabourside [12]. Compared with aglycone, the 1D NMR data revealed the absence of an aldehyde group but the presence of an oxygenated methylene (*δ*_C_/*δ*_Ha_/*δ*_Hb_ 68.4/3.49/3.40). The structure of **8** was also confirmed by ^1^H-^1^H COSY and HMBC correlations, as shown in Figure 2. The AC of C-15 was also difficult to determine.

Euplexaurene D (**9**) was isolated as a white powder, and HR-EI-MS analysis (found 290.2604, M^+^) showed the molecular formula C_20_H_34_O, suggesting four degrees of unsaturation. As the only sp^2^ group, the trisubstituted olefinic bond (*δ*_C_/*δ*_H_ 131.3 and 125.0/5.12) was deduced from the 1D NMR data, indicating the tricyclic structure of **9**. In the ^13^C NMR spectrum (Table 3), 20 carbon signals were assignable to three quaternary carbons, six methines, six methylenes, and five methyls. Comparing the ^13^C NMR spectrum of **9** with that of reported euplexaurene A [13] showed that they have identical ring systems, and the main difference was the position of the hydroxy group. Measuring the 2D NMR spectrum (Figure 2) enabled the assignment of all resonances in the ^1^H NMR and ^13^C NMR spectra (Table 2 and Table 3). The relative configurations of **9** were assigned by NOESY correlations between H_3_-19/H-4, H_3_-19/H_3_-20, H-6/H_3_-19, and H-6/H_3_-18 (Figure 4).

Cneorubin K (**10**) was obtained as a colorless oil, with the molecular formula C_20_H_32_O determined from HR-EI-MS (*m/z* 270.2334 [M − H_2_O]^+^), indicating five degrees of unsaturation. The NMR spectra revealed the presence of a trisubstituted olefinic bond (*δ*_C_/*δ*_H_ 131.1 and 124.8/5.09) and an exocyclic double bond (*δ*_C_/*δ*_H_ 157.8 and 105.5/4.90/4.79), which accounted for two degrees of unsaturation, and the remaining three degrees of unsaturation required a tricyclic system in **10**. The aromadendrane diterpenoid skeleton [14] was established by extensive ^1^H-^1^H COSY and HMBC analysis, as shown in Figure 2. In particular, the exocyclic double bond was C-3/C-19 and oxygenated nonprotonated carbon was C-9 (*δ*_C_ 75.1), which were confirmed by HMBC correlations from H_2_-19 to C-2/C-3 and H_3_-20 to C-10/C-8. The relative stereochemistry of **10** was deduced by the key NOE interactions. The NOE correlations between H-5/H-10, H_3_-20/H-4, H_3_-20/H_2_-7b, H_3_-20/H_3_-18, H_3_-18/H_2_-7b and H_3_-18/H-4 revealed the relative configuration of **10** (Figure 3). ECD calculation was performed (Figure S4) to determine the absolute configuration of compound **10** as 4*S*,5*R*,6*R*,9*R*,10*R*,11*S*.

**Scheme 1:**
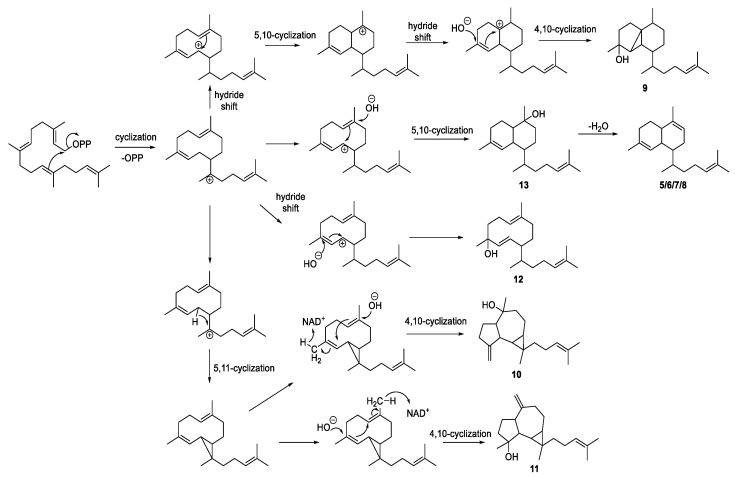
Plausible biosynthetic pathway of diterpenoids **5**–**13**.

According to previously reports, all diterpenoids from *Lemnalia* have a biflorane-based skeleton [7]. In the present study, diterpenoids separated from the *Lemnalia* organism possessed different ring structures, characterized as biflorane, 5,3,6-tricyclic, 5,7,3-tricyclic, and 10-membered carbocyclic skeletons, and were biogenetically related to each other. A geranylgeranyl-PP (GGPP, C20) has been regarded as the same biosynthetic precursor of these diterpenoids, and we further propose biosynthesis for these diterpenoids, as illustrated in Scheme 1.

All of the isolated compounds (**1**–**13**) were evaluated by a wide panel of biological assays, including cytotoxic activity (A-549, HepG2, HeLa, and CCRF-CEM cell lines) and antibacterial (*Bacillus subtilis* and *Staphylococcus aureus*) and antiviral (H1N1 and HSV-1) activity (as shown in Table S1). As a result, lineolemnene J (**3**) showed weak cytotoxicity against the CCRF-CEM cancer cell line (IC_50_ 15.9 µM). Most of the diterpenoids exhibited corresponding antibacterial activity against *Bacillus subtilis* and *Staphylococcus aureus*. Moreover, sesquiterpenes **1**–**4** exhibited anti-H1N1 virus activity with 77.6–100% inhibition at a concentration of 30 µM, and compound **2** exhibited activity with IC_50_ = 5.9 µM.

## 3. Materials and Methods

### 3.1. General Experimental Procedures

Optical rotations and UV spectra were measured on a Jasco P-1010 Polarimeter polarimeter (JASCO, Tokyo, Japan) and ThermoFisher Evolution 201/220 spectrophotometer (Thermo Scientific, Waltham, MA, USA). in MeOH at 20 °C., respectively. NMR spectra were recorded on a Bruker AVANCE NEO 600 spectrometer (BrukerBiospin AG, Fällanden, Germany) with a 5 mm inverse detection triple resonance (H-C/N/D) cryoprobe. ^1^H chemical shifts were referenced to the residual CDCl_3_ (7.24 ppm) and ^13^C chemical shifts were referenced to the CDCl_3_ (77.0 ppm) solvent peaks. High-resolution electrospray ionization mass spectra (HRESIMS) were performed on an Agilent 6230 TOF LC/MS system (Agilent Technologies Inc., Palo Alto, CA, USA) and Thermo Scientific TM Q Exactive PlusTM (Thermo Scientific, Waltham, MA, USA). High-resolution electron impact mass spectra (HREIMS) were recorded on JEOL AccuTOF LC-plus 4G (JEOL, Tokyo, Japan). Reversed-phase HPLC purifications were performed on a Shimadzu LC-20AT HPLC system equipped with a quaternary gradient solvent delivery unit and a photodiode array detector (Shimadzu, Kyoto, Japan), using a semipreparative Cosmosil ODS column (250 mm × 10.0 mm i.d., 5 µm, Cosmosil, Nakalai Tesque Co. Ltd., Kyoto, Japan). Column chromatography was performed on a silica gel (Qingdao Haiyang Chemical Co., Ltd., Qingdao, China) and Cosmosil 75 C_18_ (75 μm, Nakalai Tesque Co. Ltd., Kyoto, Japan).

### 3.2. Animal Material

Soft coral *Lemnalia* sp. (no. XSSC201907) was collected from the Xisha Islands of the South China Sea in April 2019 by scuba at a depth of 10 m. It was identified by Prof. Pingjyun Sung, National Museum of Marine Biology and Aquarium (NMMBA), Taiwan, China.

### 3.3. Extraction and Isolation

A sample of *Lemnalia* sp. (1.9 kg) was freeze-dried and extracted with acetone (1:1, *v/v*, 5 times). Then the extracting solution was concentrated to yield 33.8 g of residue. The residue was suspended in H_2_O (500 mL) and partitioned with Et_2_O (500 mL, 4 times) to yield an Et_2_O solvent extract (25.5 g). The Et_2_O solvent extract was separated on a Sephadex LH-20 column eluted with petroleum ether (PE)-CH_2_Cl_2_-MeOH (2:1:1, *v/v/v*), leading to 4 fractions (Fr.1–Fr.4). Fr.2 (10.4 g) was chromatographed over a silica gel column (PE/EtOAc, from 50:1 to 1:1) to give 9 fractions (Fr.2.1−Fr.2.9). Fr.2.3 was further separated on an ODS column (MeCN/H_2_O, from 50 to 100%) and purified by semipreparative HPLC (MeOH/H_2_O, 97:3, 3 mL/min) to give compound **8** (15.7 mg). Fr.2.5 was also separated on the ODS column (MeCN/H_2_O, from 40 to 100%) to give 4 fractions (Fr.2.4.1–Fr.2.4.4). Fr.2.4.2 was purified by semipreparative HPLC (MeOH/H_2_O, 87:13, 3 mL/min) to give compounds **9** (12.0 mg), 10 (4.1 mg), 11 (7.8 mg), and 13 (7.8 mg). Fr.2.4.3 was purified by semipreparative HPLC (MeOH/H_2_O, 92:8, 3 mL/min) to give compounds **6** (10.0 mg) and 12 (3.5 mg). Fr.2.6 was further separated on the ODS column (MeCN/H_2_O, from 40 to 100%) and purified by semipreparative HPLC (MeOH/H_2_O, 88:12, 3 mL/min) to give compounds **4** (9.8 mg), 5 (10.0 mg), and 7 (5.0 mg). Fr.2.8 was separated on the ODS column (MeCN/H_2_O, from 40 to 80%) and purified by semipreparative HPLC (MeOH/H_2_O, 75:25, 3 mL/min) to give compounds **1** (11.0 mg), 2 (23.5 mg), and 3 (28.0 mg).

Lineolemnene E (**1**): colorless oil; {${\left[\alpha \right]}_{D}^{25}$ −21 (*c* 0.1, MeOH)}; UV (MeOH) *λ*_max_ (log ε) 204 (2.53) nm; ^1^H and ^13^C NMR spectroscopic data, Table 1; HRESIMS *m/z* 299.1616 [M + Na]^+^ (calcd for C_17_H_24_O_3_Na, 299.1623).

Lineolemnene F (**2**): colorless oil; {${\left[\alpha \right]}_{D}^{25}$ +214 (*c* 0.1, MeOH)}; UV (MeOH) *λ*_max_ (log ε) 202 (2.49) nm; ^1^H and ^13^C NMR spectroscopic data, Table 1; HRESIMS *m/z* 299.1618 [M + Na]^+^ (calcd for C_17_H_24_O_3_Na, 299.1623).

Lineolemnene G (**3**): colorless oil; {${\left[\alpha \right]}_{D}^{25}$ +39 (*c* 0.1, MeOH)}; UV (MeOH) *λ*_max_ (log ε) 202 (2.42) nm; ^1^H and ^13^C NMR spectroscopic data, Table 1; HRESIMS *m/z* 343.1880 [M + Na]^+^ (calcd for C_19_H_28_O_4_, 343.1886).

2-Aristolanol acetate (**4**): colorless oil; {${\left[\alpha \right]}_{D}^{25}$ −31 (*c* 0.1, MeOH)}; UV (MeOH) *λ*_max_ (log ε) 203 (2.24) nm; ^1^H and ^13^C NMR spectroscopic data, Table 1; HRESIMS *m/z* 285.1824 [M + Na]^+^ (calcd for C_17_H_26_O_2_, 285.1831).

Biofloranate A (**5**): colorless oil; {${\left[\alpha \right]}_{D}^{25}$ +34 (*c* 0.1, MeOH)}; UV (MeOH) *λ*_max_ (log ε) 202 (2.03) nm; ^1^H and ^13^C NMR spectroscopic data, Table 2 and Table 3; HRESIMS *m/z* 357.2400 [M + Na]^+^ (calcd for C_21_H_34_O_3_, 357.2406).

Biofloranate B (**6**): colorless oil; {${\left[\alpha \right]}_{D}^{25}$ +33 (*c* 0.1, MeOH)}; UV (MeOH) *λ*_max_ (log ε) 201 (2.05) nm; ^1^H and ^13^C NMR spectroscopic data, Table 2 and Table 3; HRESIMS *m/z* 357.2395 [M + Na]^+^ (calcd for C_21_H_34_O_3_, 357.2406).

Biofloranate C (**7**): colorless oil; {${\left[\alpha \right]}_{D}^{25}$ −105 (*c* 0.1, MeOH)}; UV (MeOH) *λ*_max_ (log ε) 202 (2.03) nm; ^1^H and ^13^C NMR spectroscopic data, Table 2 and Table 3; HRESIMS *m/z* 355.2251 [M + Na]^+^ (calcd for C_21_H_32_O_3_, 355.2249).

Biofloranate D (**8**): colorless oil; {${\left[\alpha \right]}_{D}^{25}$ +30 (*c* 0.1, MeOH)}; UV (MeOH) *λ*_max_ (log ε) 202 (2.05) nm; ^1^H and ^13^C NMR spectroscopic data, Table 2 and Table 3; HRESIMS *m/z* 313.2511 (calcd for C_20_H_34_O, 313.2508).

Euplexaurene D (**9**): colorless oil; {${\left[\alpha \right]}_{D}^{25}$ +21 (*c* 0.1, MeOH)}; UV (MeOH) *λ*_max_ (log ε) 201 (1.98) nm; ^1^H and ^13^C NMR spectroscopic data, Table 2 and Table 3; HREIMS *m/z* 290.2604 [M]^+^ (calcd for C_20_H_34_O, 290.2610).

Cneorubin K (**10**): colorless oil; {${\left[\alpha \right]}_{D}^{25}$ −24 (*c* 0.1, MeOH)}; UV (MeOH) *λ*_max_ (log ε) 202 (2.04) nm; ^1^H and ^13^C NMR spectroscopic data, Table 2 and Table 3; HREIMS *m/z* 270.2334 [M-H_2_O]^+^ (calcd for C_20_H_30_, 270.2348).

### 3.4. Computational Methods

Geometry optimization was performed using Gaussian 09W (Gaussian Inc., Wallingford, CT, USA) and the B3LYP functional at the 6-311+G(d,p) level of theory as the method described in a reported article [15].

The theoretical calculation of ^13^C NMR chemical shifts of epimers **1** and **2** were carried out by geometry optimization [16]. The molecular structures were first minimized by MMFF. All torsional angles were systematically varied to search for the global minimum. The molecular mechanics minimized structures were then imported to Gaussian for all subsequent steps. Geometry optimization was conducted at the B3LYP/6-31G (d, p) level of theory. IEF-PCM solvation in chloroform was used for the stereoisomers. Chloroform was the solvent in which experimental NMR spectra were acquired. ^13^C NMR shielding constants were calculated at the GIAO/mPW1PW91/6-31G (d, p) level, with IEF-PCM solvation in the chloroform solvent.

### 3.5. Cytotoxic Activity Assay

Cell lines were purchased from the American Type Cultural Collection (ATCC, Manassas, VA, USA). The cytotoxicity of the compounds was evaluated against the A549, HepG2, Hela and CCRF-CEM with the Cell Counting Kit-8 (CCK-8) as the reported method [17]. Chidamide was used as a positive control.

### 3.6. Antibacterial Assays

The antibacterial activities were evaluated with the broth dilution assay [18]. Bacterial strains, *Bacillus subtilis* (CMCC (B) 63501), *Staphylococcus aureus* (CMCC (B) 26003) were used, and penicillin G as a positive control.

### 3.7. Antiviral Assays

The antiviral activities against the influenza A virus (H1N1) and (HSV-1) were evaluated as the literature reported [19]. Acyclovir was used as a positive control.

## 4. Conclusions

Marine invertebrates have been regarded as an important source of bioactive secondary metabolites. The acetone extracts of soft coral *Lemnalia* sp. (no. XSSC201907) were chemically investigated, resulting in the discovery of four new sesquiterpenes (**1**–**4**) and six new diterpenoids (**5**–**10**), along with three known related diterpenoids. Most of the isolated diterpenes were found to exhibit moderate antimicrobial activity against *Bacillus subtilis* and *Staphylococcus aureus*. Sesquiterpene lineolemnene J (**3**) showed weak cytotoxicity against the CCRF-CEM cancer cell line. Lineolemnene (**2**) exhibited medium inhibitory activity against influenza A H1N1 virus.

## Figures and Tables

**Table 1:** NMR spectroscopic data (^1^H 600 MHz, ^13^C 150 MHz, CDCl_3_) for compounds **1**–**4.**

	1		2		3		4	
Position	*δ*_H_, Mult.	*δ*_C_, Type	*δ*_H_, Mult.	*δ*_C_, Type	*δ*_H_, Mult.	*δ*_C_, Type	*δ*_H_, Mult.	*δ*_C_, Type
1	5.43, m	123.4, CH	5.49, m	125.1, CH	5.46, m	123.9, CH	5.17, br.s	118.1, CH
2a	2.07, m	26.1, CH_2_	2.09, m	26.0, CH_2_	2.06, m	26.0, CH_2_	5.35, m	68.9, CH
2b	2.01, m	—	2.09, m	—	2.06, m	—	—	—
3a	1.49, m	26.6, CH_2_	1.51, m	26.1, CH_2_	1.48, m	26.7, CH_2_	1.93, m	42.6, CH_2_
3b	1.37, m	—	1.51, m	—	1.41, m	—	1.57, m	—
4	2.26, ov	36.0, CH	2.41, m	33.2, CH	2.28, m	35.3, CH	1.95, m	37.9, CH
5	—	46.2, C	—	45.3, C	—	43.6, C	—	51.7, C
6	5.82, s	138.6, CH	5.22, s	147.5, CH	5.33, s	132.4, CH	0.85, d (6.5)	38.8, CH
7	—	127.8, C	—	128.0, C	—	137.4, C	1.22, br.t (6.3)	32.4, CH
8a	5.66, s	85.5, CH	5.16, s	84.8, CH	5.67, d (6.9)	73.7, CH	2.01, m	26.8, CH_2_
8b	—	—	—	—	—	—	1.74, m	—
9a	—	201.2, C	—	203.2, C	4.86, dd (9.9, 7.3)	79.5, CH	1.90, m	33.0, CH_2_
9b	—	—	—	—	—	—	1.59, m	—
10a	2.75, td (12.6, 3.8)	40.8, CH_2_	2.38, m	42.9, CH_2_	1.74, m	32.2, CH_2_	—	145.9, C
10b	2.16, m	—	2.08, m	—	1.57, dd (15.1, 9.3)	—	—	—
11a	2.59, m	32.6, CH_2_	2.33, m	34.3, CH_2_	2.22, dd (15.2, 9.4)	34.6, CH_2_	—	20.3, C
11b	2.30, ov	—	2.32, m	—	2.05, m	—	—	—
12	—	140.4, C	—	142.4, C	—	143.8, C	1.00, s	28.8, CH_3_
13	1.51, s	13.0, CH_3_	2.01, s	21.2, CH_3_	1.72, s	15.5, CH_3_	1.14, s	17.1, CH_3_
14	1.13, s	21.4, CH_3_	1.03, s	21.0, CH_3_	1.05, s	22.1, CH_3_	1.76, s	20.4, CH_3_
15	0.99, d (6.9)	17.8, CH_3_	0.95, d (6.9)	17.5, CH_3_	0.91, d (6.9)	17.6, CH_3_	1.07, d (7.1)	17.0, CH_3_
7-OAc	—	—	—	—	—	—	—	171.1, C
	—	—	—	—	—	—	2.04, s	21.5, CH_3_
8-OAc	—	170.1, C	—	170.6, C	—	170.1, C	—	—
	2.18, s	20.8, CH_3_	2.23, s	20.9, CH_3_	2.01, s	20.9, CH_3_	—	—
9-OAc	—	—	—	—	—	169.8, C	—	—
	—	—	—	—	2.12, s	20.9, CH_3_	—	—

^ov^ Overlapped signals, — absence of value.

**Table 2:** ^1^H NMR (600 MHz) spectroscopic data for compounds **5**–**10**.

Position	5	6	7	8	9	10
	*δ*_H_, Mult.	*δ*_H_, Mult.	*δ*_H_, Mult.	*δ*_H_, Mult.	*δ*_H_, Mult.	*δ*_H_, Mult.
1a	1.85, m	1.84, m	1.78, m	1.84, ov	1.84, m	1.88, ov
1b	1.36, ov	1.36, m	1.36, ov	1.37, ov	1.52, ov	1.47, qd (11.5, 8.3)
2a	1.98, ov	1.97, m	1.99, ov	1.98, ov	1.53, ov	2.40, m
2b	1.92, ov	1.91, ov	1.95, ov	1.91, ov	1.35, ov	2.31, m
4	5.47, br.s	5.47, br.s	5.45, br.s	5.48, br.s	0.87, ov	1.71, ov
5	2.04, dd (9.3, 4.7)	2.02, m	2.04, ov	2.04, m	0.80, ov	0.54, br.t (9.5)
6	1.52, ov	1.48, m	1.51, dt (7.4, 3.1)	1.51, m	1.14, m	0.66, td (10.9, 10.1, 6.5)
7a	1.78, ov	1.75, ov	1.83, ov	1.84, ov	1.33, ov	1.85, ov
7b	1.78, ov	1.75, ov	1.78, ov	1.77, ov	0.83, ov	0.89, m
8a	5.39, br.s	5.39, br.s	5.39, br.s	5.40, br.s	1.57, ov	1.75, dd (12.9, 7.1)
8b	—	—	—	—	0.51, m	1.60, ov
9	—	—	—	—	1.64, dd (11.9, 6.0)	—
10	1.95, ov	1.93, ov	1.94, ov	1.94, ov	—	1.92, td (11.1, 6.5)
11	1.78, ov	1.78, ov	1.83, ov	1.79, ov	1.51, ov	—
12a	1.38, ov	1.18, ov	1.36, ov	1.30, ov	1.46, m	1.26, m
12b	1.22, ov	1.18, ov	1.36, ov	1.21, ov	1.21, m	1.16, m
13a	1.51, m	1.69, ov	2.27, m	1.19, m	2.04, dd (13.9, 6.3)	2.06, m
13b	1.36, ov	1.62, ov	2.27, m	1.19, m	1.92, dq (14.5, 7.5)	2.06, m
14a	3.85, dd (8.0, 3.8)	1.39, ov	6.88, t (7.8)	1.37, ov	5.12, br.t (6.2)	5.09, t (7.1)
14b	—	1.15, m	—	1.08, ov	—	—
15	2.54, qd (7.2, 3.5)	—	—	1.61, m	—	—
16a	—	—	—	3.40, dd (10.4, 6.6)	1.61, s	1.60, s
16b	—	—	—	3.49, dd (10.5, 5.9)	—	—
17	1.18, d (7.2)	1.39, s	4.33, br.s	0.90, d (6.7)	1.69, s	1.67, s
18	0.83, d (6.8)	0.79, d (6.7)	0.84, d (6.9)	0.81, d (6.8)	0.90, d (6.7)	1.04, s
19a	1.68, s	1.69, s	1.70, s	1.69, s	1.28, s	4.90, s
19b	—	—	—	—	—	4,79, s
20	1.68, s	1.68, s	1.69, s	1.69, s	0.93, d (6.4)	1.12, s
OMe	3.71, s	3.78, s	3.78, s	—	—	—

^ov^ Overlapped signals, — absence of value.

**Table 3:** ^13^C NMR (150 MHz) spectroscopic data for compounds **5**–**10.**

Position	5	6	7	8	9	10
	*δ*_C_, Type	*δ*_C_, Type	*δ*_C_, Type	*δ*_C_, Type	*δ*_C_, Type	*δ*_C_, Type
1	24.6, CH_2_	24.6, CH_2_	24.5, CH_2_	24.7, CH_2_	29.5, CH_2_	26.3, CH_2_
2	30.8, CH_2_	30.8, CH_2_	30.7, CH_2_	30.8, CH_2_	36.3, CH_2_	32.1, CH_2_
3	134.7, C	134.6, C	134.8, C	134.5, C	80.4, C	157.8, C
4	123.6, CH	123.8, CH	123.4, CH	123.8, CH	39.0, CH	41.6, CH
5	36.3, CH	36.4, CH	36.2, CH	36.3, CH	23.1, CH	32.7, CH
6	39.1, CH	38.9, CH	38.7, CH	39.0, CH	42.5, CH	26.6, CH
7	24.6, CH_2_	24.6, CH_2_	24.5, CH_2_	24.6, CH_2_	25.3, CH2	20.4, CH_2_
8	121.2, CH	121.4, CH	121.0, CH	121.4, CH	31.7, CH_2_	44.6, CH_2_
9	136.7, C	136.7, C	136.6, C	136.6, C	30.9, CH	75.1, C
10	39.5, CH	39.6, CH	39.4, CH	39.5, CH	33.6, C	57.9, CH
11	31.8, CH	35.6, CH	31.6, CH	31.7, CH	38.3, CH	23.2, C
12	31.4, CH_2_	31.6, CH_2_	34.5, CH_2_	35.9, CH_2_	34.6, CH_2_	43.2, CH_2_
13	31.9, CH_2_	21.7, CH_2_	26.6, CH_2_	25.0, CH_2_	26.1, CH_2_	25.4, CH_2_
14	72.1, CH	40.6, CH_2_	146.1, CH	33.5, CH_2_	125.0, CH	124.8, CH
15	44.0, CH	74.8, C	130.4, C	35.8, CH	131.3, C	131.1, C
16	176.7, C	177.9, C	168.0, C	68.4, CH_2_	17.7, CH_3_	17.6, CH_3_
17	10.6, CH_3_	26.0, CH_3_	57.2, CH_2_	16.6, CH_3_	25.7, CH_3_	25.7, CH_3_
18	13.2, CH_3_	13.3, CH_3_	13.1, CH_3_	13.3, CH_3_	16.1, CH_3_	13.7, CH_3_
19	23.9, CH_3_	24.0, CH_3_	23.8, CH_3_	24.0, CH_3_	27.9, CH_3_	105.5, CH_2_
20	21.7, CH_3_	21.8, CH_3_	21.6, CH_3_	21.7, CH_3_	18.8, CH_3_	20.5, CH_3_
OMe	51.7, CH_3_	52.7, CH_3_	51.7, CH_3_			

## Data Availability

The data presented in this study are available in the Supplementary Materials file associate with this article.

## Supplementary Materials

The following are available online https://www.mdpi.com/article/10.3390/md19060294/s1. Table S1 Antibacterial and antiviral activity of the isolated compounds **1**–**13**; Figures S1–S2 Calculated and experimental ECD spectra of compounds **4** and **10**; Figures S3–S81 HRESIMS, 1D and 2D NMR spectra of all new compounds **1**–**10**; Figures S82–S83 ^1^H-NMR for (*S*)-MTPA and (*R*)-MTPA esters of compound **5**; Figures S84–S89 1D NMR spectra of known compounds **11**–**13**.
