# Supplementary material for: Antimicrobial Terpenoids from South China Sea Soft Coral Lemnalia sp
Source: Mar Drugs. 2021 May 22;19(6):294. doi: 10.3390/md19060294 (PMC8224568; doi:10.3390/md19060294)
Supplement: Supplementary file 1 [file marinedrugs-19-00294-s001.zip › marinedrugs-1214830-supplementary.pdf]

Supplementary Materials

Antimicrobial Terpenoids from South China Sea Soft Coral *Lemnalia* sp.

Xia Yan [1], Han Ouyang [2,*], Wei Wang [3], Jing Liu [1], Te Li [1], Bin Wu [4], Xiaojun Yan [1] and Shan He [1,*]

[1] Li Dak Sum Yip Yio Chin Kenneth Li Marine Biopharmaceutical Research Center, Department of Marine Pharmacy, College of Food and Pharmaceutical Sciences, Ningbo University, Ningbo 315800, Zhejiang, China; yanxia@nbu.edu.cn (X.Y.); 1397930711@qq.com (J.L.); telinbu@163.com (T.L.); yanxiaojun@nbu.edu.cn (X.Y.)

[2] Institute of Drug Discovery Technology, Ningbo University, Ningbo 315211, Zhejiang, China

[3] Key Laboratory of Marine Drugs, Chinese Ministry of Education; School of Medicine and Pharmacy, Ocean University of China, Qingdao 266003, Shandong, China; wwwakin@ouc.edu.cn (W.W.)

[4] Ocean College, Zhejiang University, Hangzhou, 310058, Zhejiang, China; wubin@zju.edu.cn (B.W.)

* Correspondence: ouyanghan@nbu.edu.cn (H.O.); heshan@nbu.edu.cn (S.H.); Tel.: +86-574-87604382 (S.H.); +86-574-87609771(H.O.)

Content

1. Antibacterial and Antiviral Activity of the Isolated Compounds 1-13 3
2. Regression analysis of experimental vs calculated ^{13}C NMR chemical shifts of compound 1 and 2 3
3.Calculated ECD spectrum of (2*S*,4*S*,5*S*,6*R*,7*S*)-4 and experimental ECD spectrum of 4.. 4
4. Calculated ECD spectrum of (1*R*,5*S*,6*R*,7*R*,10*R*,11*S*)-10 and experimental ECD spectrum of 10...5
5. Spectroscopic data for lineolemnene E (1) 5
6. Spectroscopic data for lineolemnene F (2) 9
7. Spectroscopic data for lineolemnene G (3)..13
8. Spectroscopic data for 2-acetoxy-aristolane (4) 18
9. Spectroscopic data for biofloranate A (5) 23
10. Spectroscopic data for biofloranate B (6)..28
11. Spectroscopic data for biofloranate C (7)..31
12. Spectroscopic data for biofloranate D (8) 35
13. Spectroscopic data for euplexaurene D (9) 39
14. Spectroscopic data for cneorubin K (10)...44
15. ^{1}H-NMR for (*S*)-MTPA and (*R*)-MTPA esters of compound 5 in pyridine-d_5..49
16. Spectroscopic data for known compound cneorubin X (11) 50
17. Spectroscopic data for known compound obscuronatin (12) 51
18. Spectroscopic data for known compound dictyotin B (13)..............................52

1. Antibacterial and Antiviral Activity of the Isolated Compounds 1-13

Table S1. Antibacterial and antiviral activity of the isolated compounds **1-13**.

No.	MIC (against *Staphylococcus aureus,* μg/mL)	MIC (against *Bacillus subtilis,* μg/mL)	Inhibition rate at 30 μM (against H1N1)	Inhibition rate at 50 μM (against HSV-1)
**1**	> 128	> 128	84.50%	0.60%
**2**	> 128	> 128	100.00%	5.10%
**3**	> 128	> 128	77.60%	32.10%
**4**	> 128	> 128	88.50%	0.00%
**5**	8	8	2.20%	0.00%
**6**	4	16	5.40%	0.00%
**7**	4	16	21.70%	0.00%
**8**	16	8	15.60%	0.00%
**9**	> 128	> 128	2.20%	13.70%
**10**	16	8	0.00%	0.00%
**11**	> 128	16	49.40%	50%
**12**	> 128	16	2.70%	0.00%
**13**	> 128	8	1.40%	0.00%
Penicillin G	1	1	——	——
Acyclovir	——	——	97.00%	93.70%

2. Regression analysis of experimental vs calculated ^{13}C NMR chemical shifts of compound 1 and 2

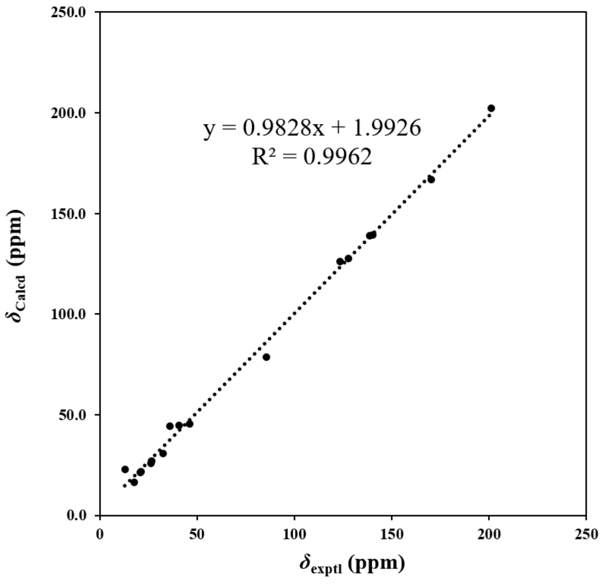

Figure S1. Regression analysis of experimental vs calculated ^{13}C NMR chemical shifts of (4S,5S,8R)-**1** at the GIAO/mPW1PW91/6-31G (d, p) level using DP4+ method. The linear fitting is shown as dashed line

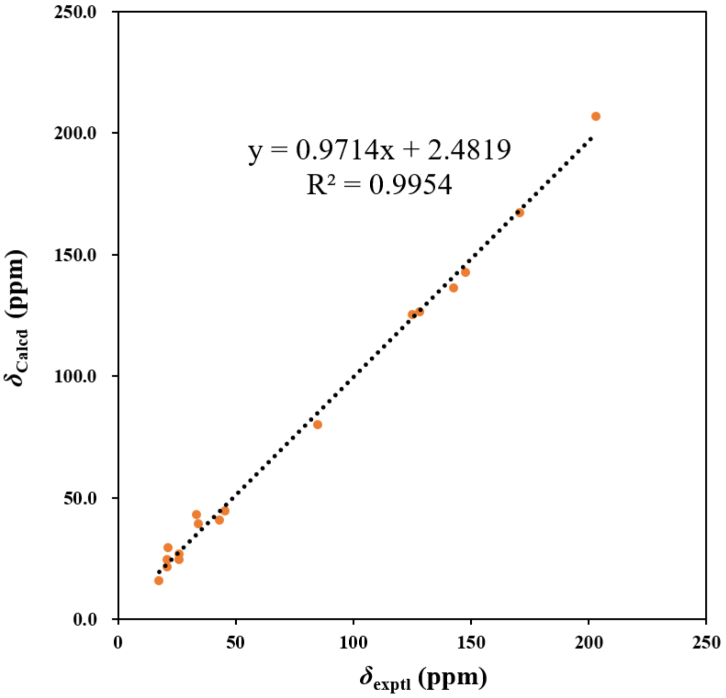

Figure S2. Regression analysis of experimental vs calculated ^{13}C NMR chemical shifts of (4S,5S,8S)-**2** at the GIAO/mPW1PW91/6-31G (d, p) level using DP4+ method. The linear fitting is shown as dashed line

3. Calculated ECD spectrum of (2*S*,4*S*,5*S*,6*R*,7*S*)-4 and experimental ECD spectrum of 4

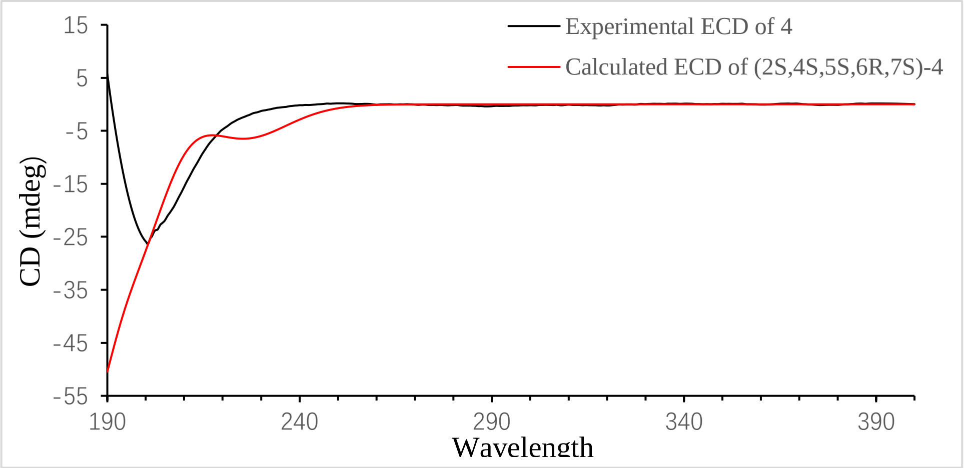

Figure S3. Calculated ECD spectrum of (2*S*,4*S*,5*S*,6*R*,7*S*)-**4** and experimental ECD spectrum of **4**

4. Calculated ECD spectrum of (1*R*,5*S*,6*R*,7*R*,10*R*,11*S*)-10 and experimental ECD spectrum of 10

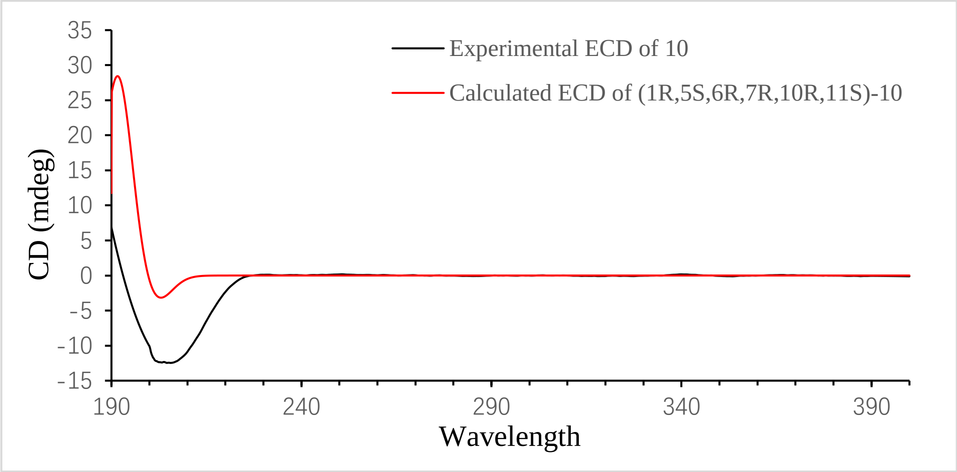

Figure S4. Calculated ECD spectrum of (1*R*,5*S*,6*R*,7*R*,10*R*,11*S*)-**10** and experimental ECD spectrum of **10**

5. Spectroscopic data for lineolemnene E (1)

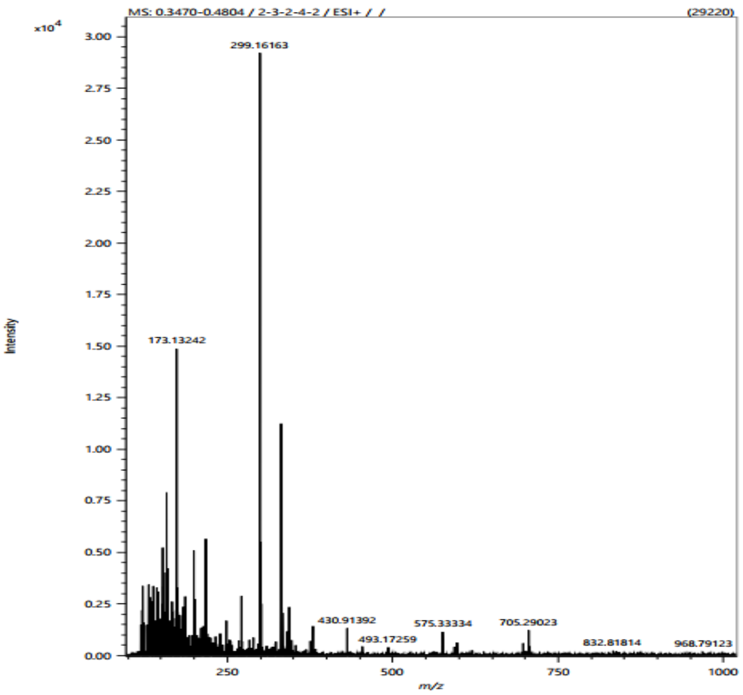

Figure S5. HRESIMS spectrum of lineolemnene E (**1**)

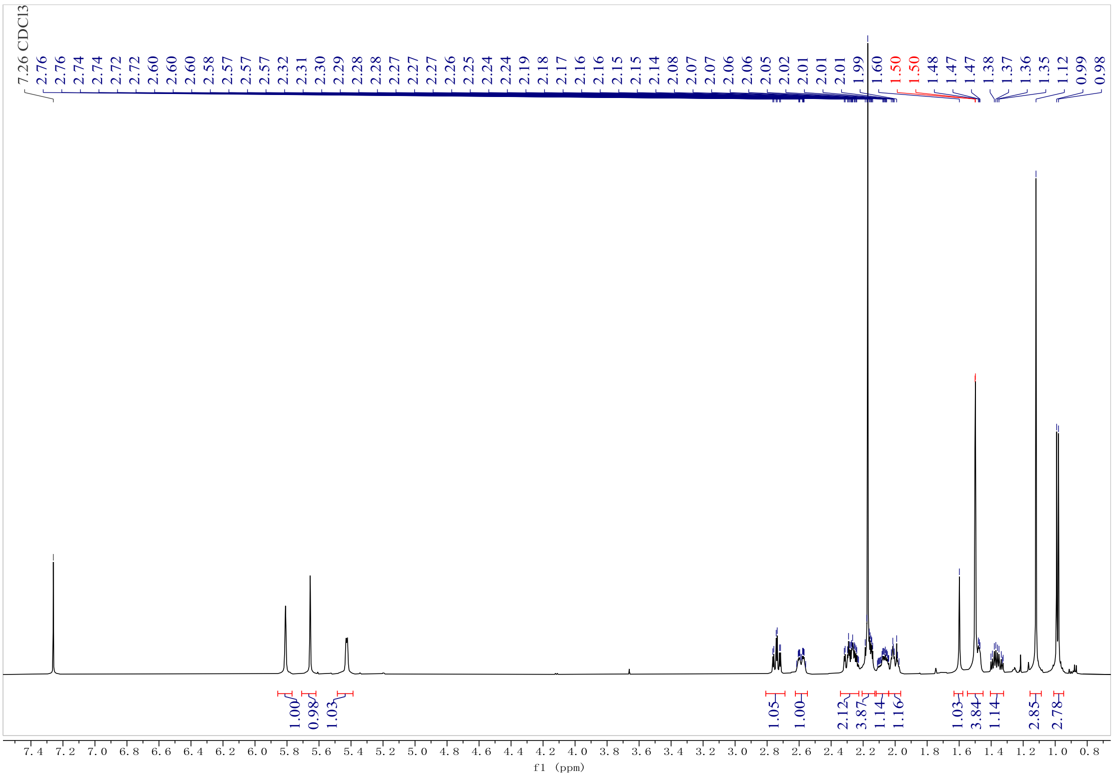

Figure S6. ^{1}H-NMR spectrum of lineolemnene E (**1**) in $CDCl_3$

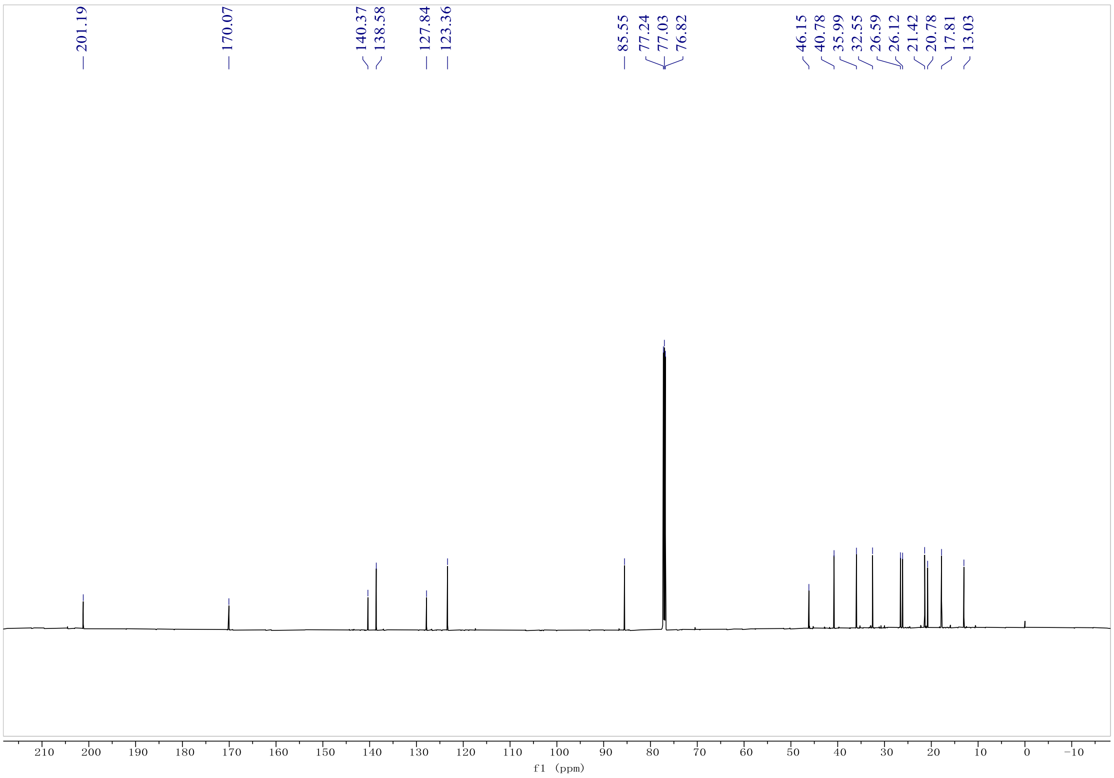

Figure S7. ^{13}C-NMR spectrum of lineolemnene E (**1**) in $CDCl_3$

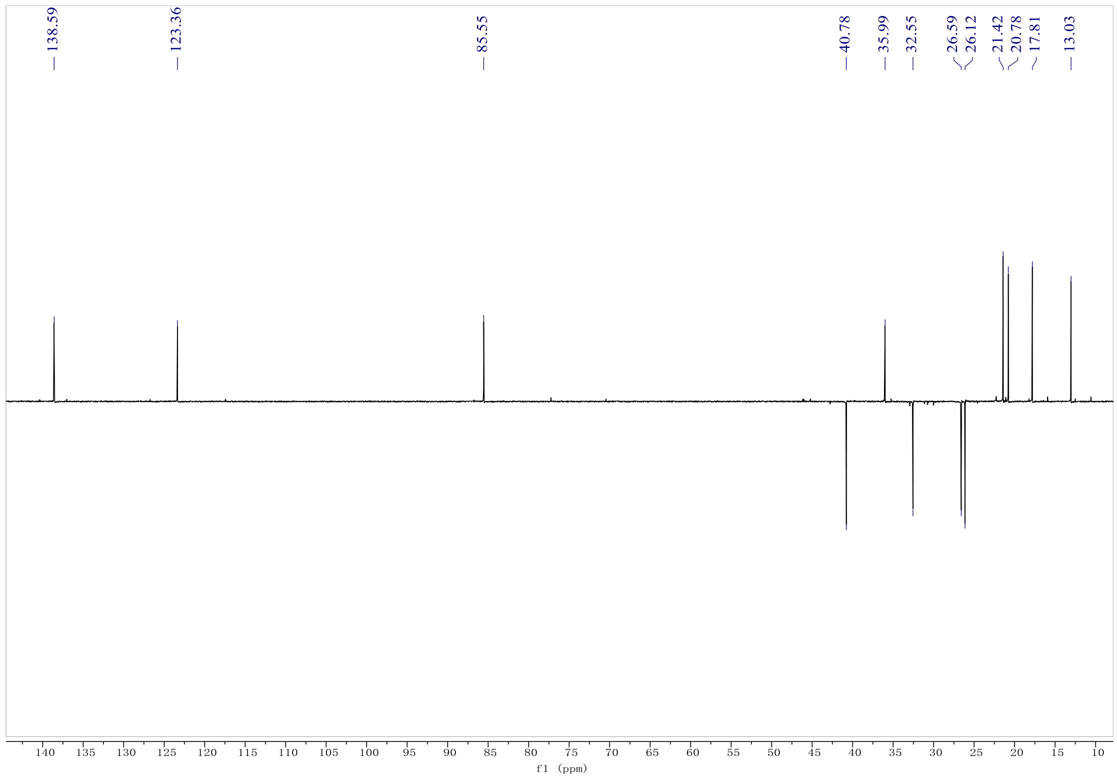

Figure S8. Dept 135° spectrum of lineolemnene E (**1**) in $CDCl_3$

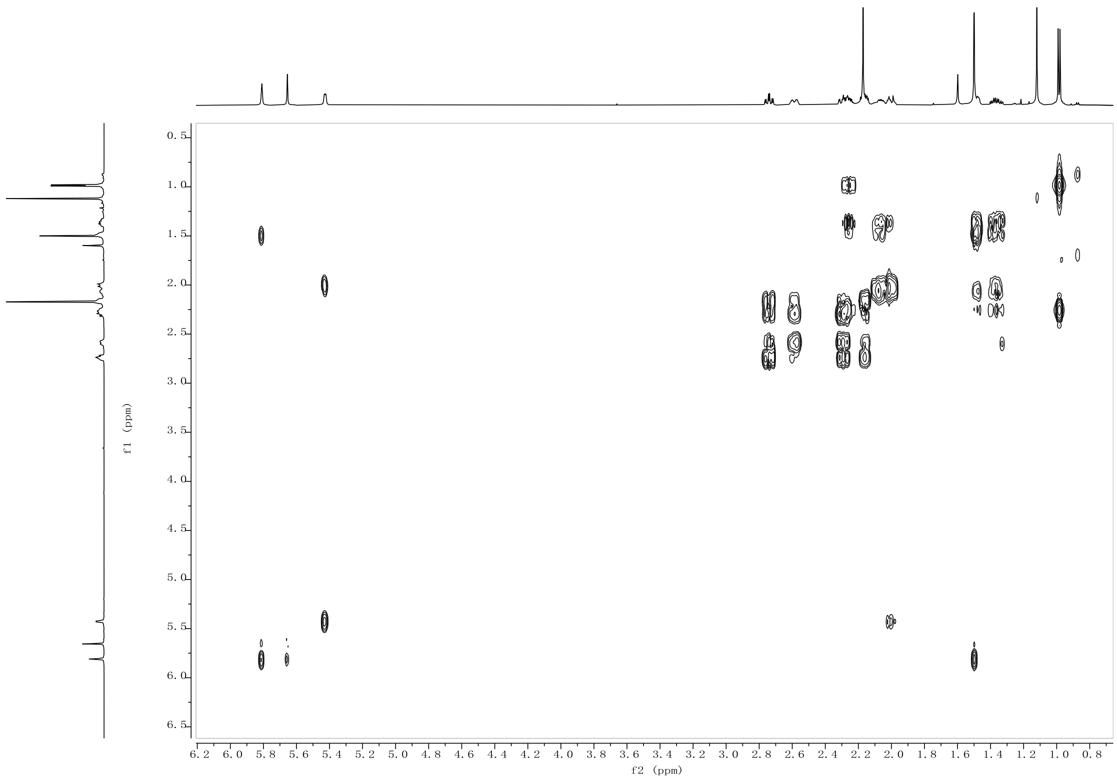

Figure S9. ^1H-^1H COSY spectrum of lineolemnene E (**1**) in $CDCl_3$

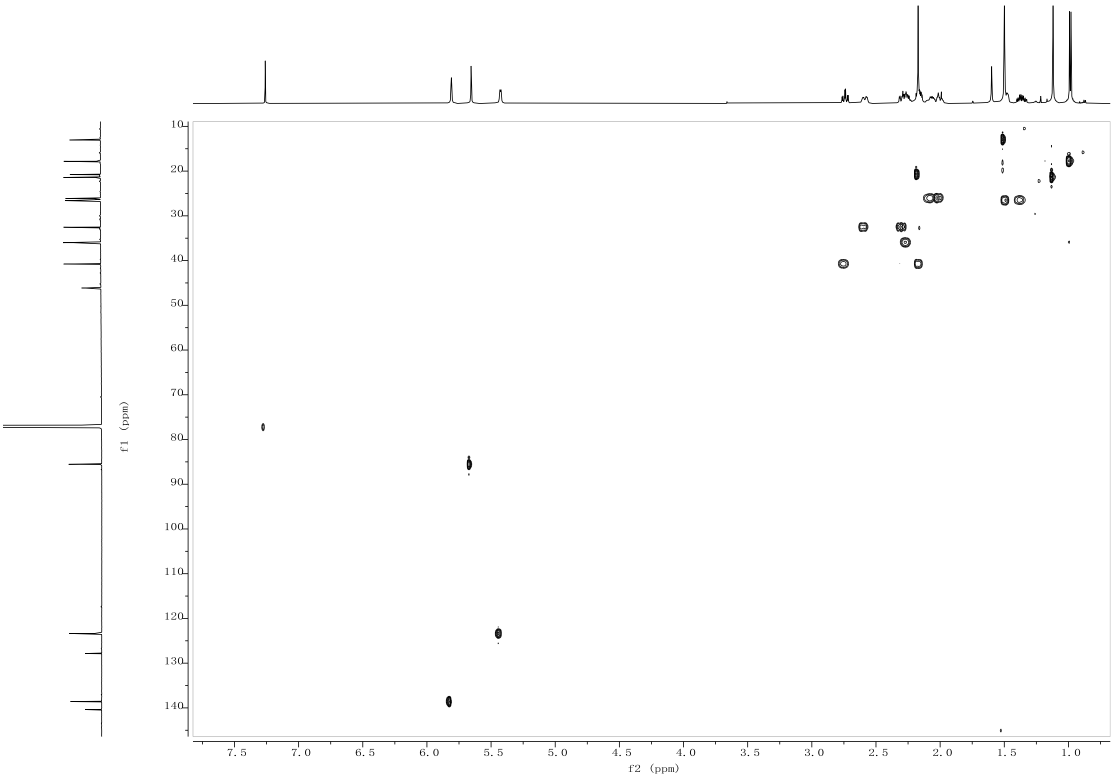

Figure S10. HSQC spectrum of lineolemnene E (**1**) in $CDCl_3$

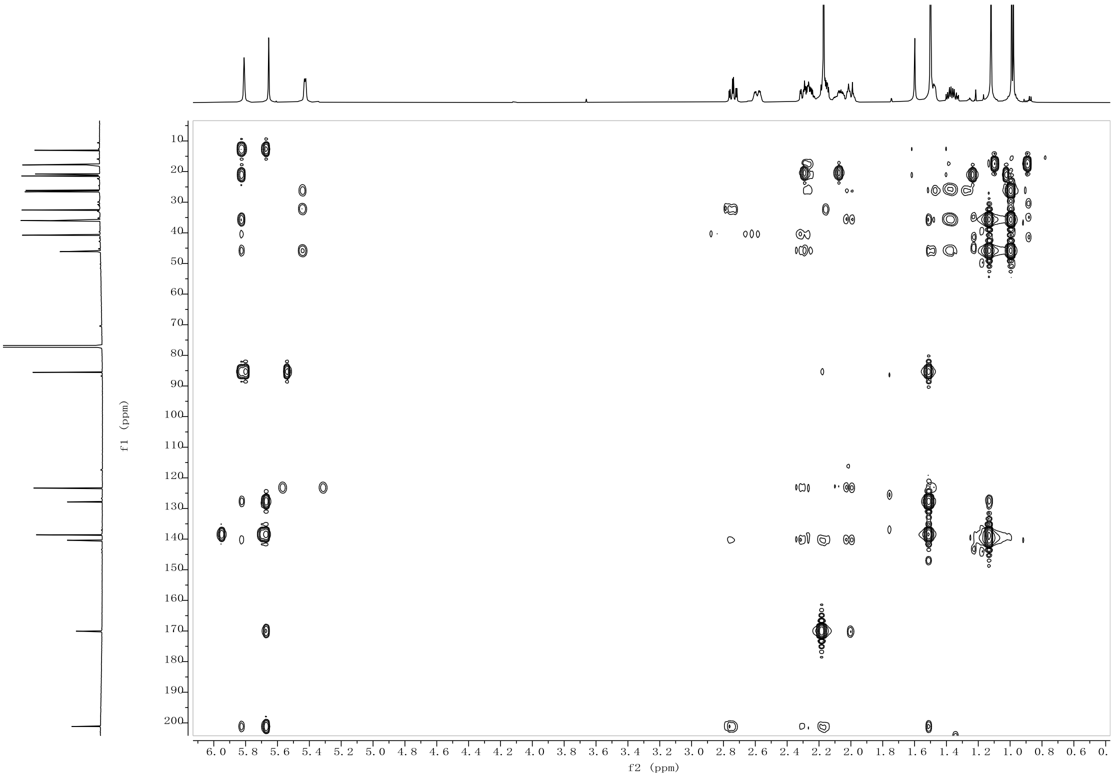

Figure S11. HMBC spectrum of lineolemnene E (**1**) in $CDCl_3$

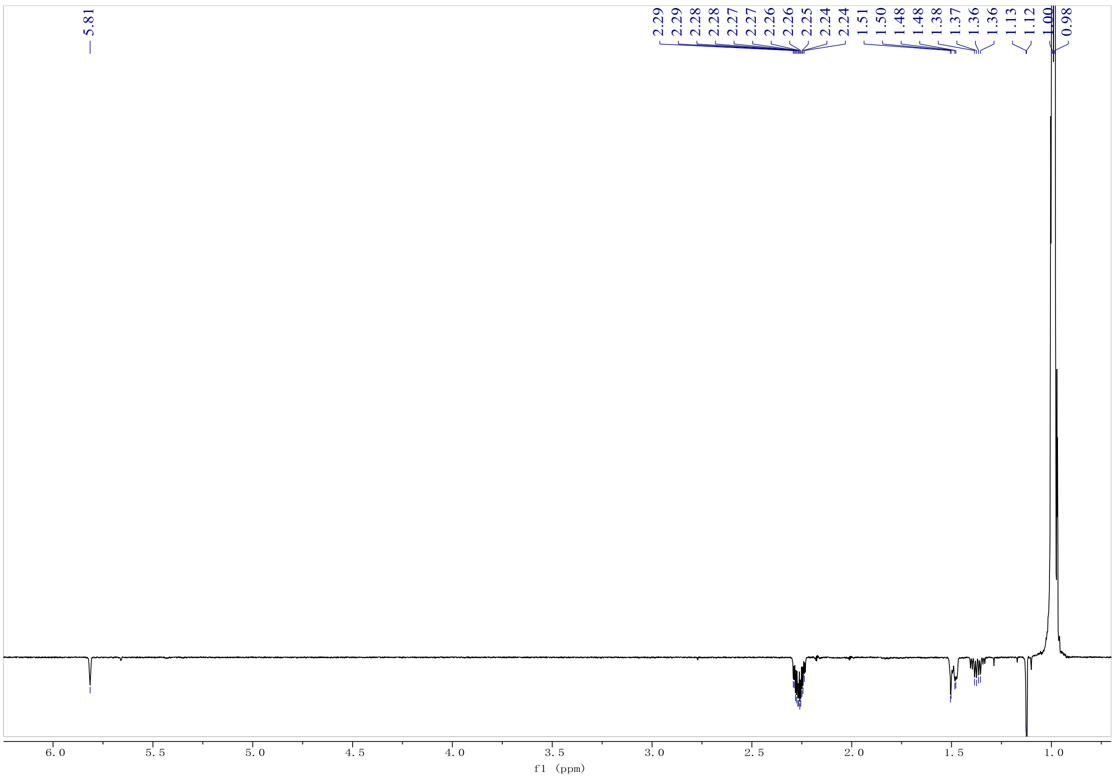

Figure S12. NOESY spectrum of lineolemnene E (**1**) in $CDCl_3$

6. Spectroscopic data for lineolemnene F (2)

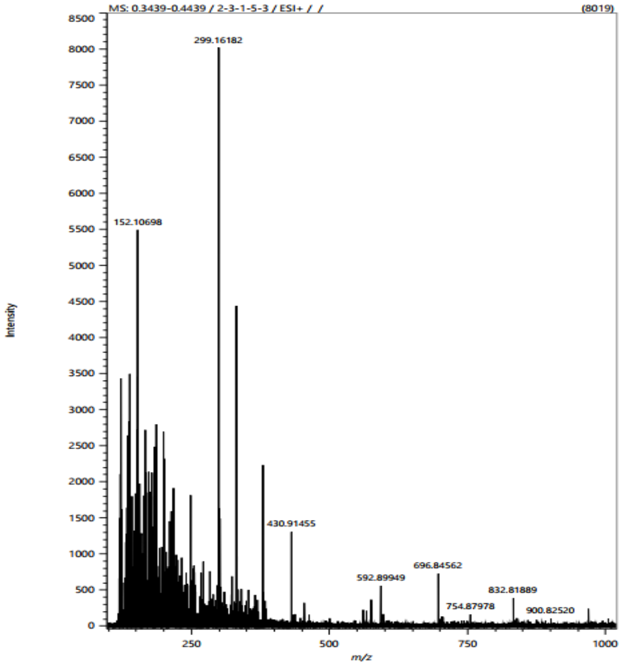

Figure S13. HRESIMS spectrum of lineolemnene F (**2**)

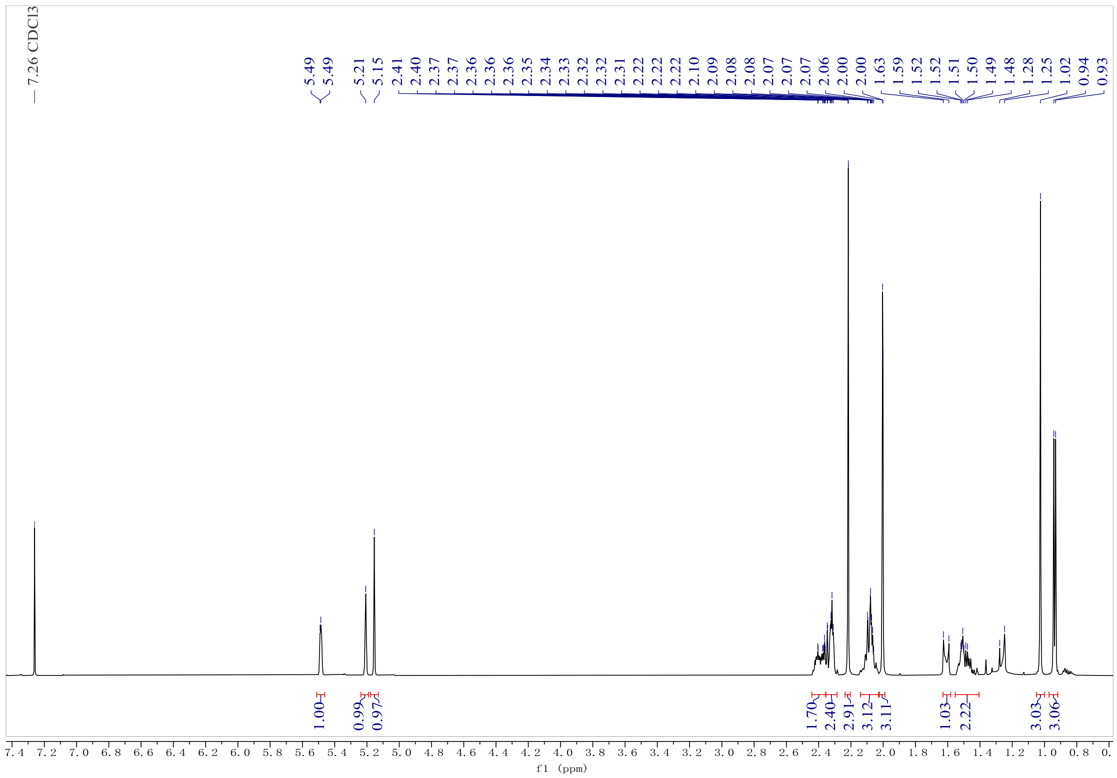

Figure S14. ^{1}H-NMR spectrum of lineolemnene F (**2**) in $CDCl_3$

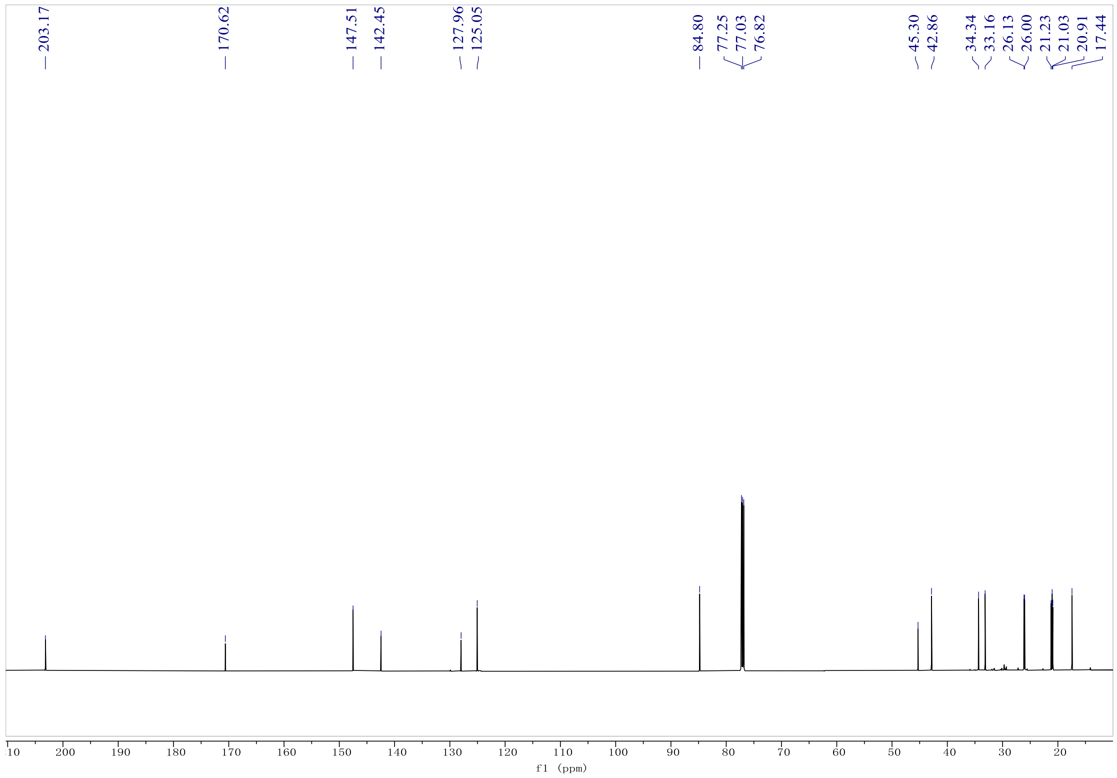

Figure S15. ^{13}C-NMR spectrum of lineolemnene F (**2**) in $CDCl_3$

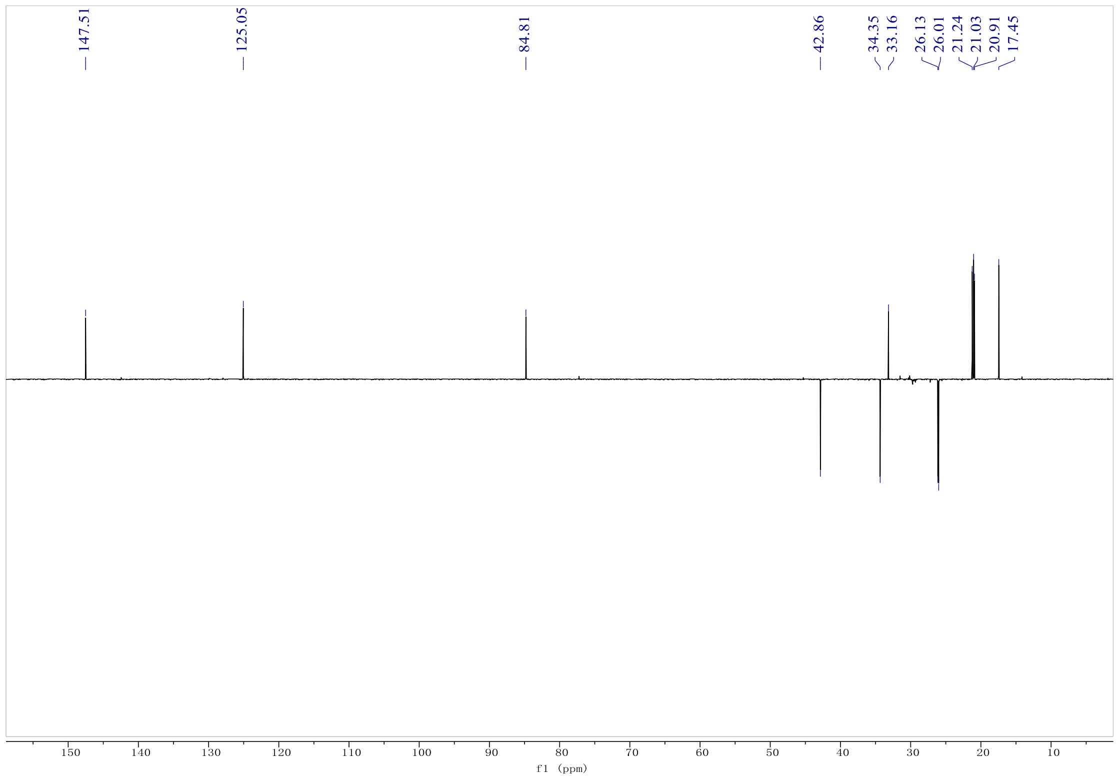

Figure S16. Dept 135° spectrum of lineolemnene F (**2**) in $CDCl_3$

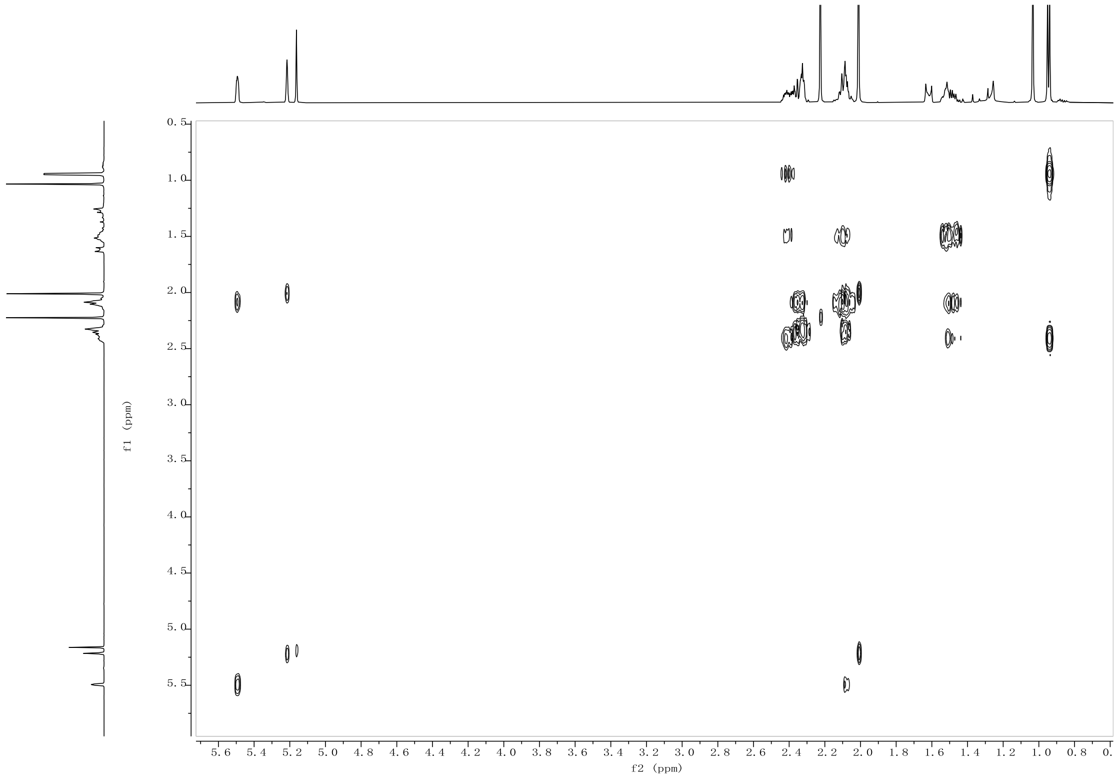

Figure S17. ^{1}H-^{1}H COSY spectrum of lineolemnene F (**2**) in $CDCl_3$

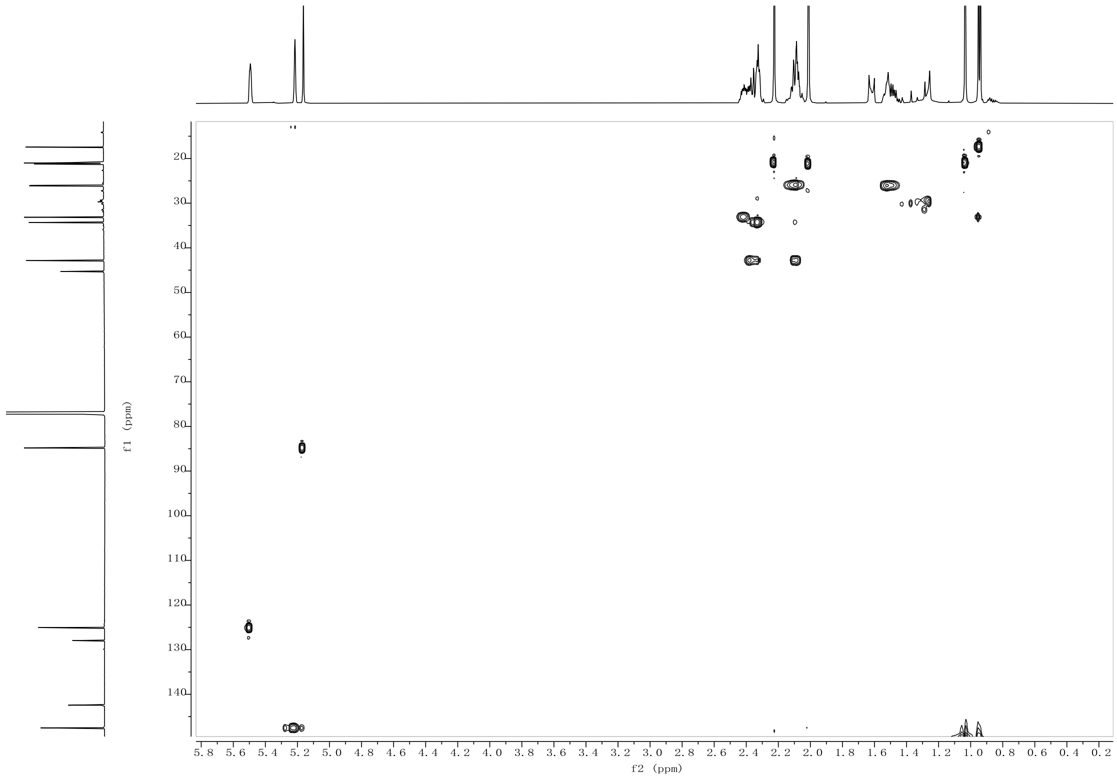

Figure S18. HSQC spectrum of lineolemnene F(**2**) in $CDCl_3$

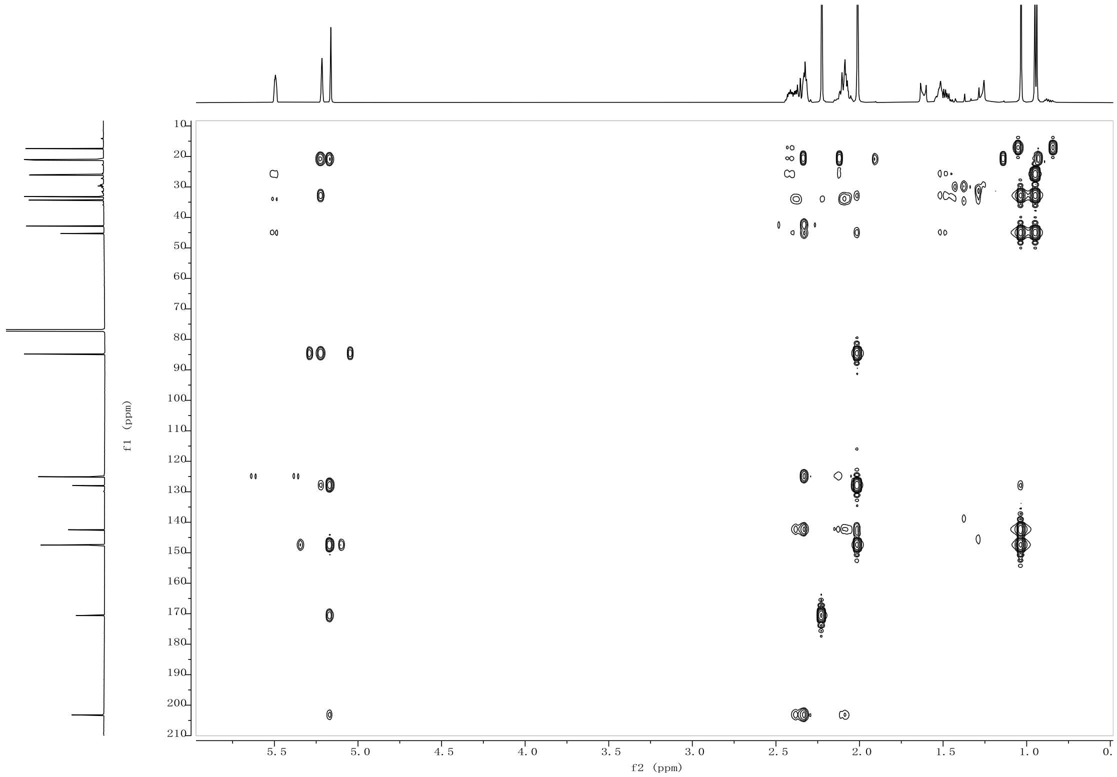

Figure S19. HMBC spectrum of lineolemnene F (**2**) in $CDCl_3$

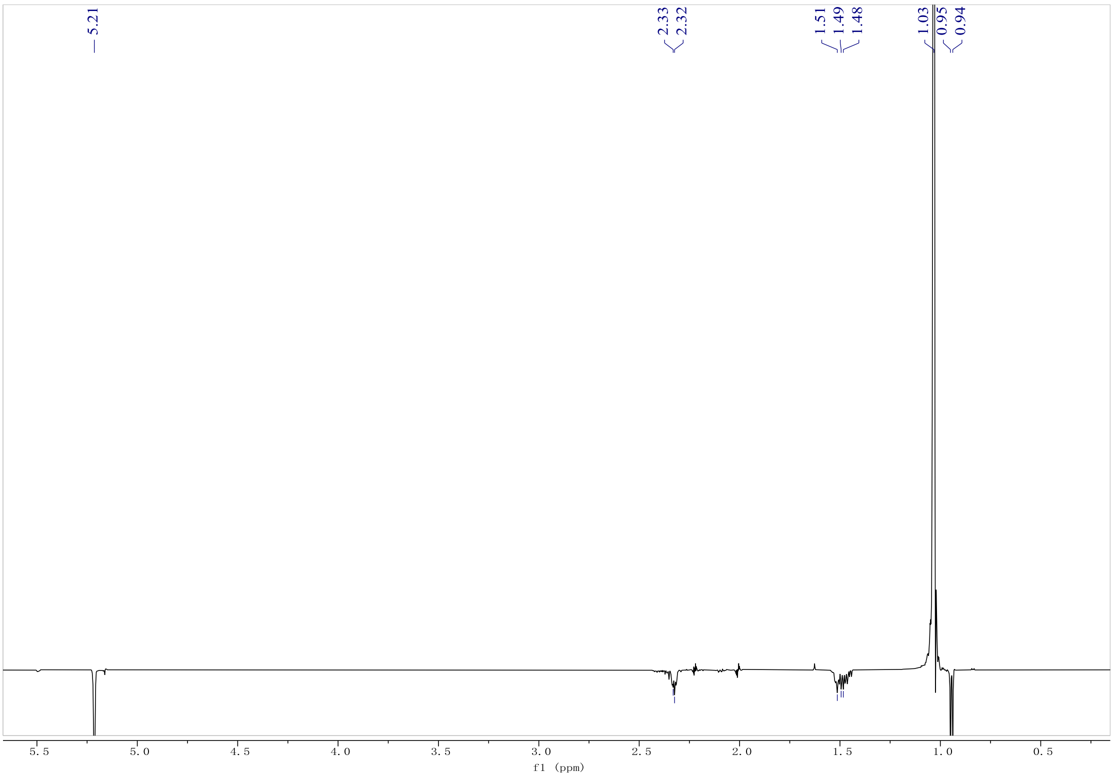

Figure S20. NOESY spectrum of lineolemnene F (**2**) in $CDCl_3$

7. Spectroscopic data for lineolemnene G (3)

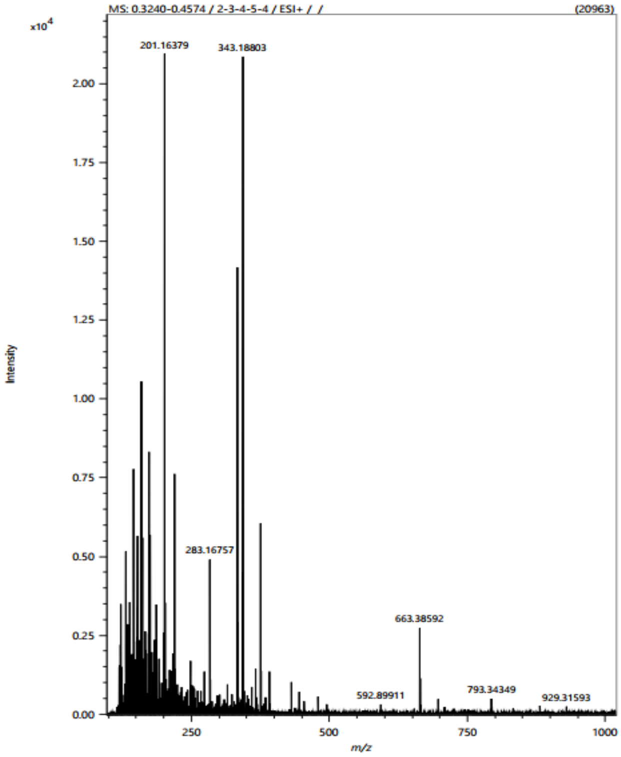

Figure S21. HRESIMS spectrum of lineolemnene G (**3**)

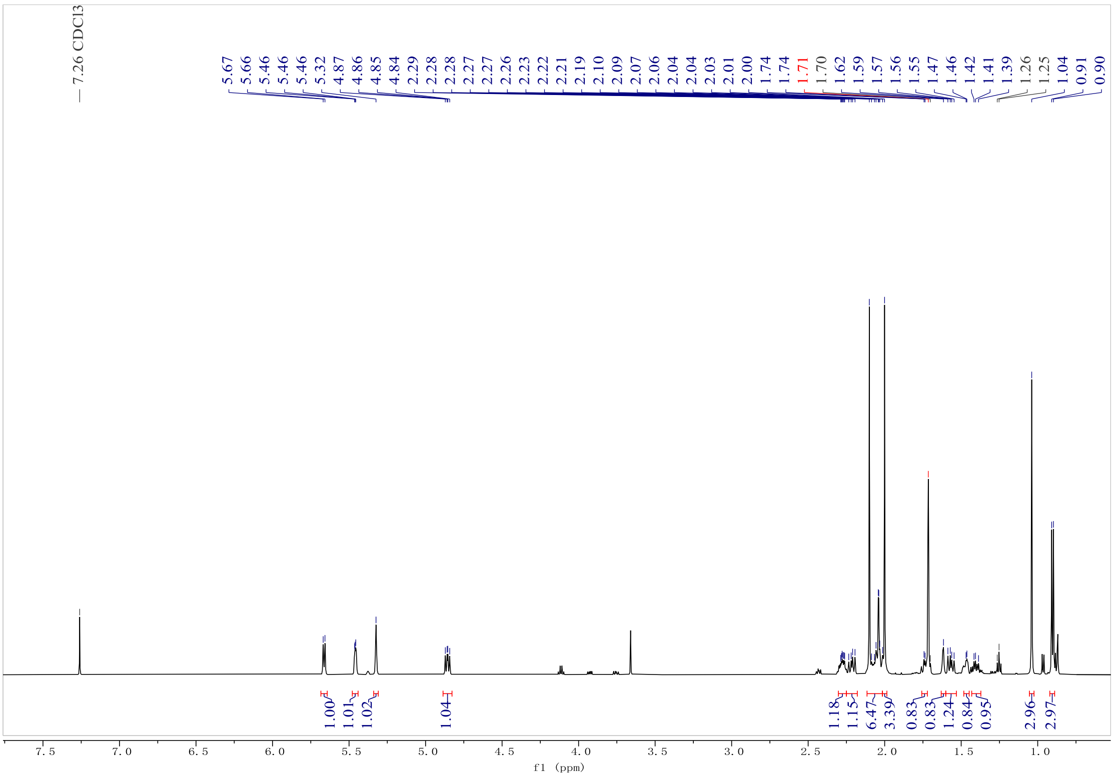

Figure S22. ^{1}H-NMR spectrum of lineolemnene G (**3**) in $CDCl_3$

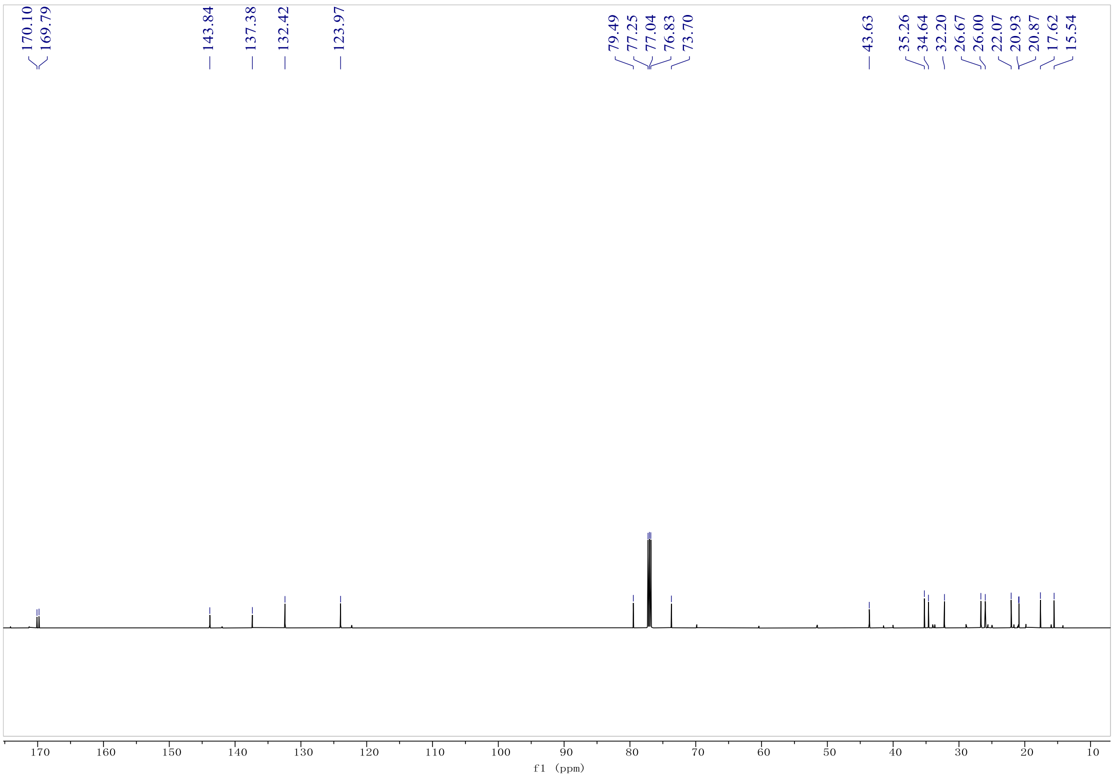

Figure S23. ^{13}C-NMR spectrum of lineolemnene G (**3**) in $CDCl_3$

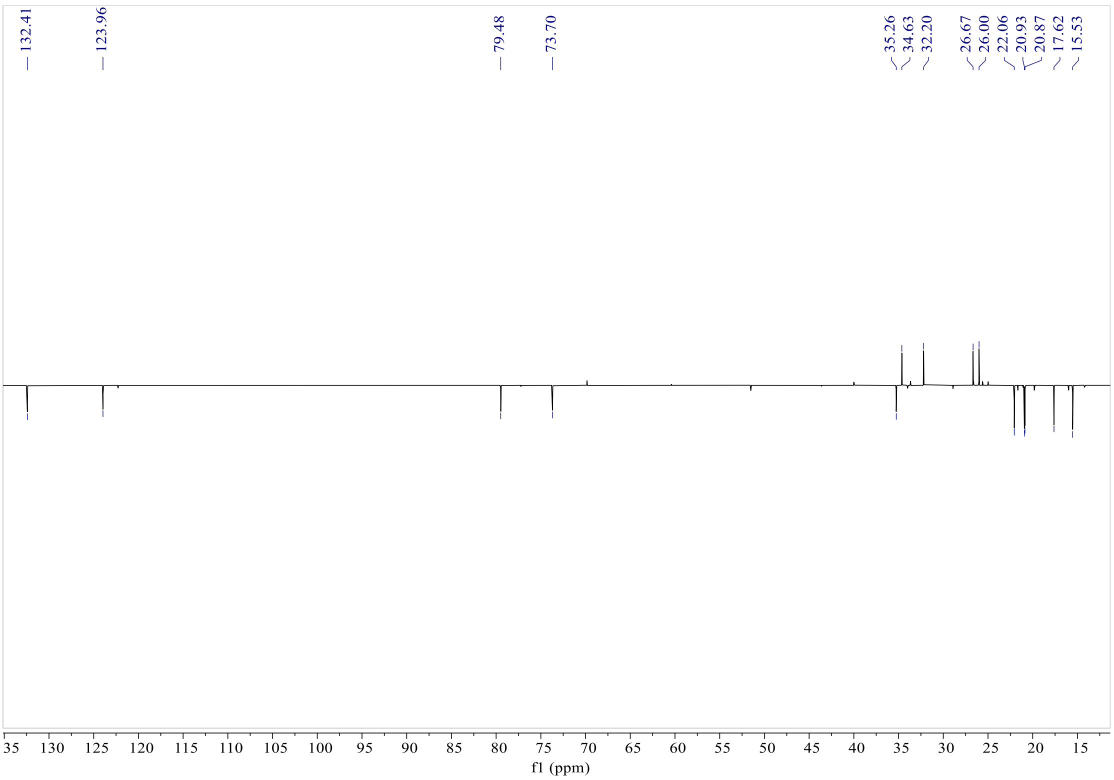

Figure S24. Dept 135° spectrum of lineolemnene G (**3**) in $CDCl_3$

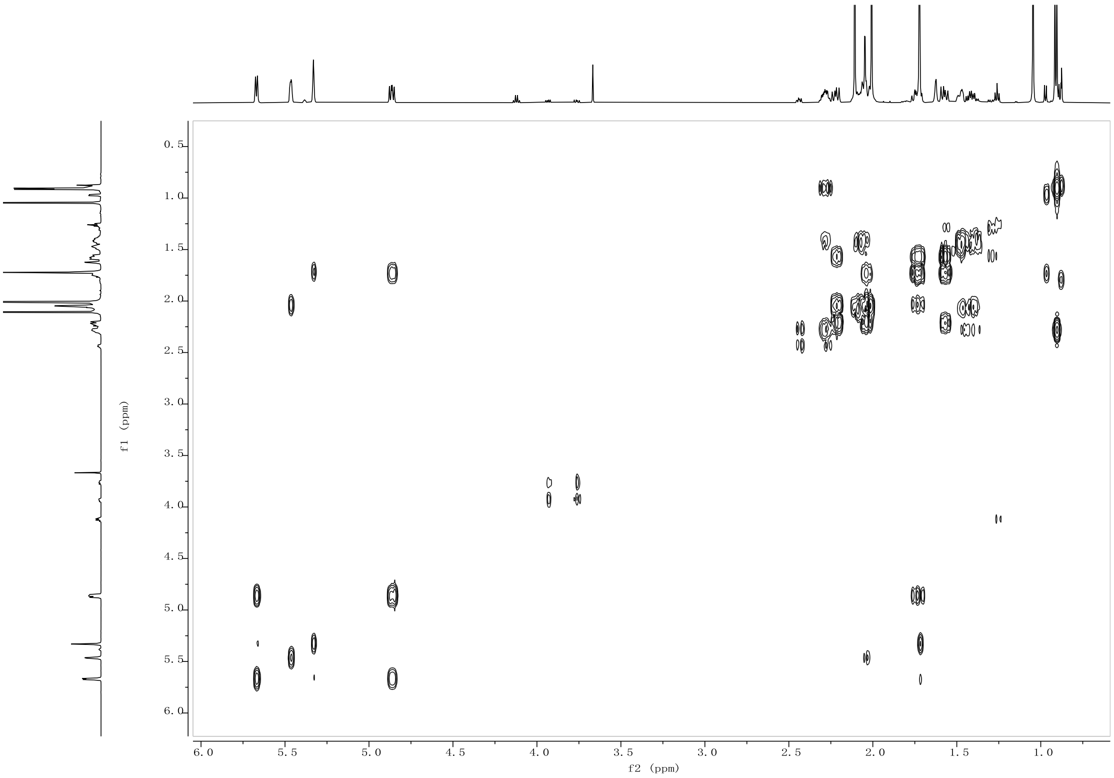

Figure S25. ^1H-^1H COSY spectrum of lineolemnene G (**3**) in $CDCl_3$

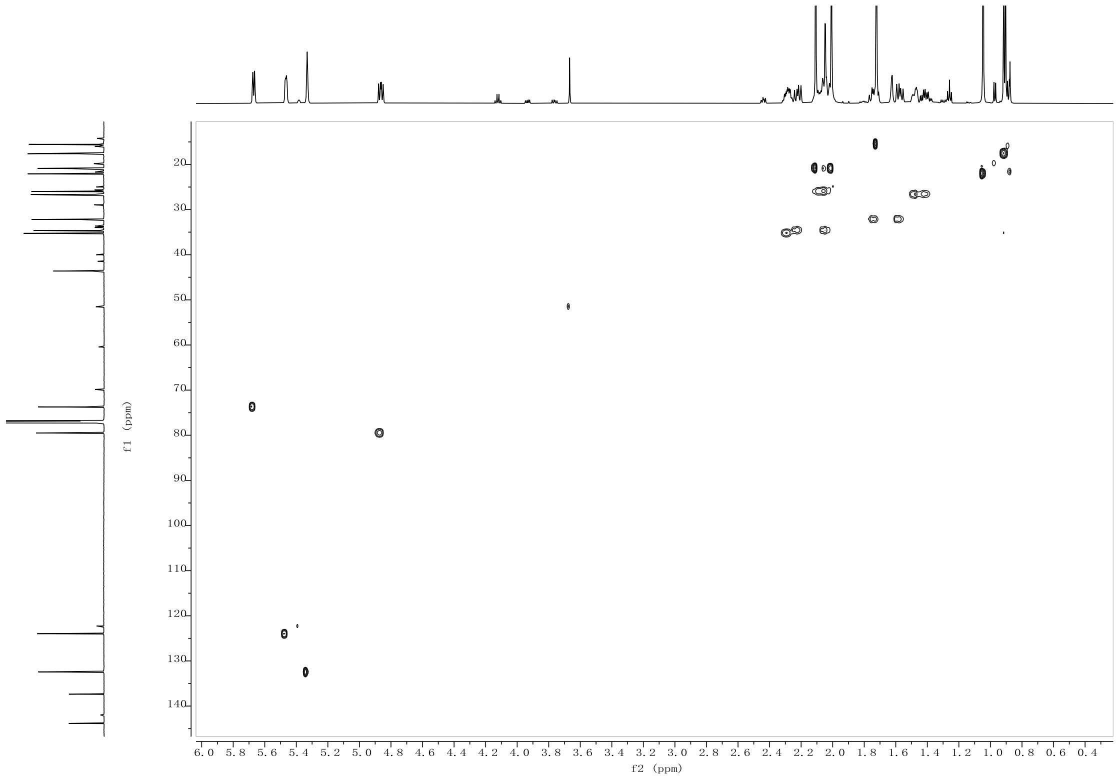

Figure S26. HSQC spectrum of lineolemnene G(**3**) in $CDCl_3$

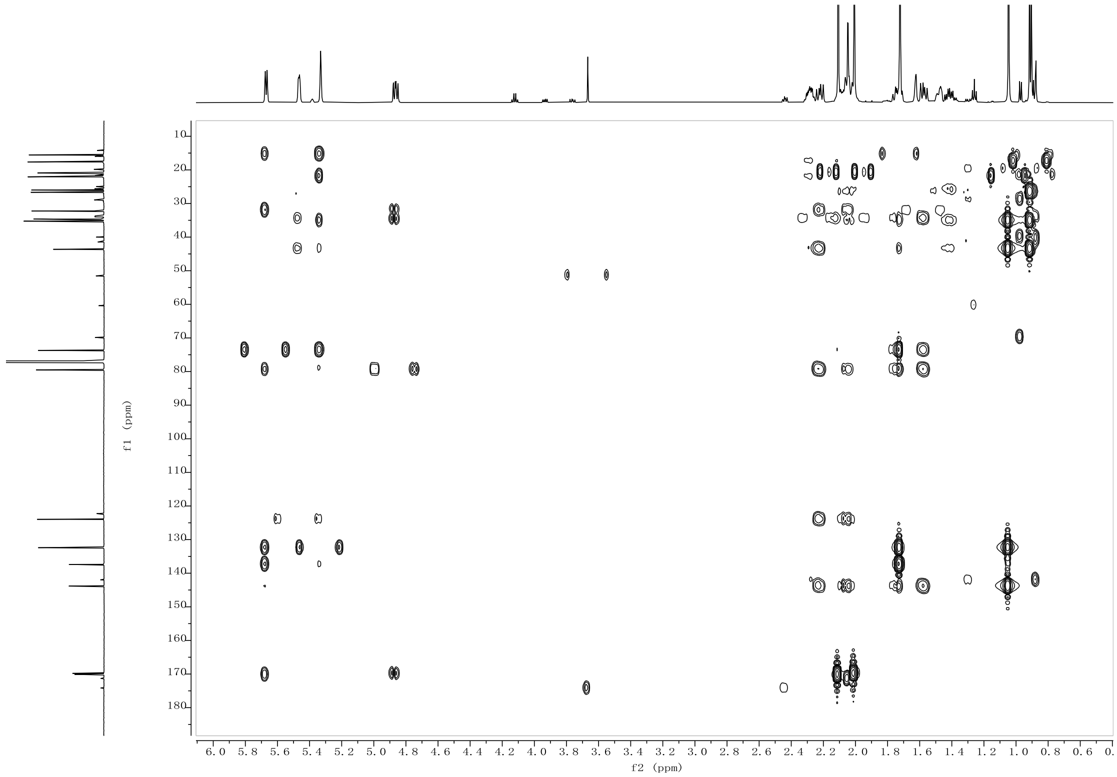

Figure S27. HMBC spectrum of lineolemnene G (**3**) in $CDCl_3$

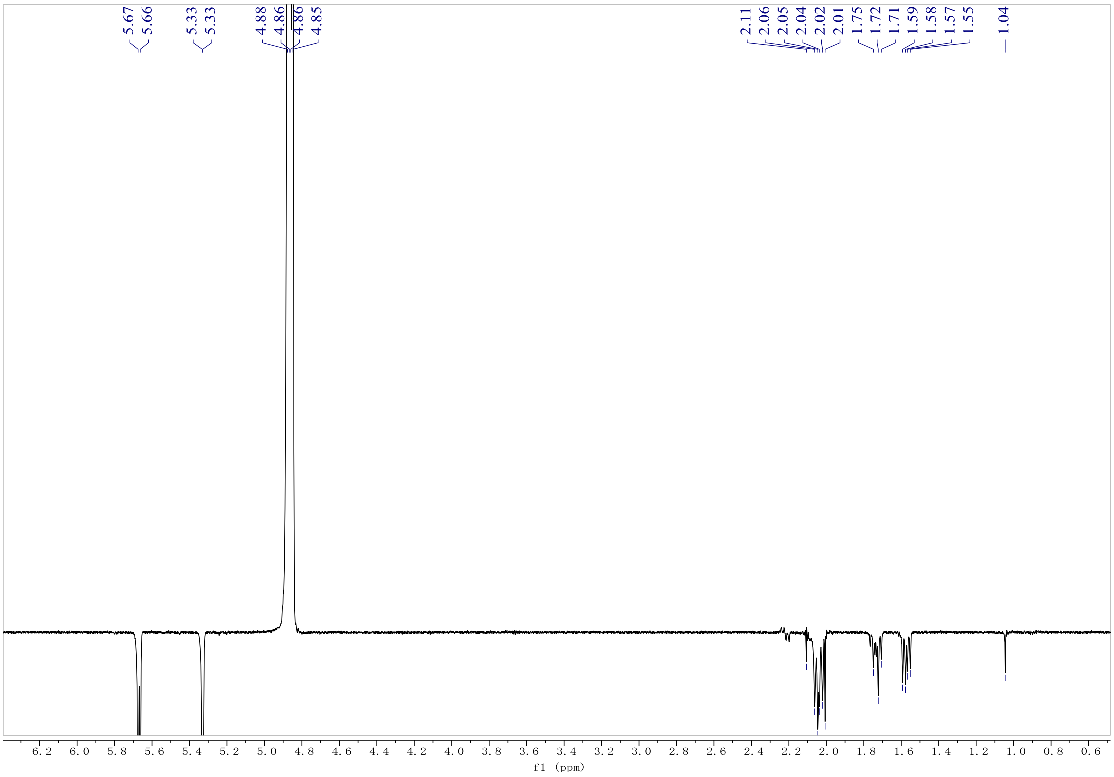
5.67
5.66
5.33
5.33
4.88
4.86
4.86
4.85
2.11
2.06
2.05
2.04
2.02
2.01
1.75
1.72
1.71
1.59
1.58
1.57
1.55
1.04
f1 (ppm)

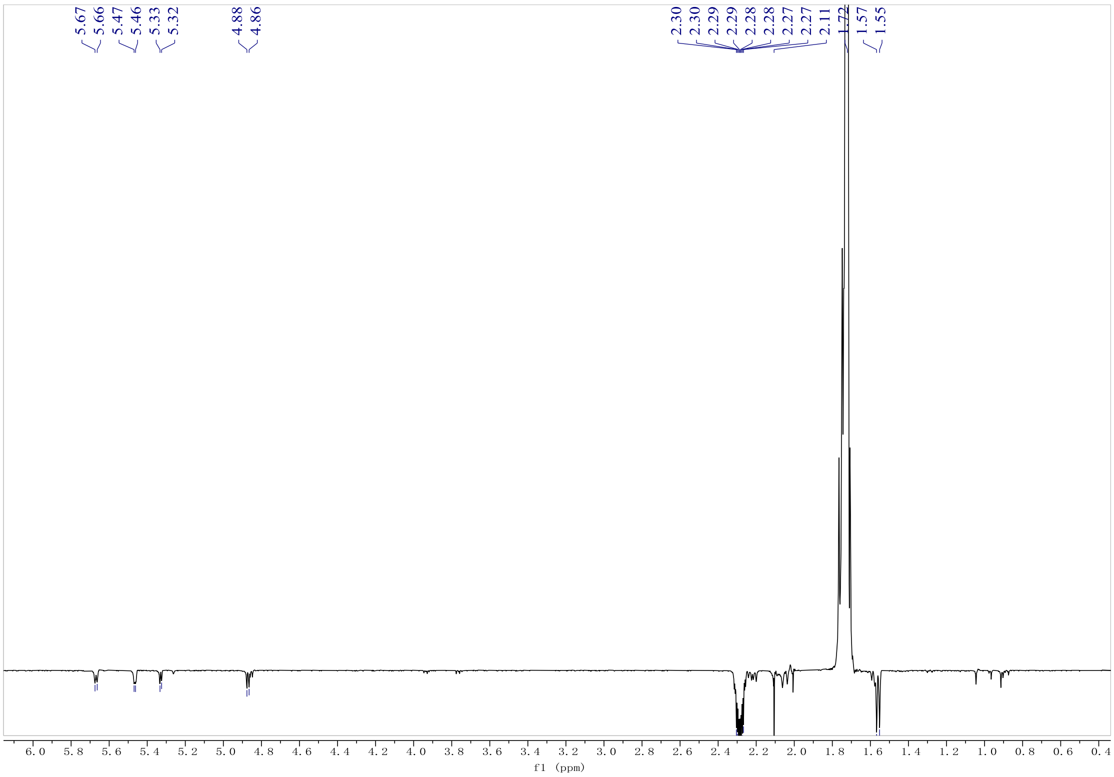
5.67
5.66
5.47
5.46
5.33
5.32
4.88
4.86
2.30
2.30
2.29
2.29
2.28
2.28
2.27
2.27
2.11
1.72
1.57
1.55
f1 (ppm)

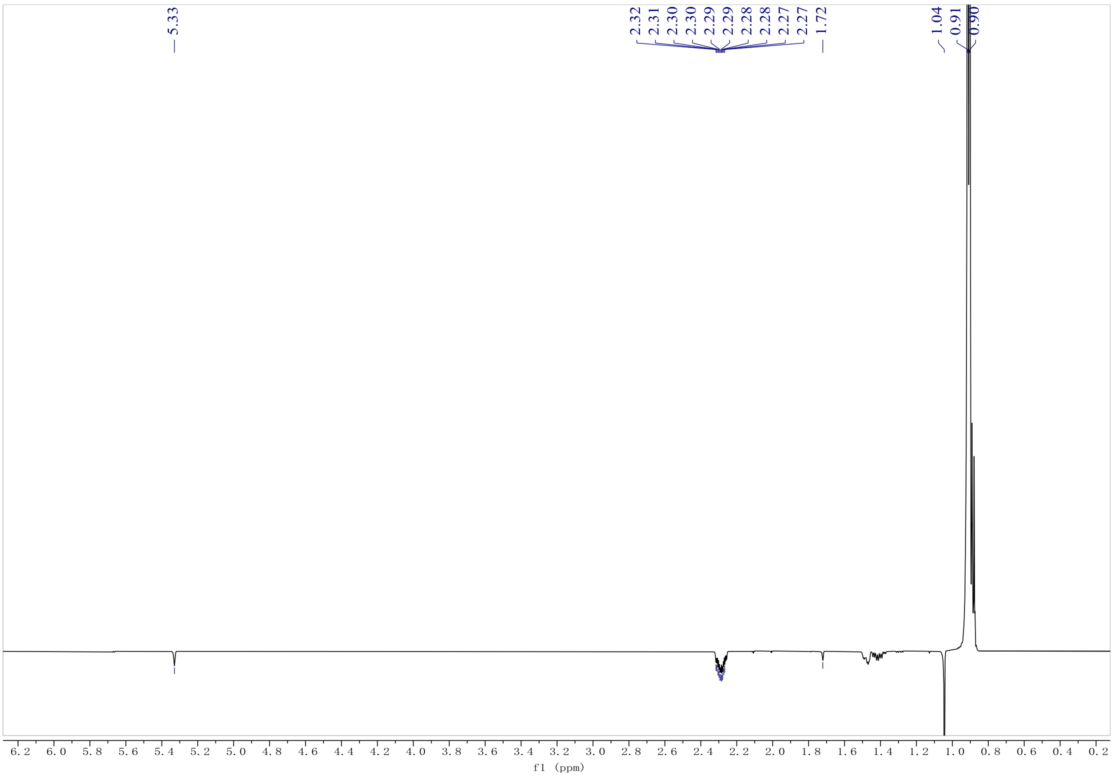

Figure S28. NOESY spectrum of lineolemnene G (**3**) in $CDCl_3$

8. Spectroscopic data for 2-acetoxy-aristolane (4)

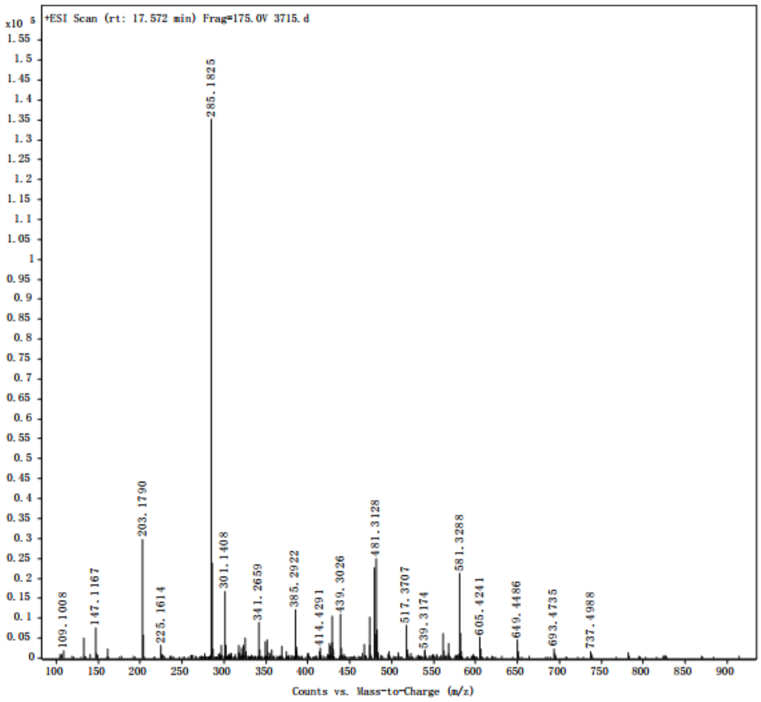

Figure S29. HRESIMS spectrum of 2-acetoxy-aristolane (**4**)

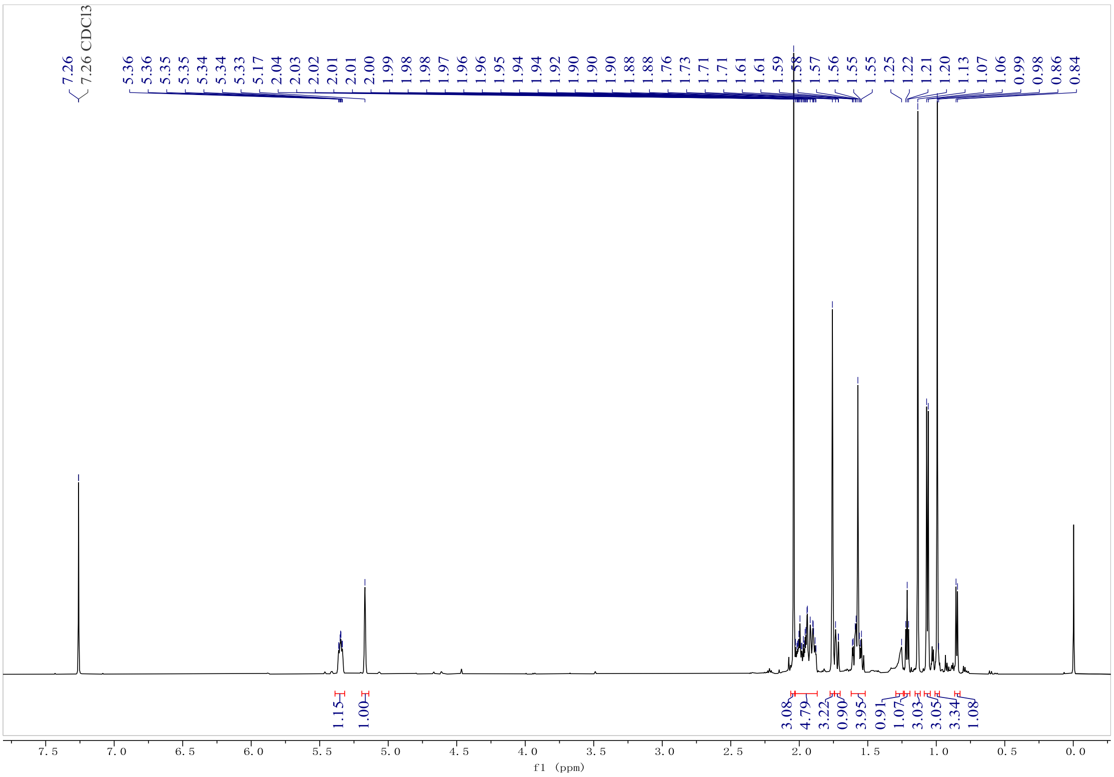

Figure S30. ^{1}H-NMR spectrum of 2-acetoxy-aristolane (**4**) in $CDCl_3$

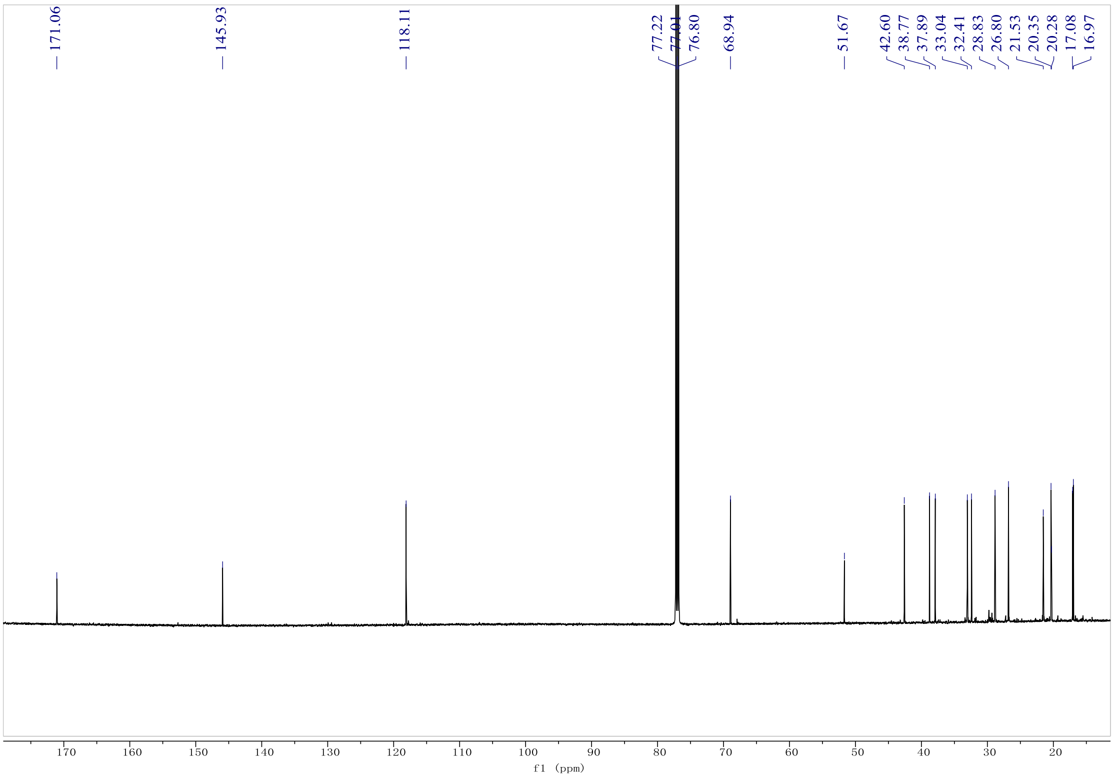

Figure S31. ^{13}C-NMR spectrum of 2-acetoxy-aristolane (**4**) in $CDCl_3$

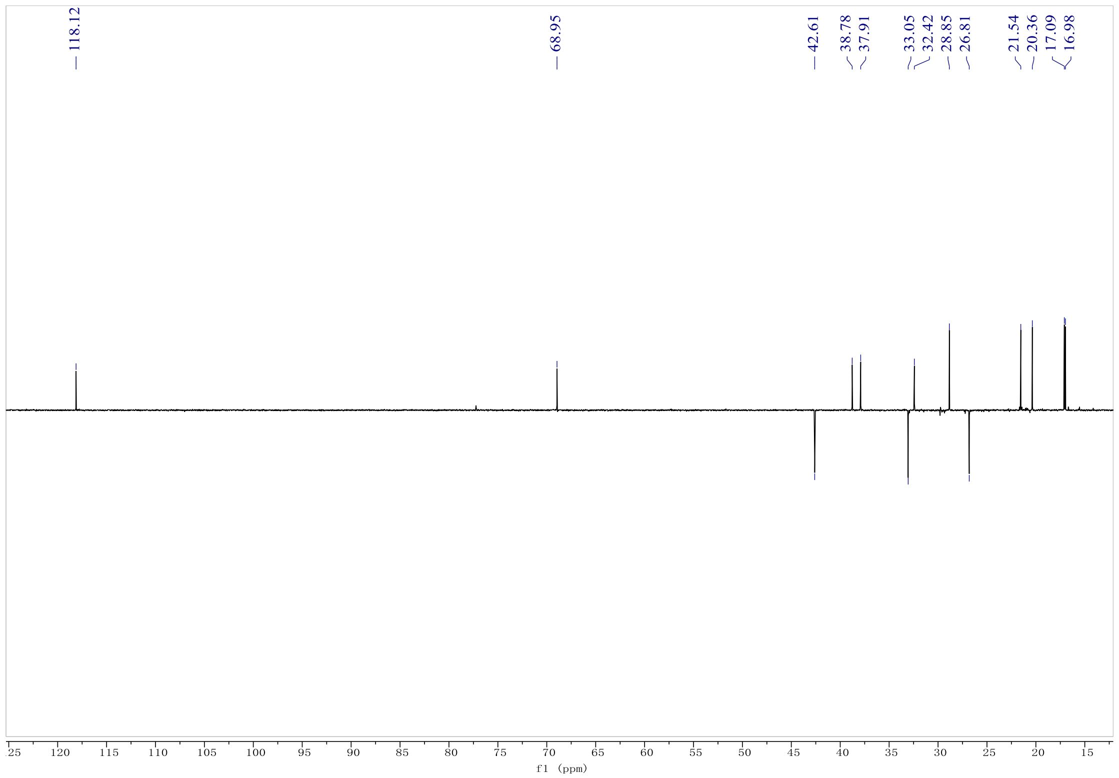

Figure S32. Dept 135° spectrum of 2-acetoxy-aristolane (**4**) in $CDCl_3$

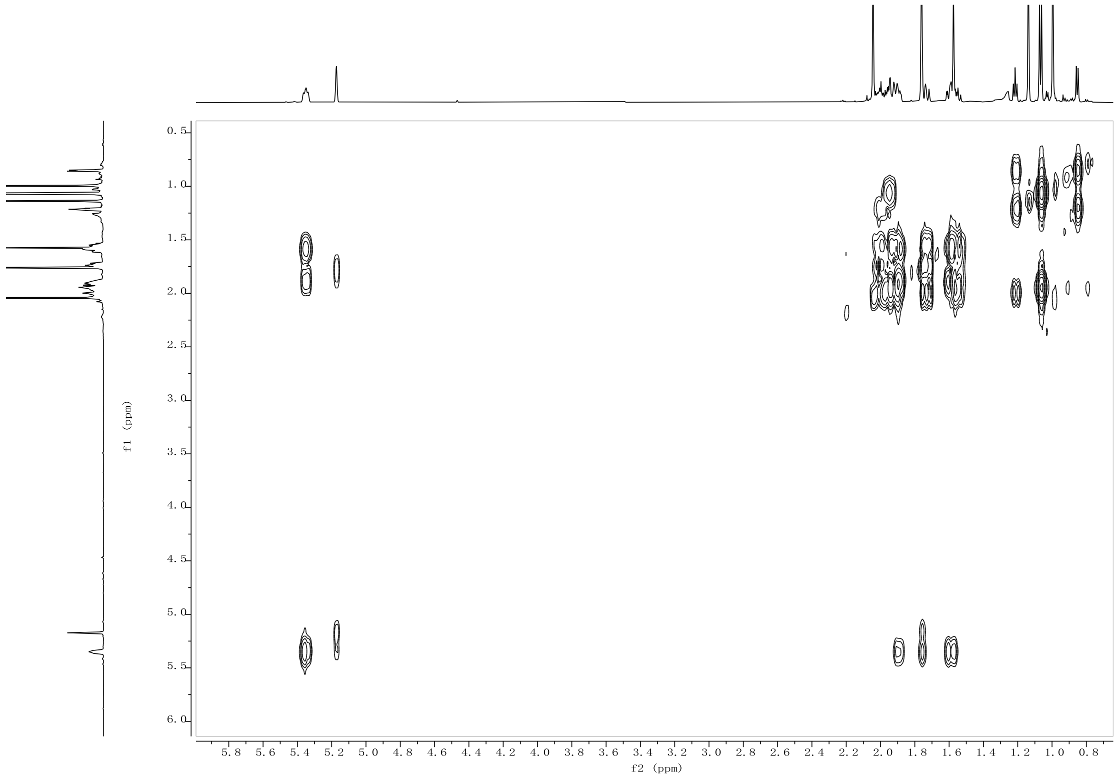

Figure S33. ^1H-^1H COSY spectrum of 2-acetoxy-aristolane (**4**) in $CDCl_3$

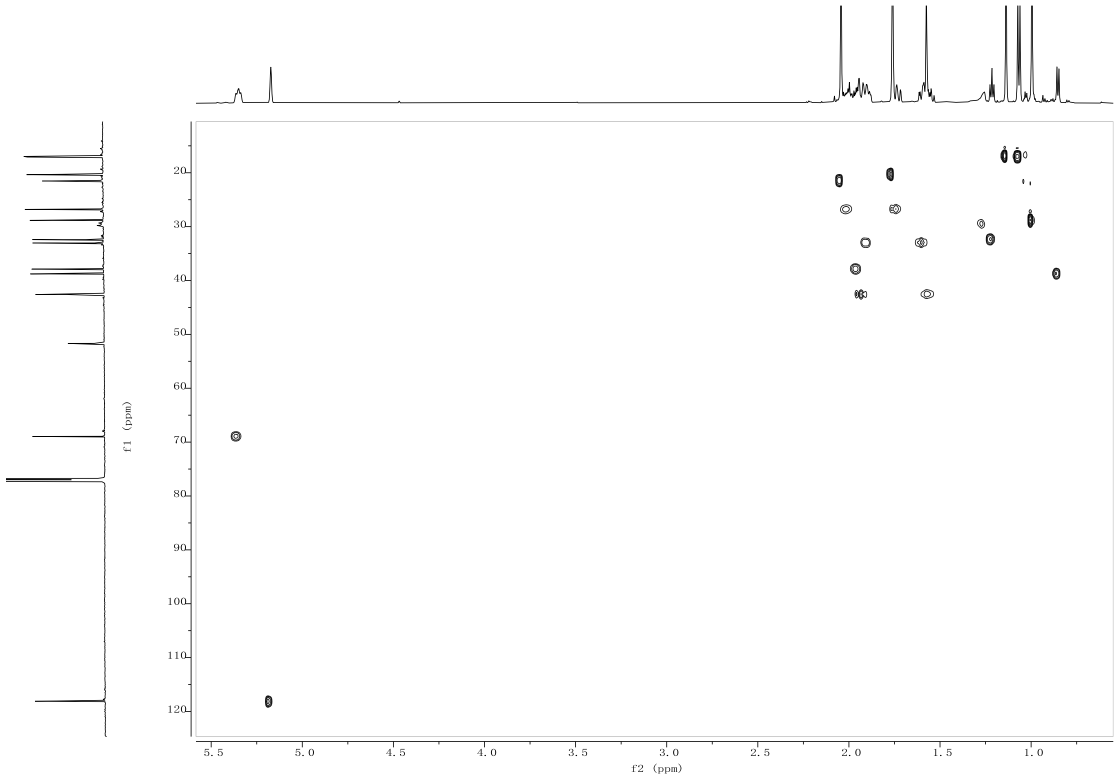

Figure S34. HSQC spectrum of 2-acetoxy-aristolane (**4**) in $CDCl_3$

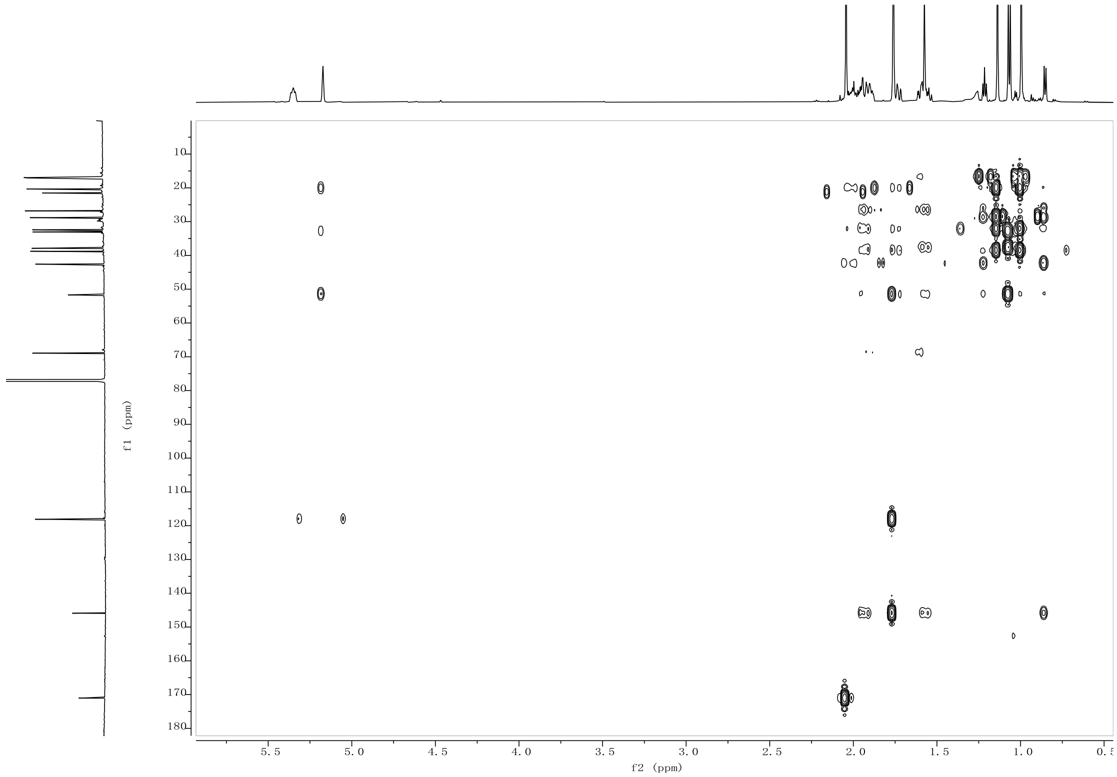

Figure S35. HMBC spectrum of 2-acetoxy-aristolane (**4**) in $CDCl_3$

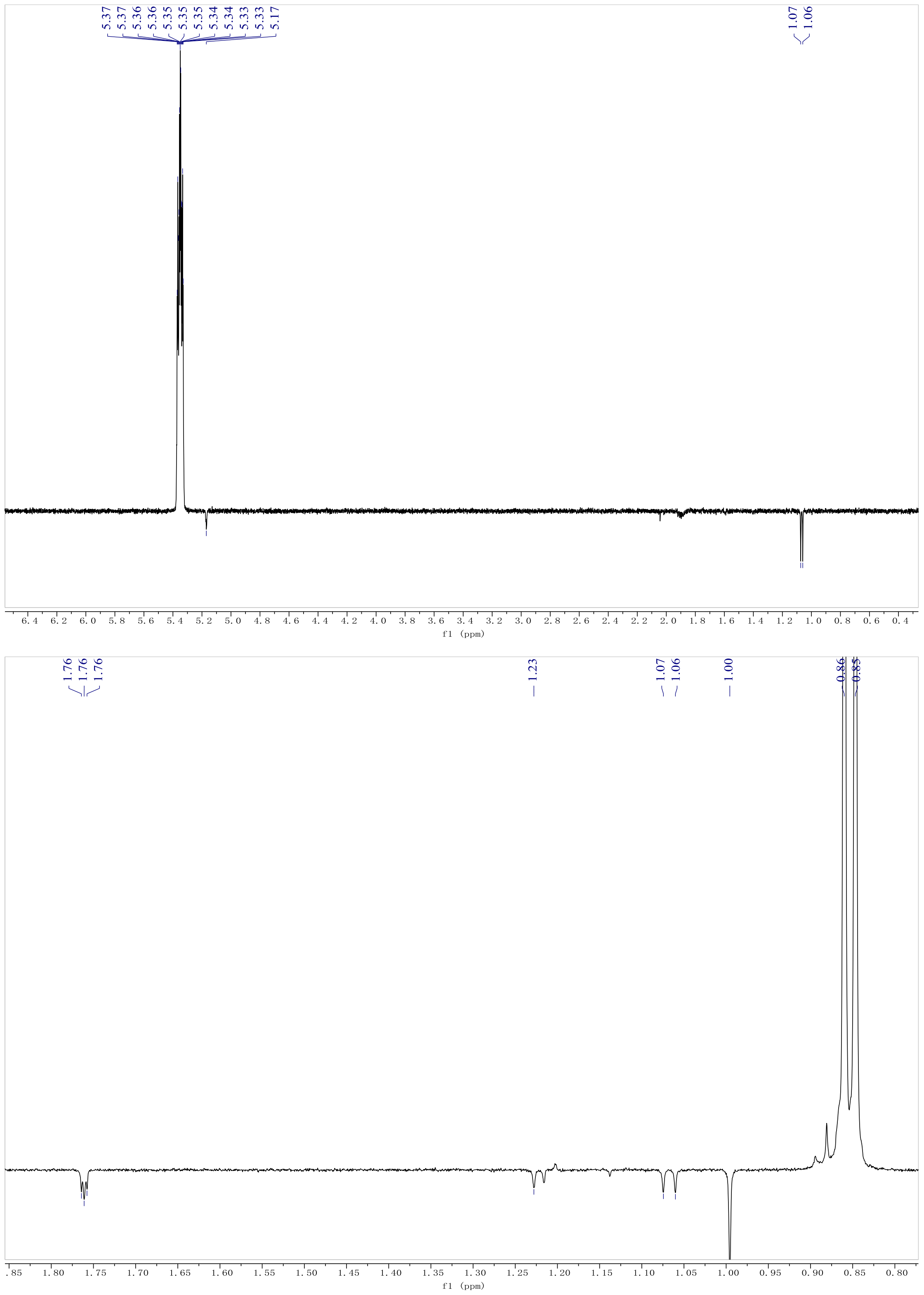

Figure S36. NOESY spectrum of 2-acetoxy-aristolane (**4**) in $CDCl_3$

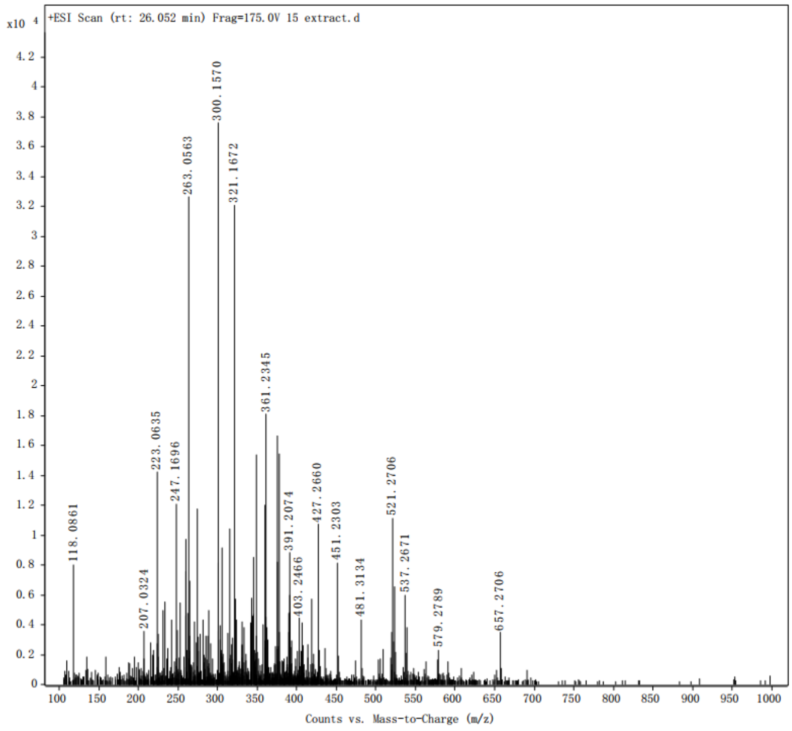

Figure S37. [M + H]+ of **4** extracted from the TIC of *Lemnalia* sp. methanol extract

9. Spectroscopic data for biofloranate A (5)

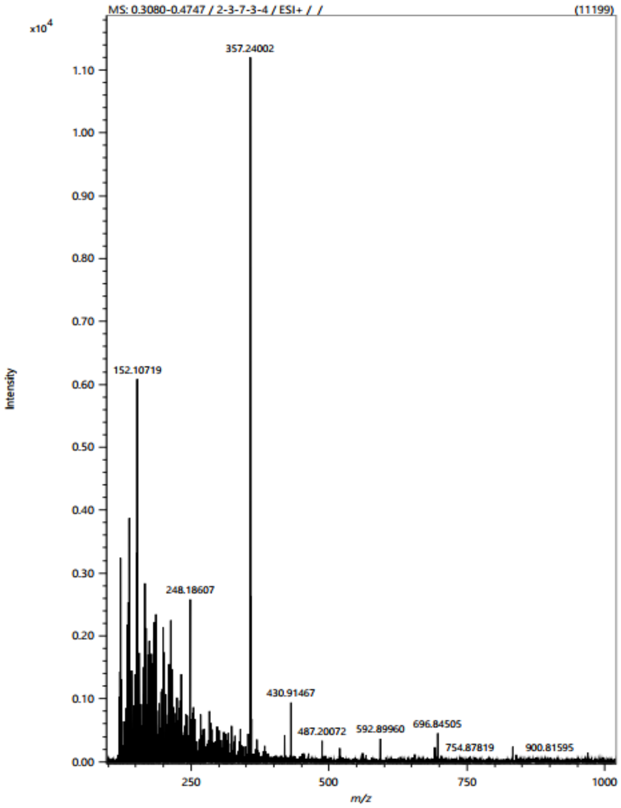

Figure S38. HRESIMS spectrum of biofloranate A (**5**)

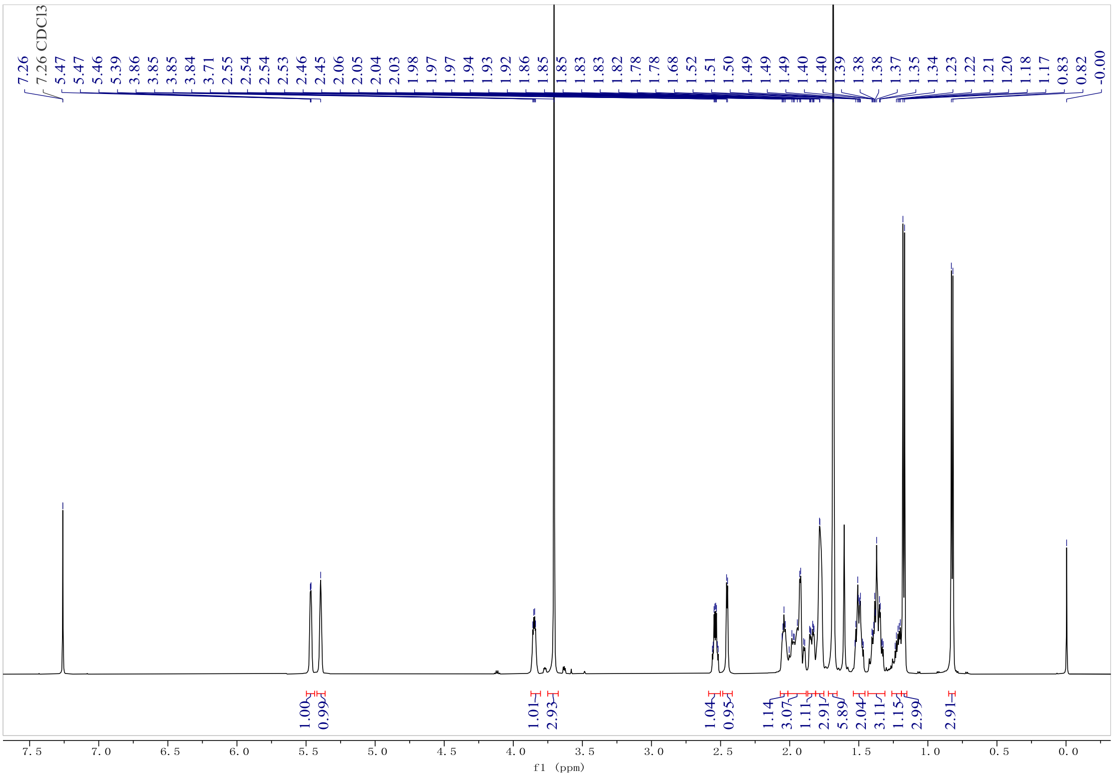

Figure S39. ^{1}H-NMR spectrum of biofloranate A (**5**) in $CDCl_3$

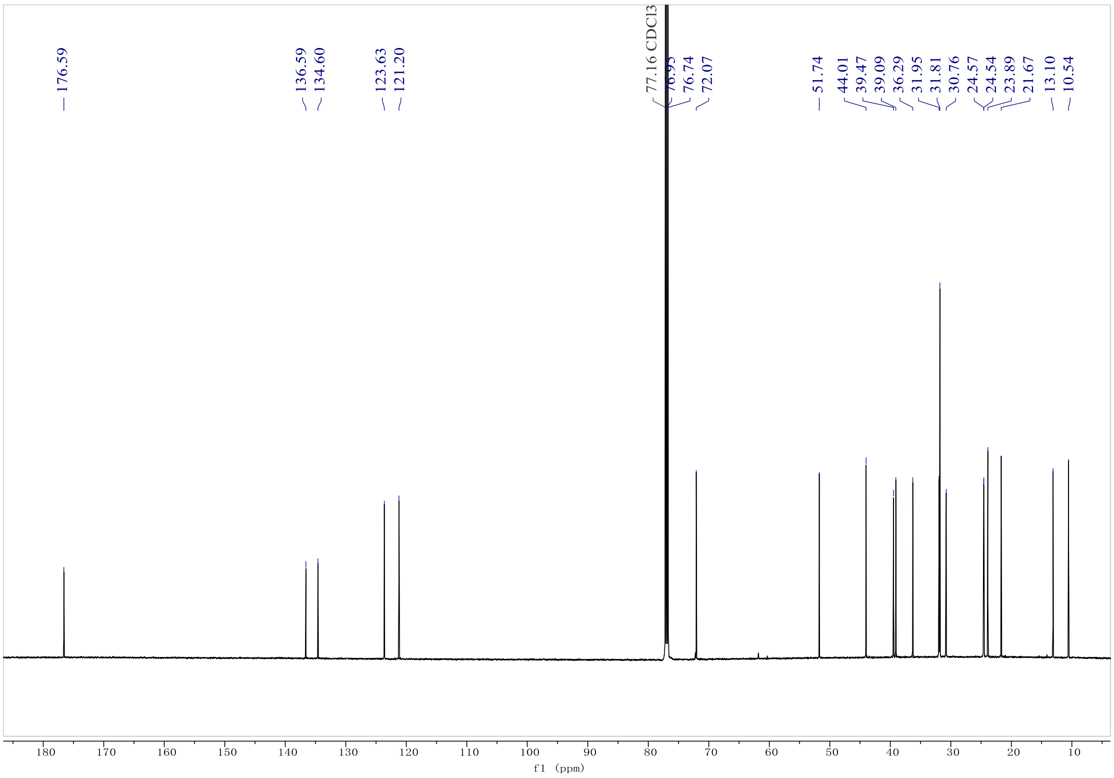

Figure S40. ^{13}C-NMR spectrum of biofloranate A (**5**) in $CDCl_3$

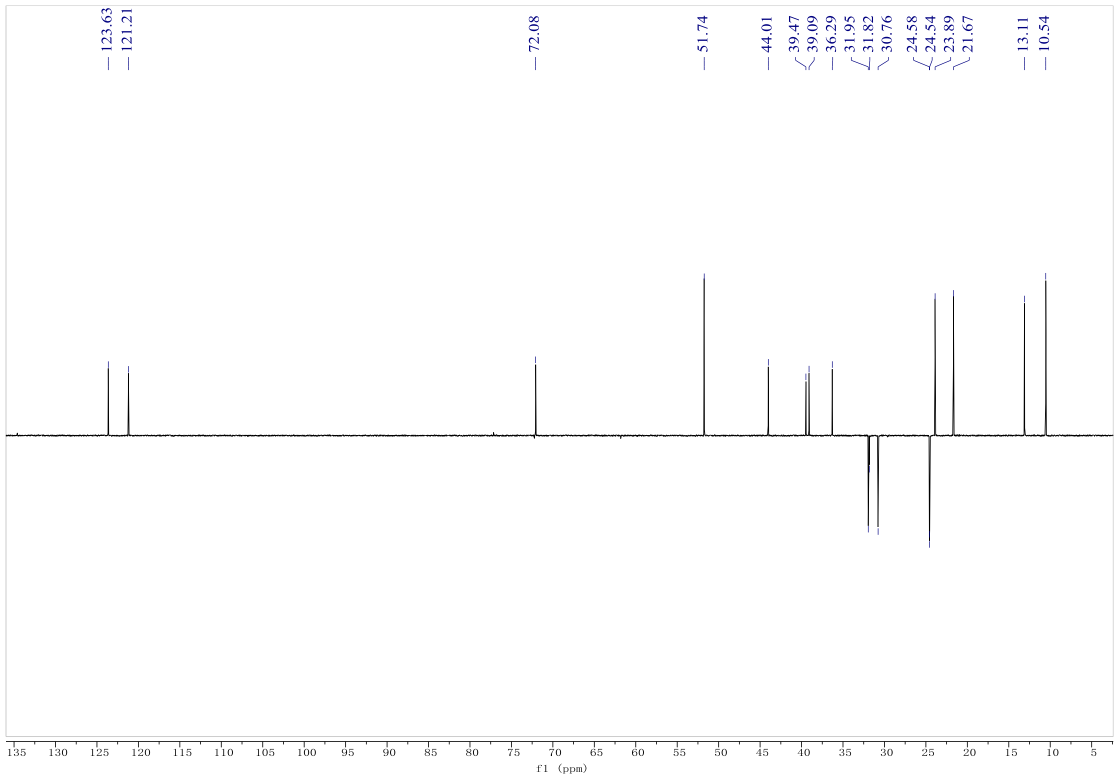

Figure S41. Dept 135° spectrum of biofloranate A (**5**) in $CDCl_3$

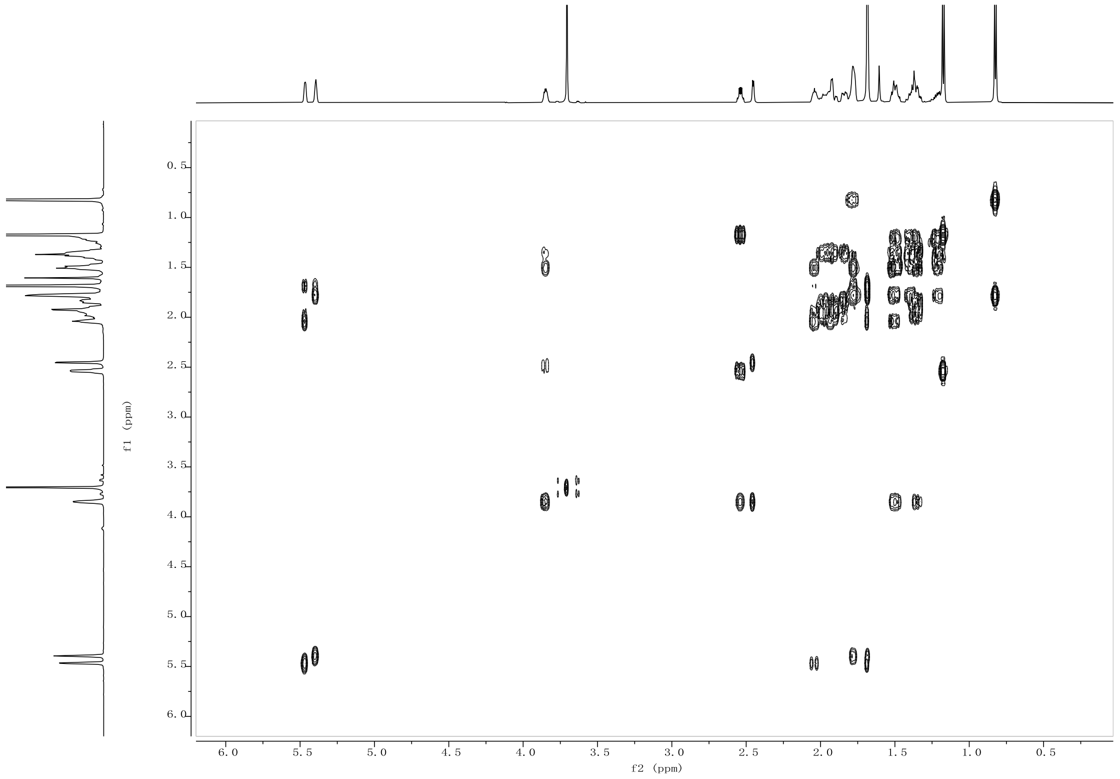

Figure S42. ^{1}H-^{1}H COSY spectrum of biofloranate A (**5**) in $CDCl_3$

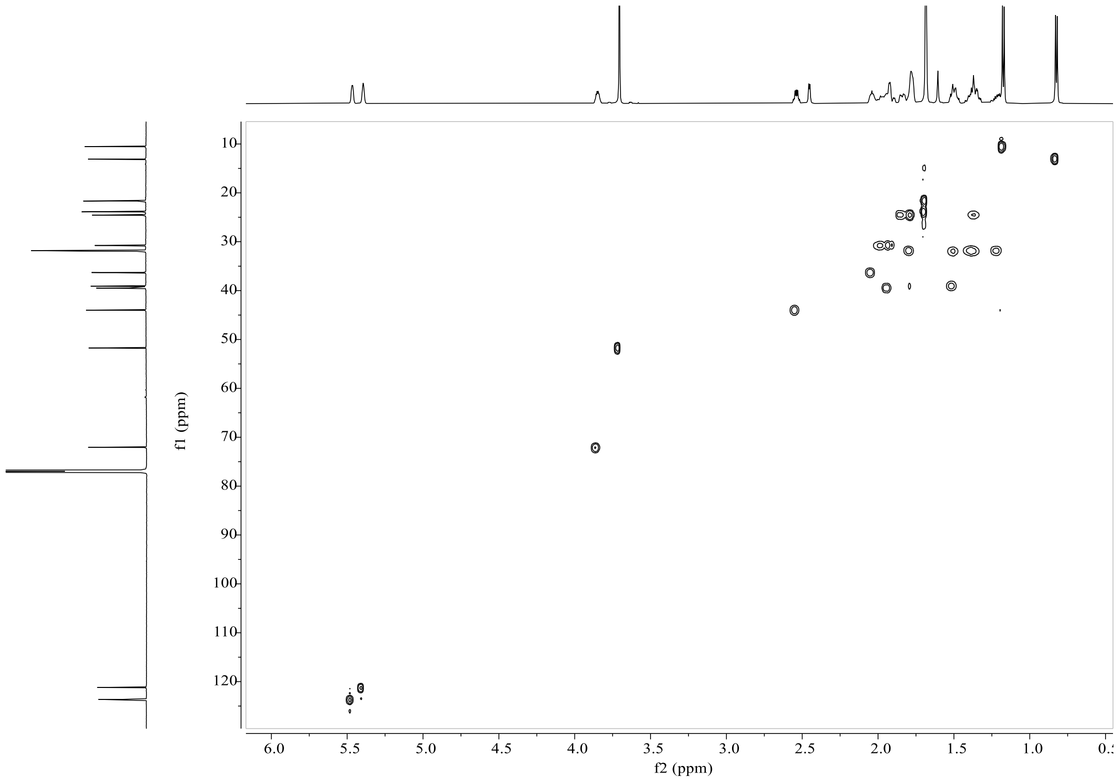

Figure S43. HSQC spectrum of biofloranate A (**5**) in $CDCl_3$

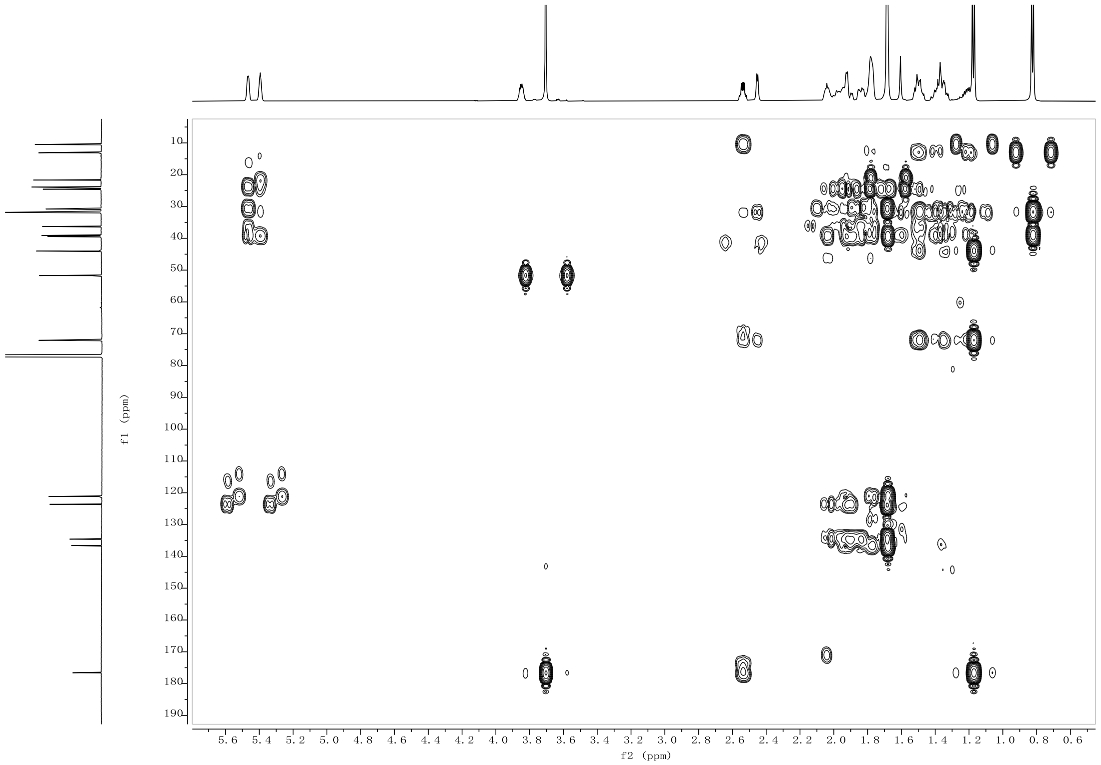

Figure S44. HMBC spectrum of biofloranate A (**5**) in $CDCl_3$

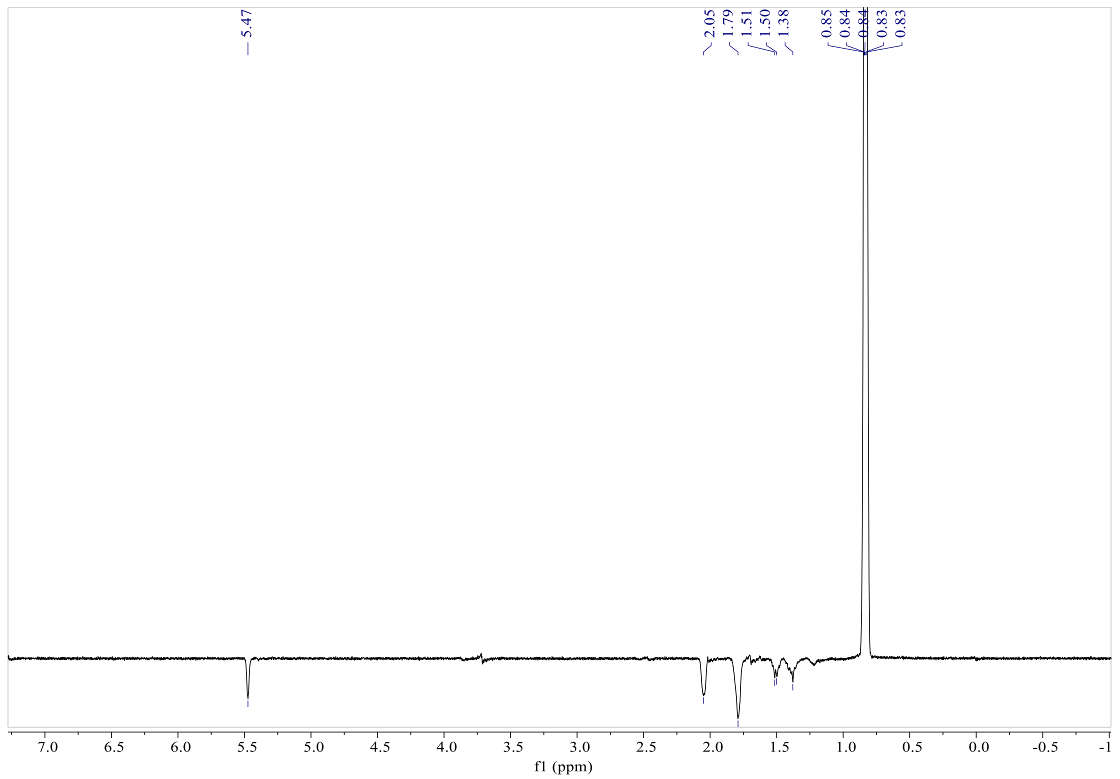

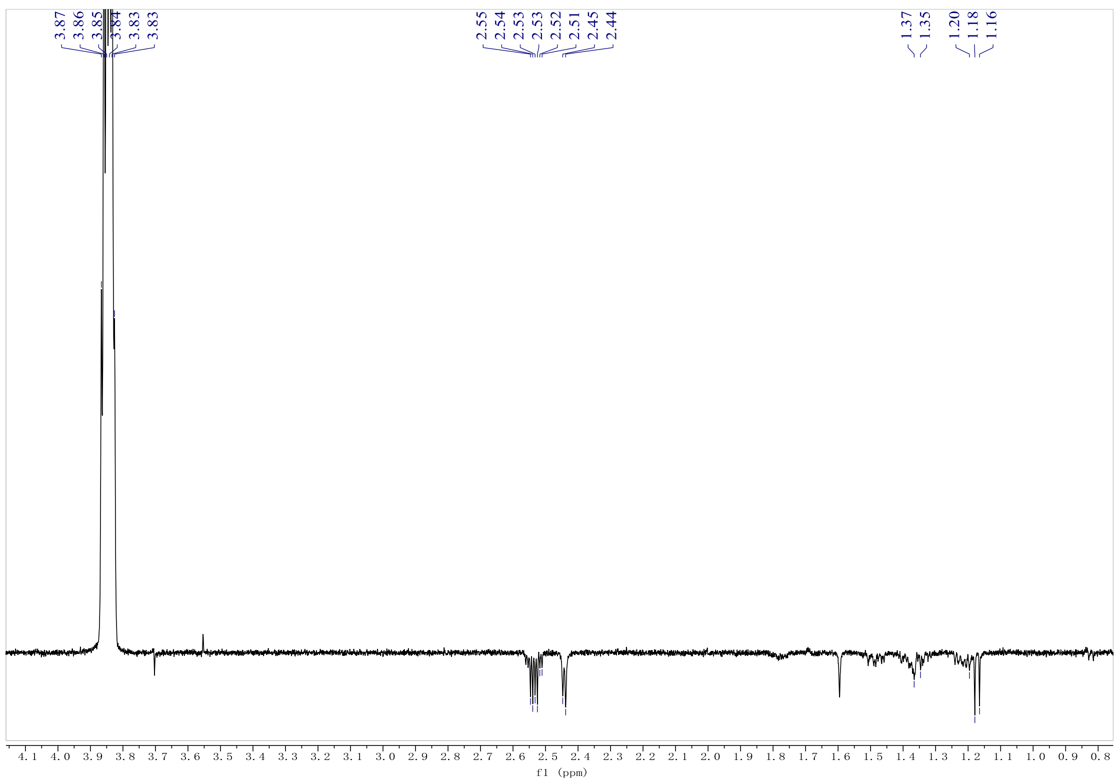

Figure S45. NOESY spectrum of biofloranate A (**5**) in $CDCl_3$

10. Spectroscopic data for biofloranate B (6)

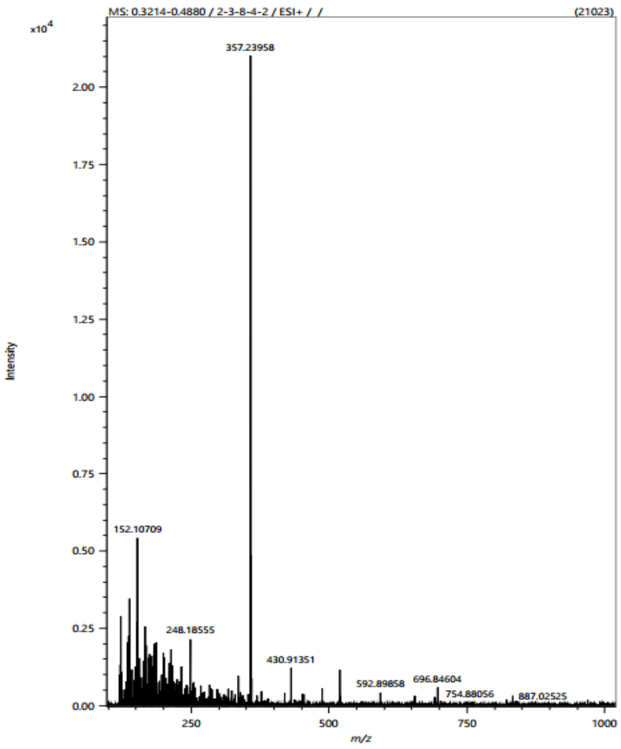

Figure S46. HRESIMS spectrum of biofloranate B (**6**)

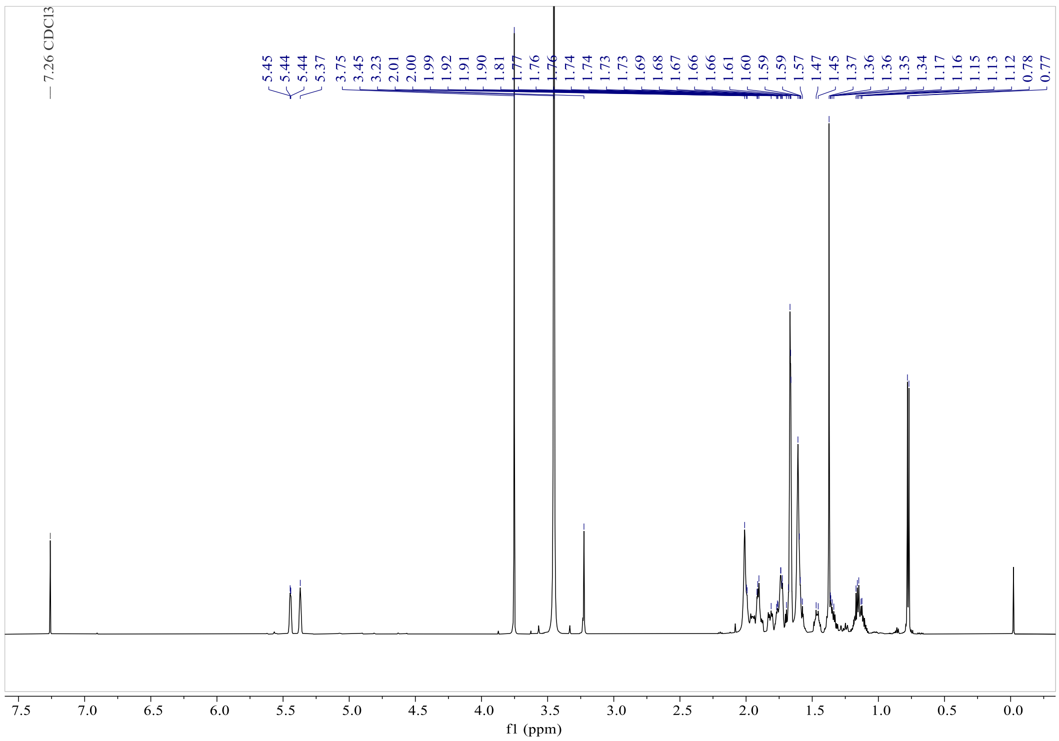

Figure S47. ^{1}H-NMR spectrum of biofloranate B (**6**) in $CDCl_3$

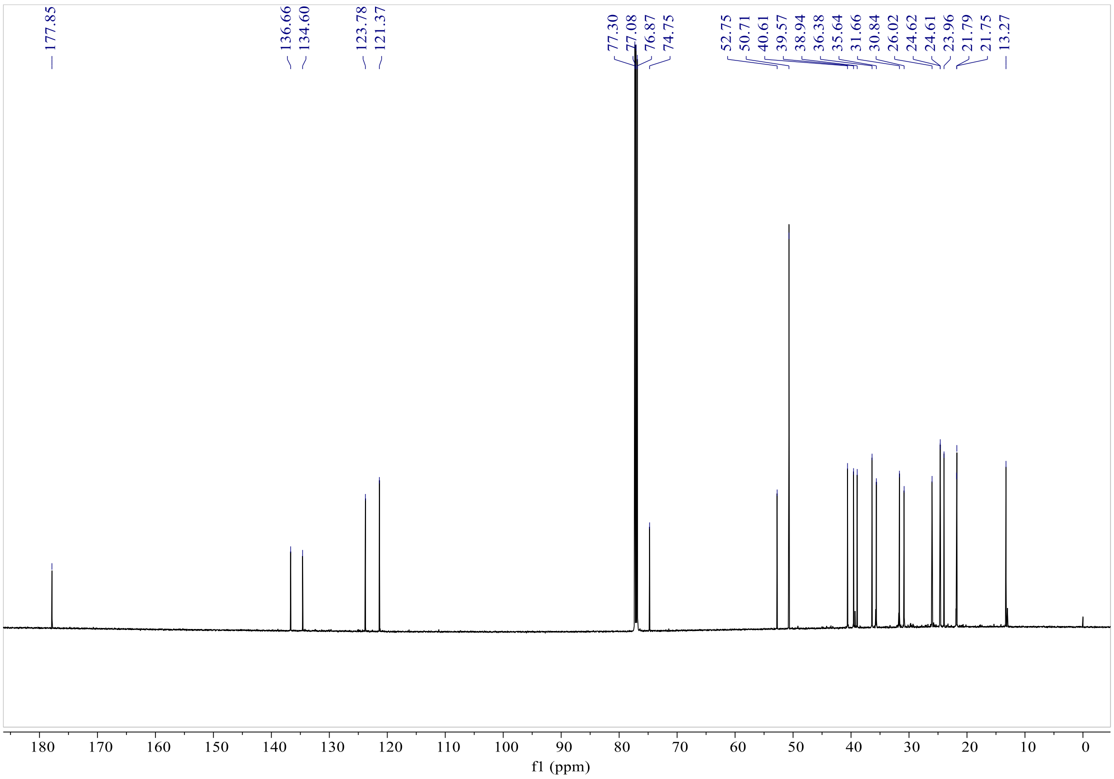

Figure S48. ^{13}C-NMR spectrum of biofloranate B (**6**) in $CDCl_3$

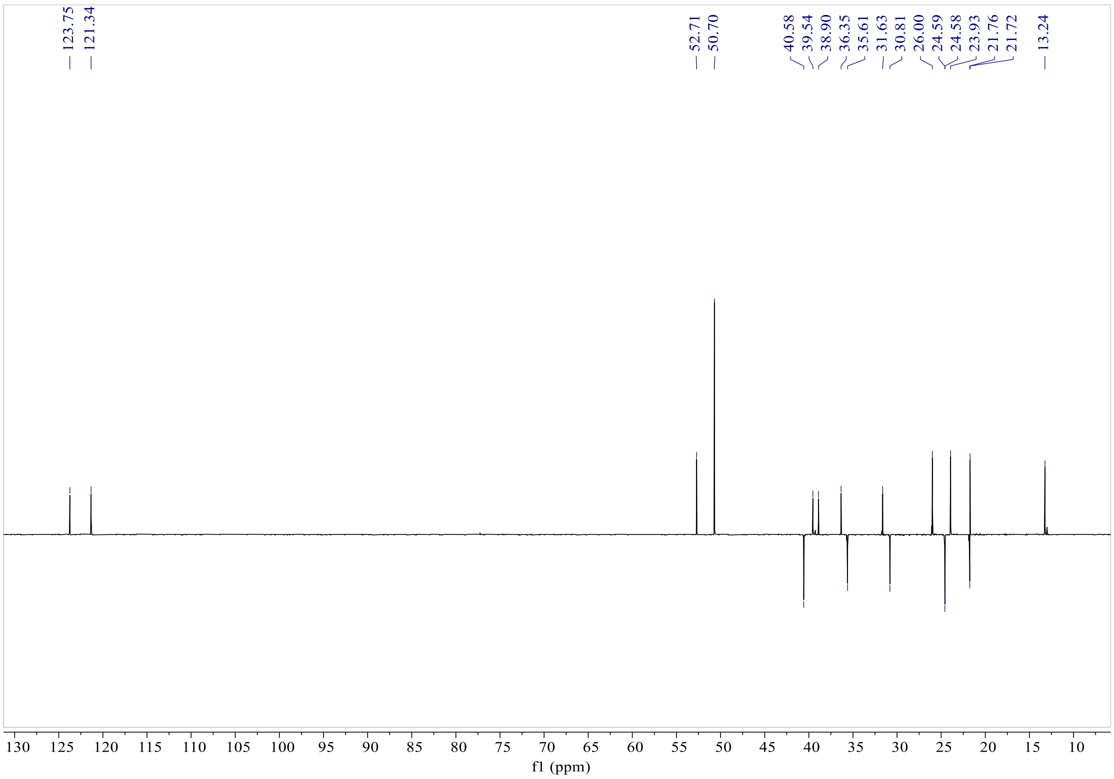

Figure S49. Dept 135° spectrum of biofloranate B (**6**) in $CDCl_3$

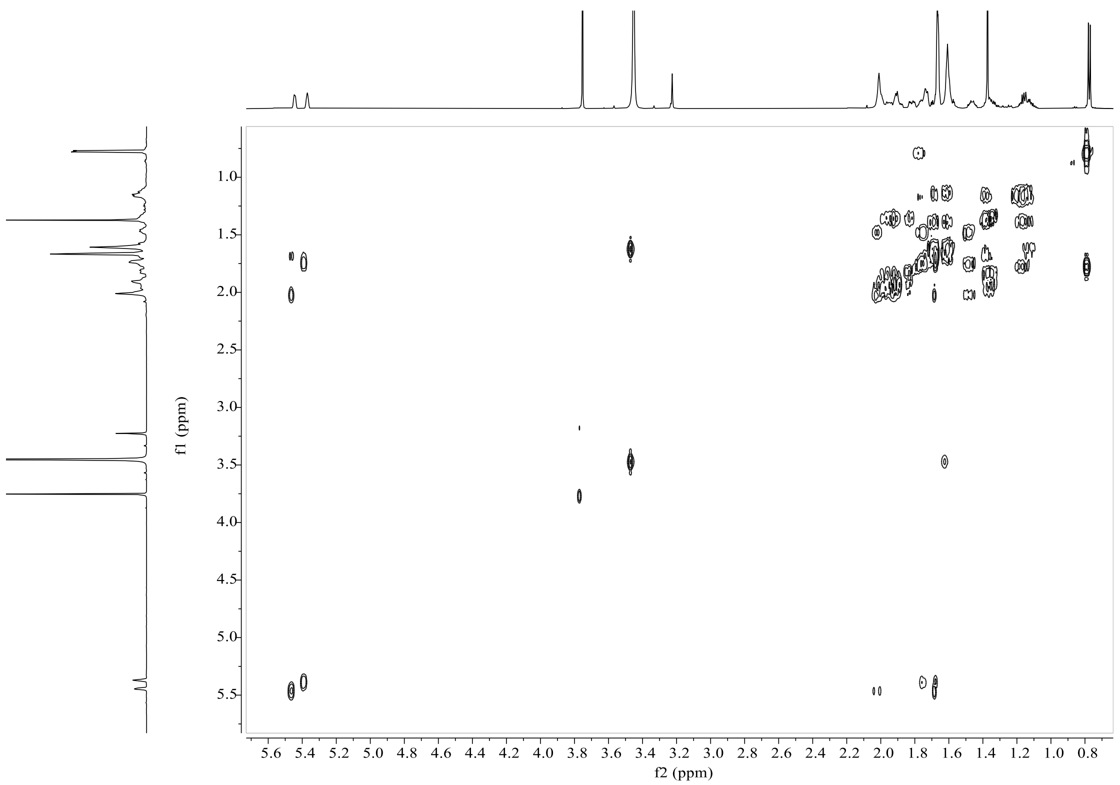

Figure S50. ^{1}H-^{1}H COSY spectrum of biofloranate B (**6**) in $CDCl_3$

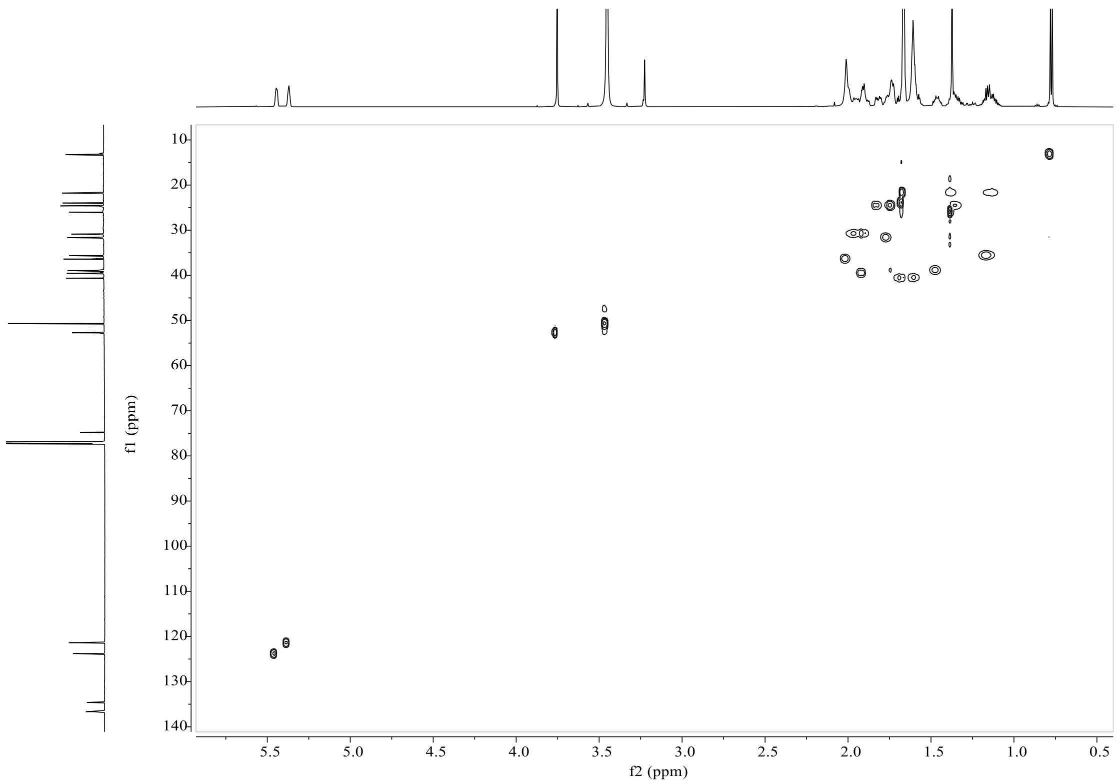

Figure S51. HSQC spectrum of biofloranate B (**6**) in $CDCl_3$

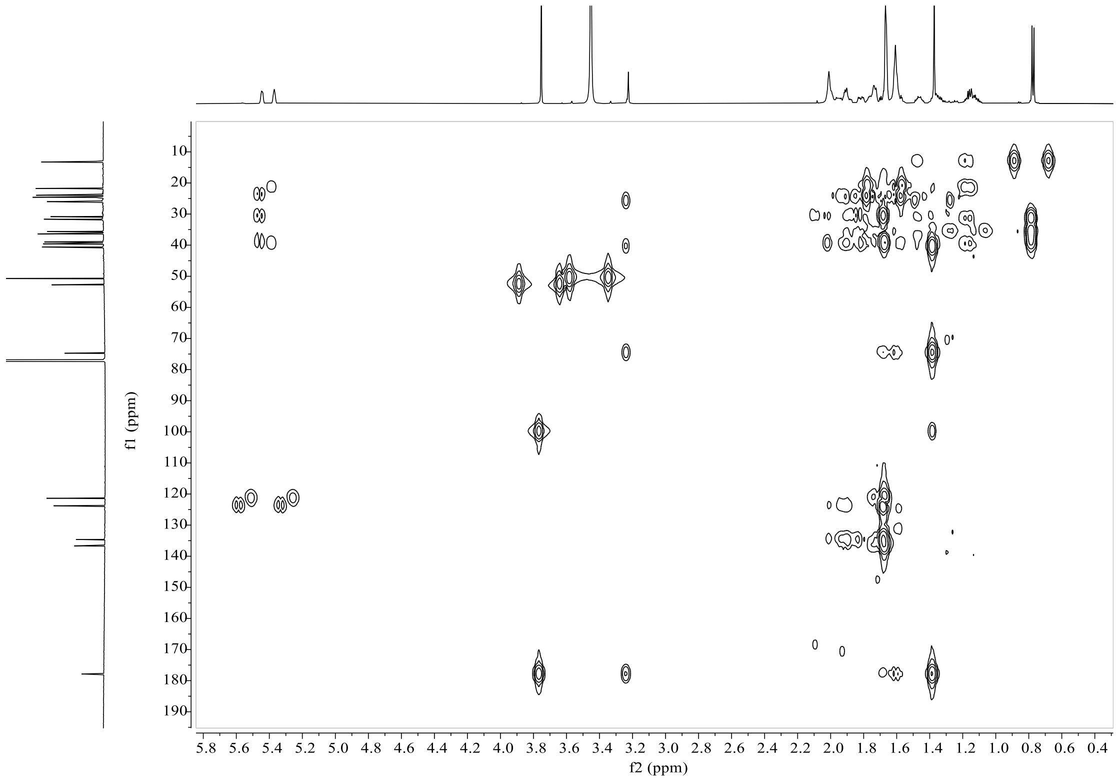

Figure S52. HMBC spectrum of biofloranate B (**6**) in $CDCl_3$

11. Spectroscopic data for biofloranate C (7)

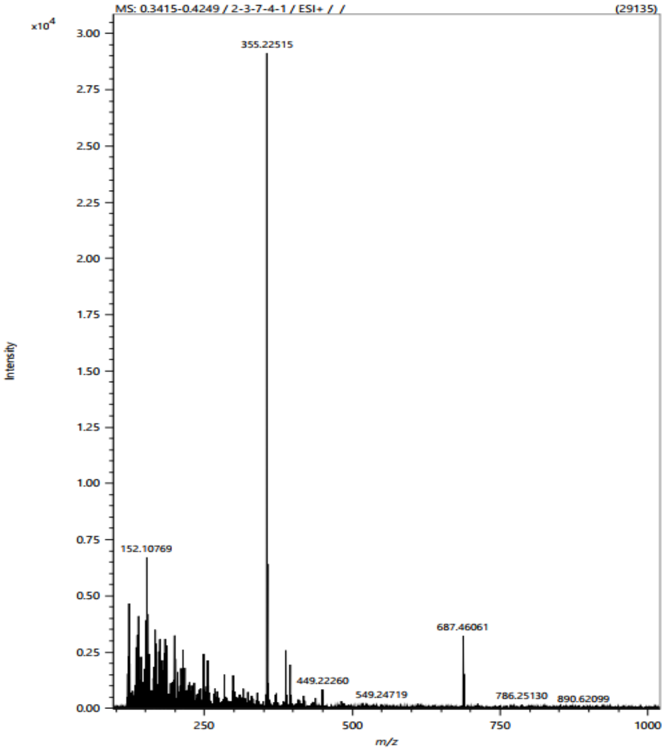

Figure S53. HRESIMS spectrum of biofloranate C (**7**)

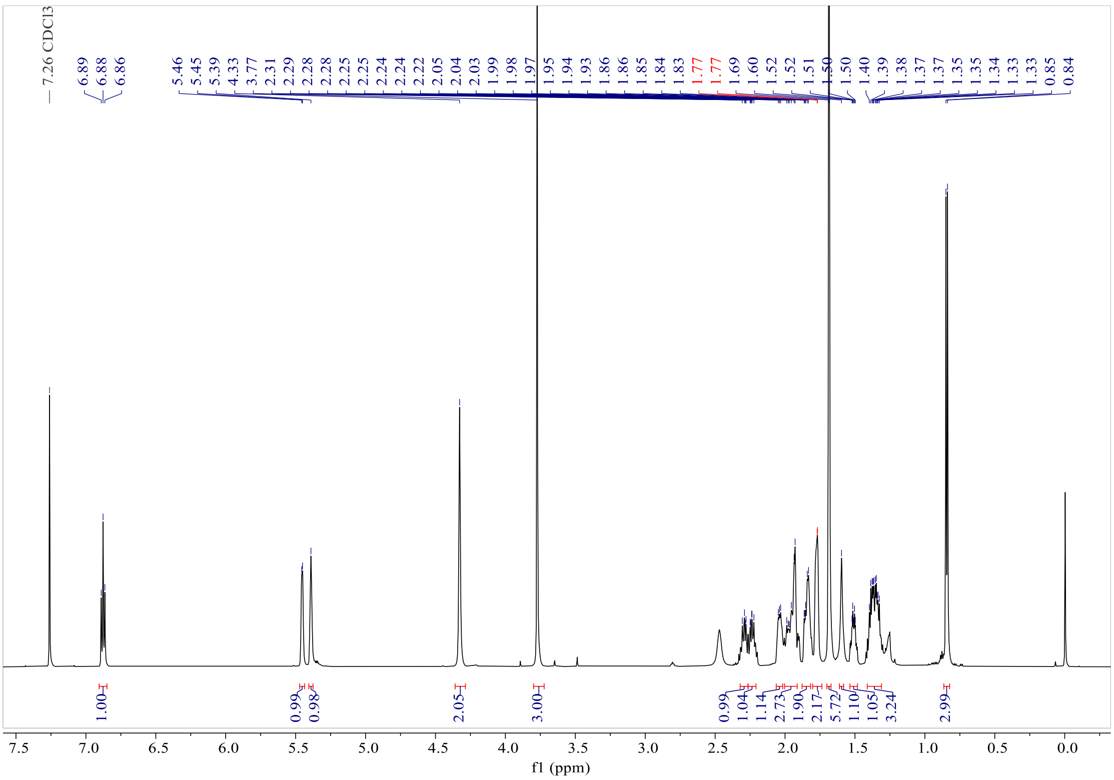

Figure S54. ^{1}H-NMR spectrum of biofloranate C (**7**) in $CDCl_3$

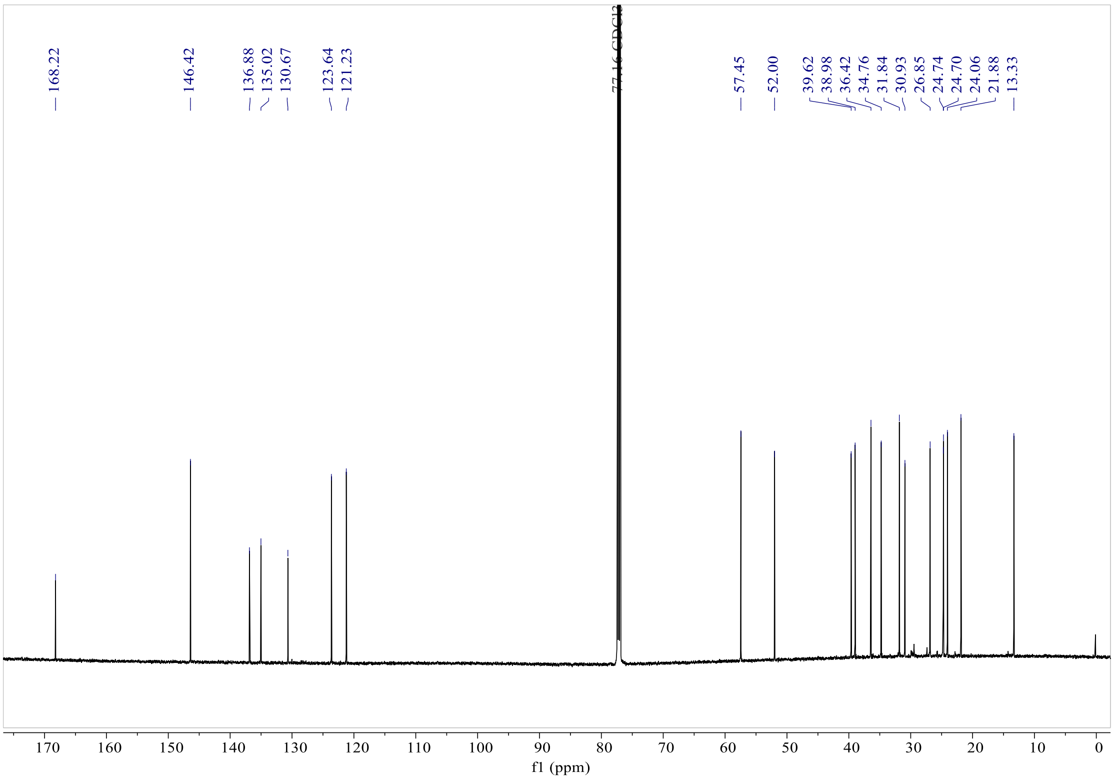

Figure S55. ^{13}C-NMR spectrum of biofloranate C (**7**) in $CDCl_3$

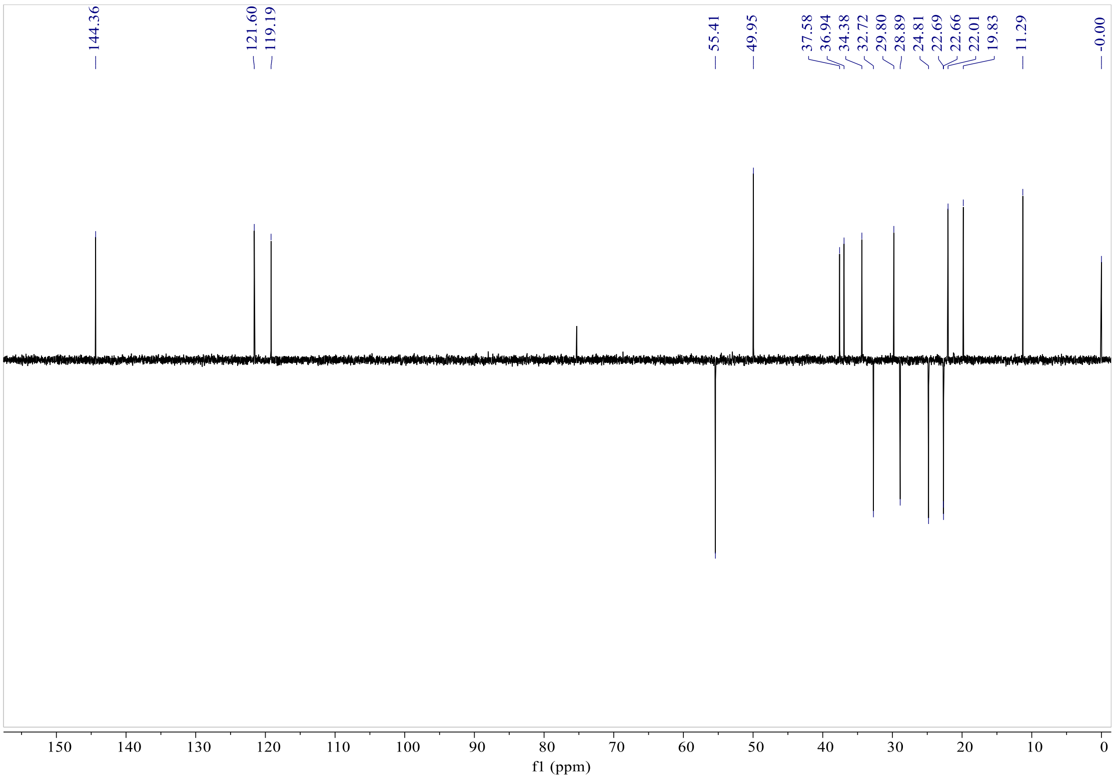

Figure S56. Dept 135° spectrum of biofloranate C (**7**) in $CDCl_3$

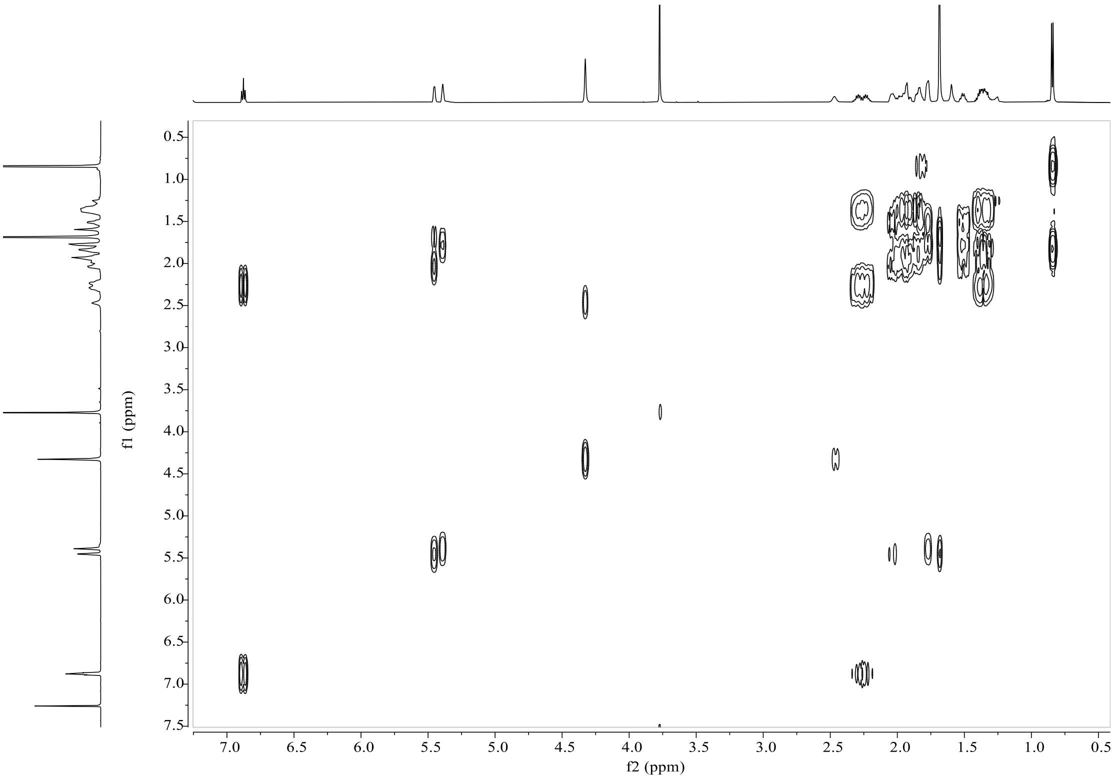

Figure S57. ^1H-^1H COSY spectrum of biofloranate C (**7**) in $CDCl_3$

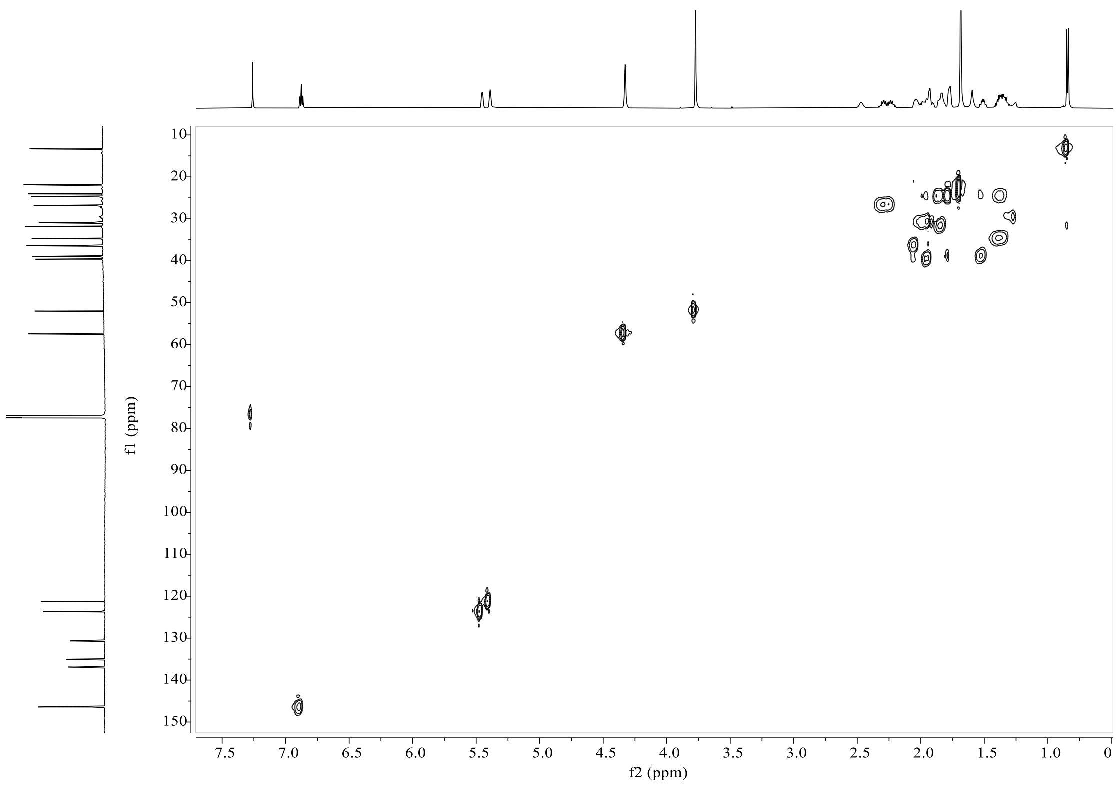

Figure S58. HSQC spectrum of biofloranate C (**7**) in $CDCl_3$

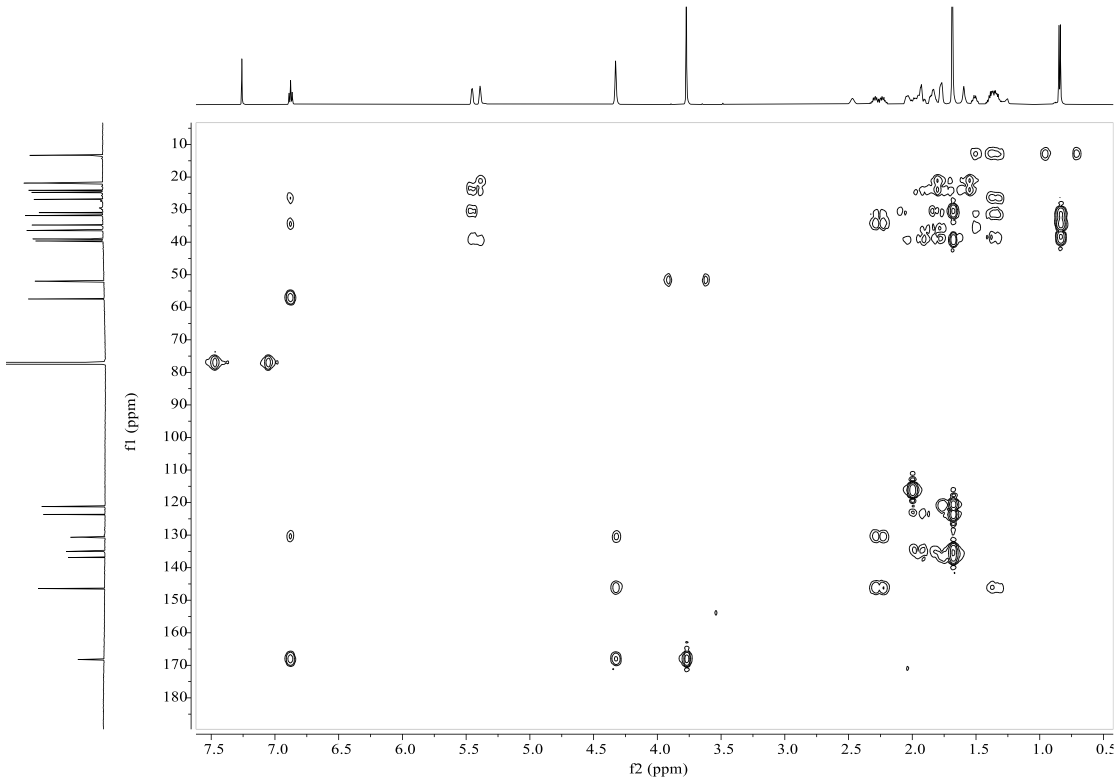

Figure S59. HMBC spectrum of biofloranate C (**7**) in $CDCl_3$

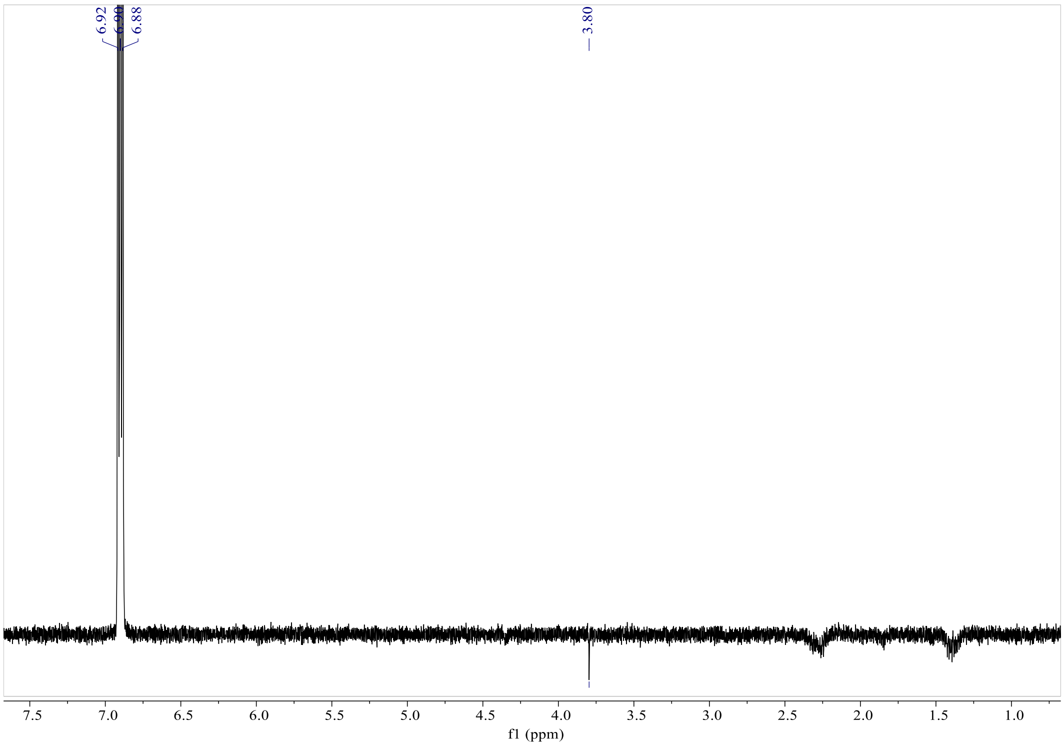

Figure S60. NOESY spectrum of biofloranate C (**7**) in $CDCl_3$

12. Spectroscopic data for biofloranate D (8)

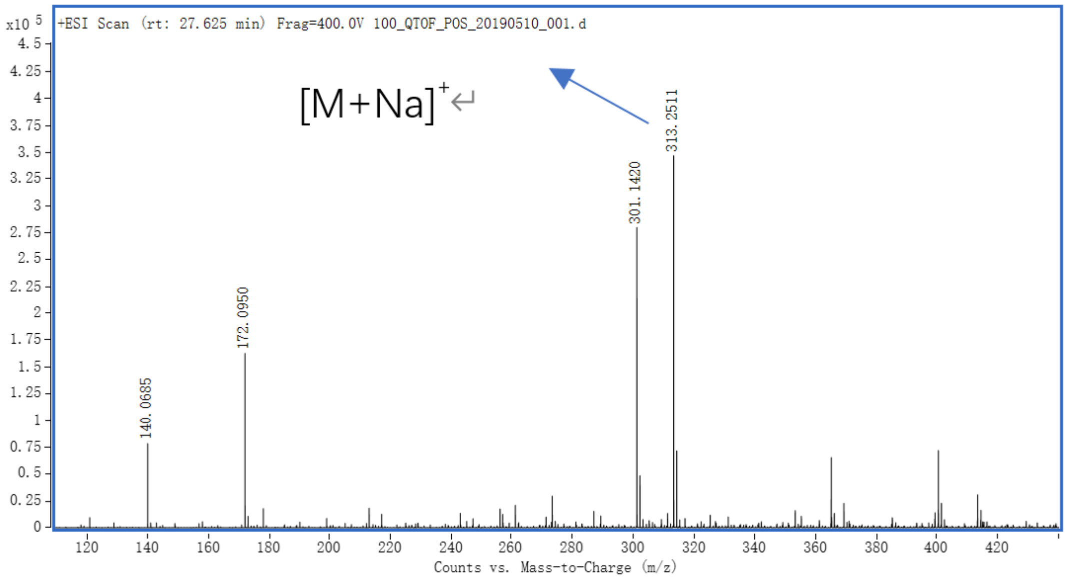

Figure S61. HRESIMS spectrum of biofloranate D (**8**)

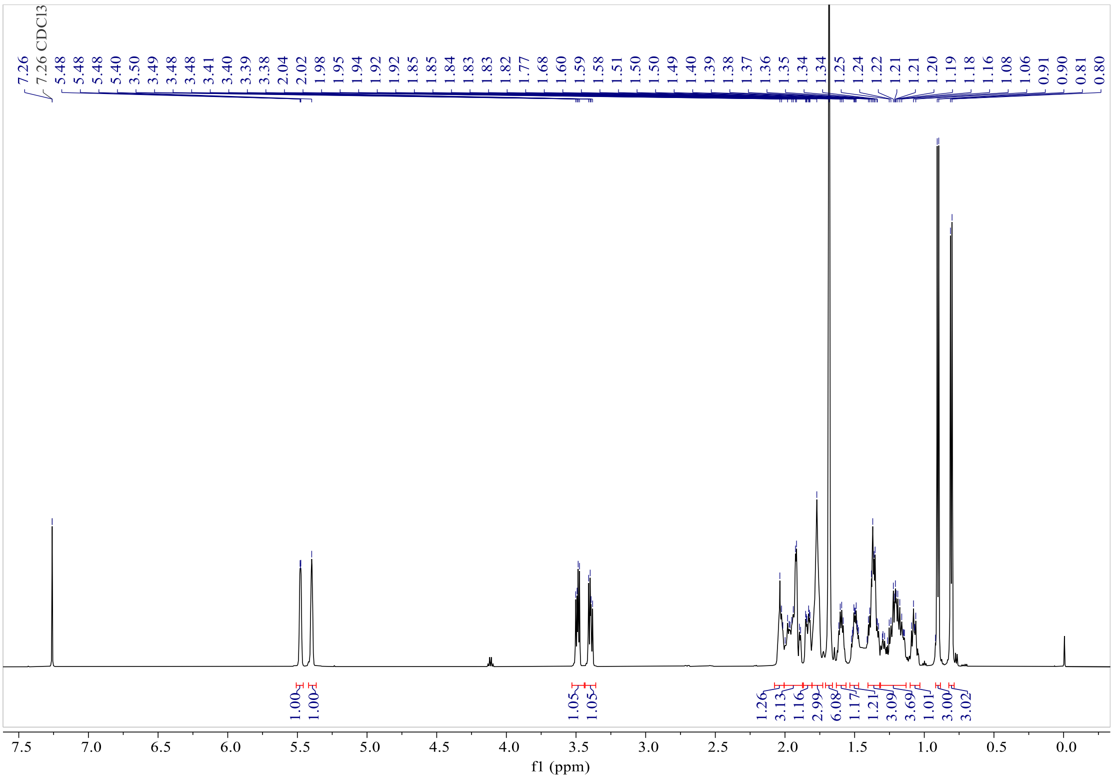

Figure S62. ^{1}H-NMR spectrum of biofloranate D (**8**) in $CDCl_3$

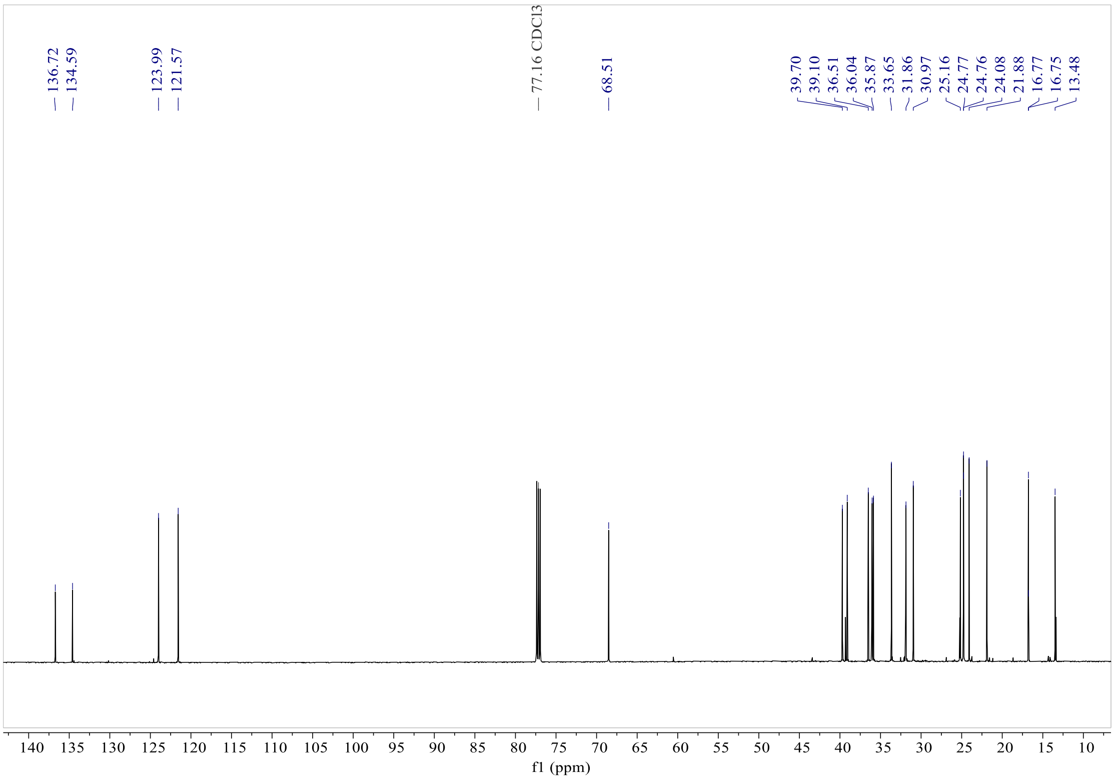

Figure S63. ^{13}C-NMR spectrum of biofloranate D (**8**) in $CDCl_3$

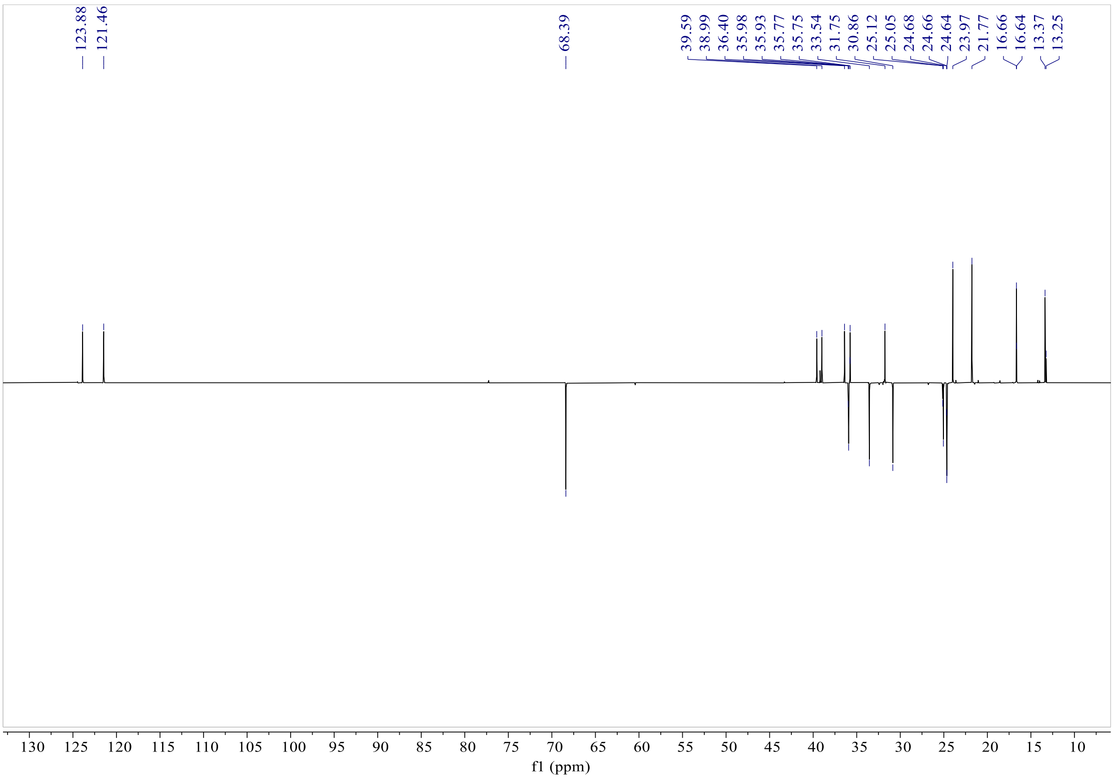

Figure S64. Dept 135° spectrum of biofloranate D (**8**) in $CDCl_3$

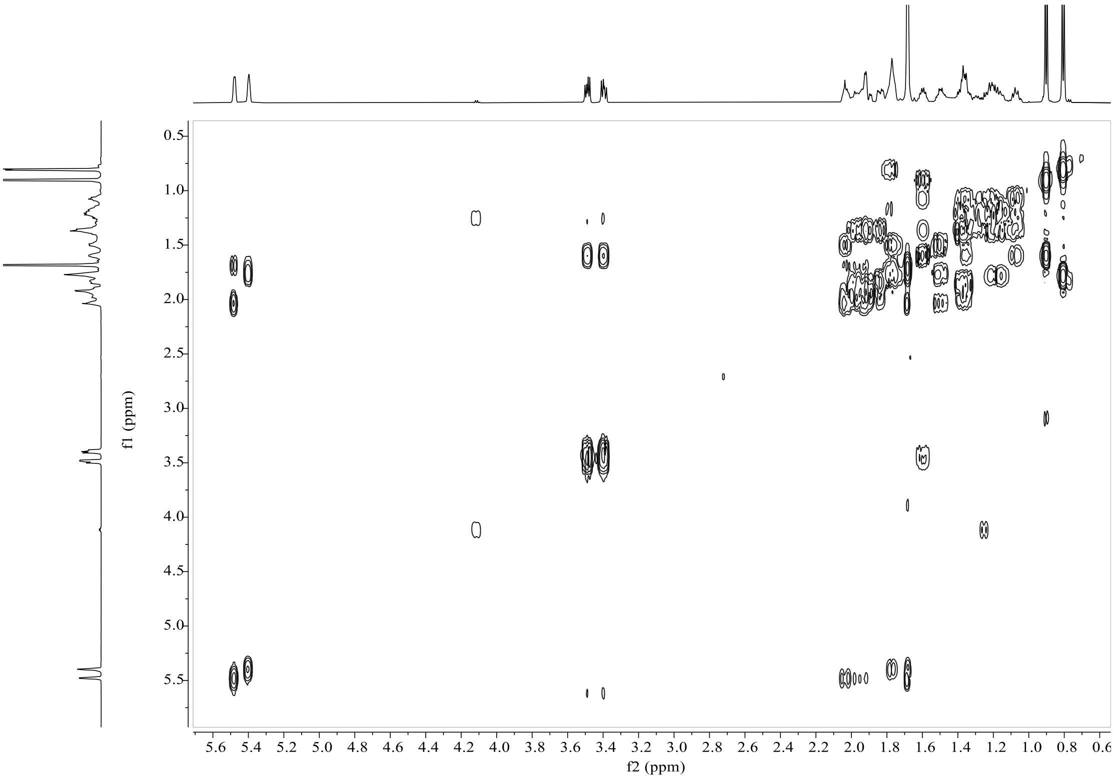

Figure S65. ^{1}H-^{1}H COSY spectrum of biofloranate D (**8**) in $CDCl_3$

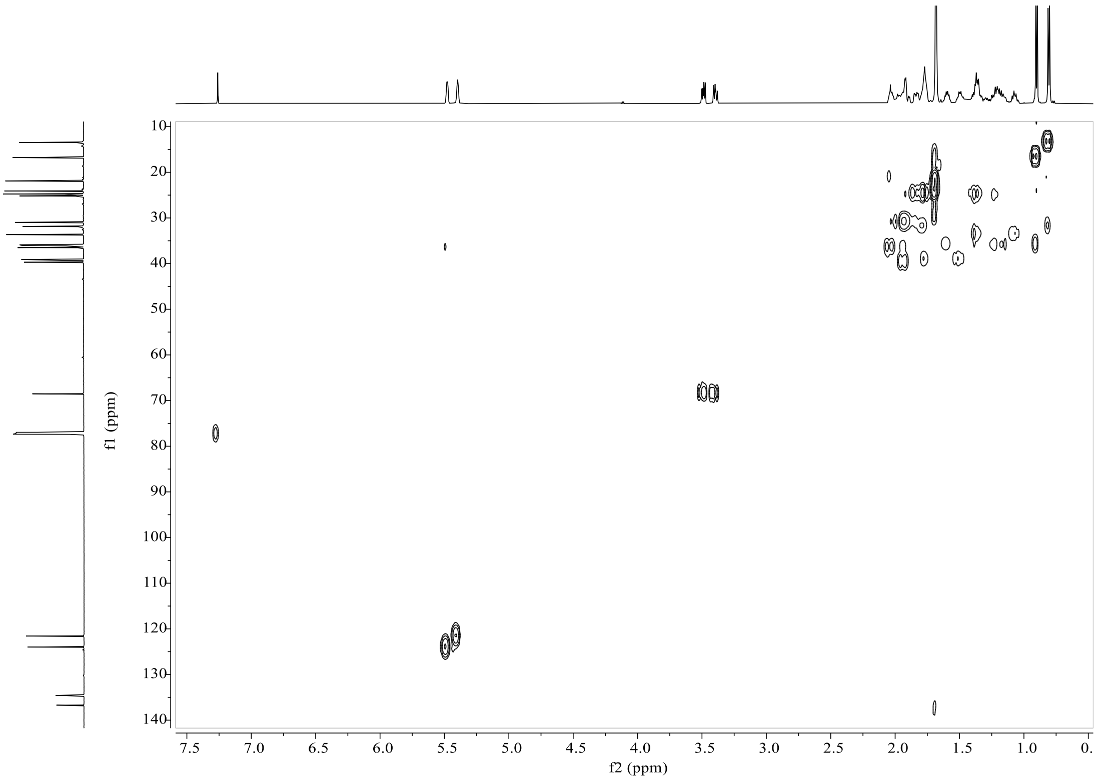

Figure S66. HSQC spectrum of biofloranate D (**8**) in $CDCl_3$

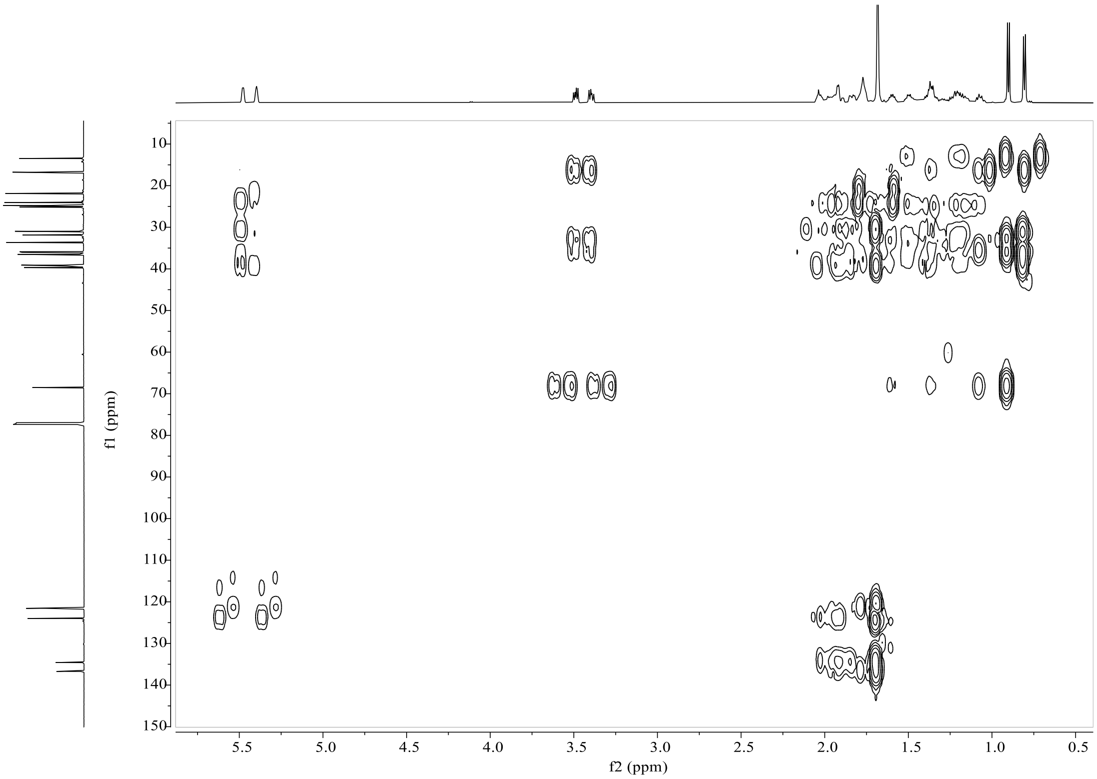

Figure S67. HMBC spectrum of biofloranate D (**8**) in $CDCl_3$

13. Spectroscopic data for euplexaurene D (9)

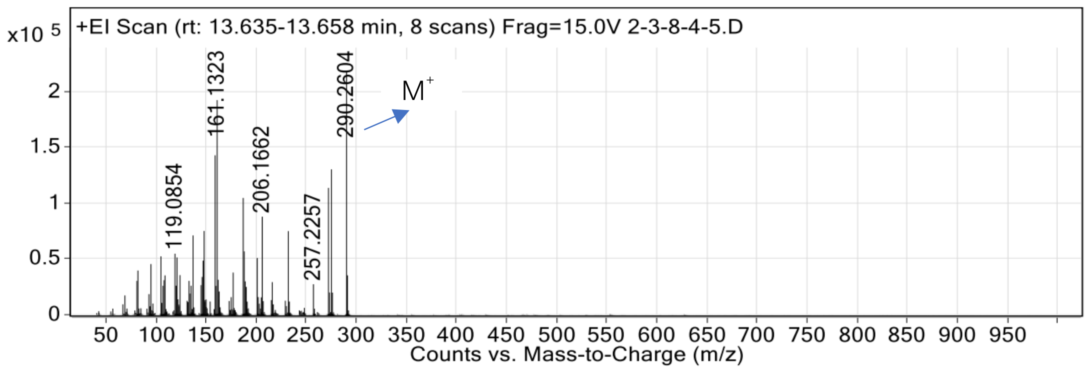

Figure S68. HRESIMS spectrum of euplexaurene D (**9**)

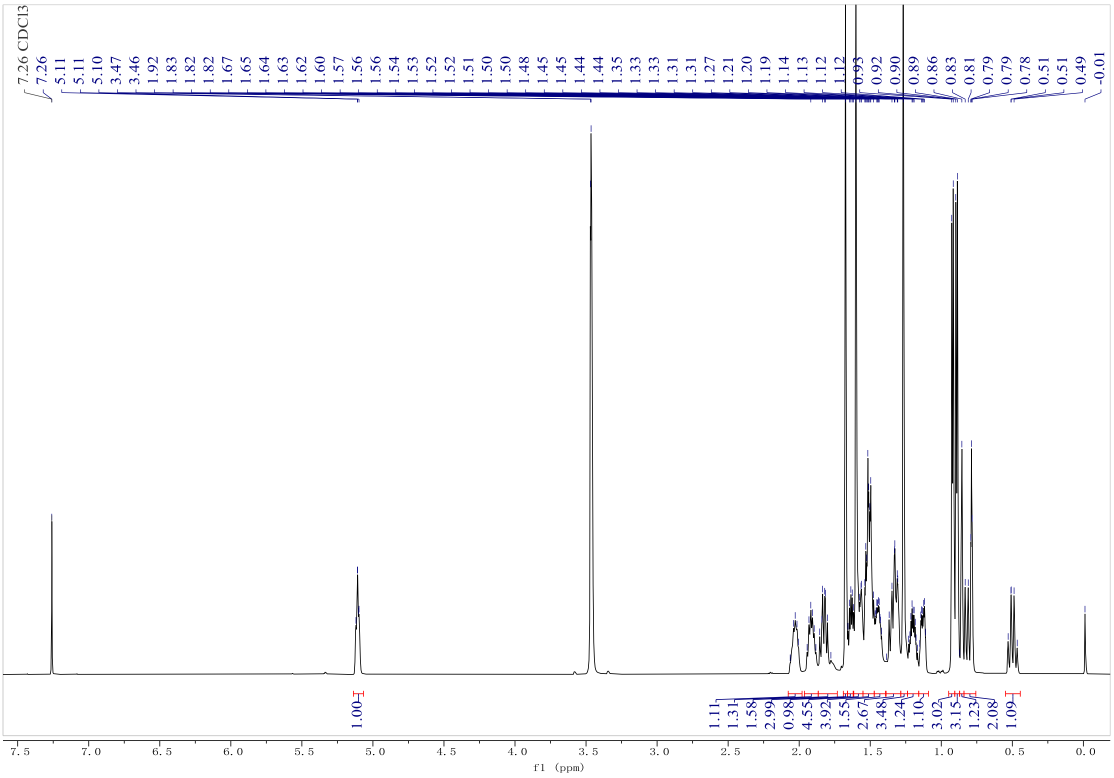

Figure S69. ^{1}H-NMR spectrum of euplexaurene D (**9**) in $CDCl_3$

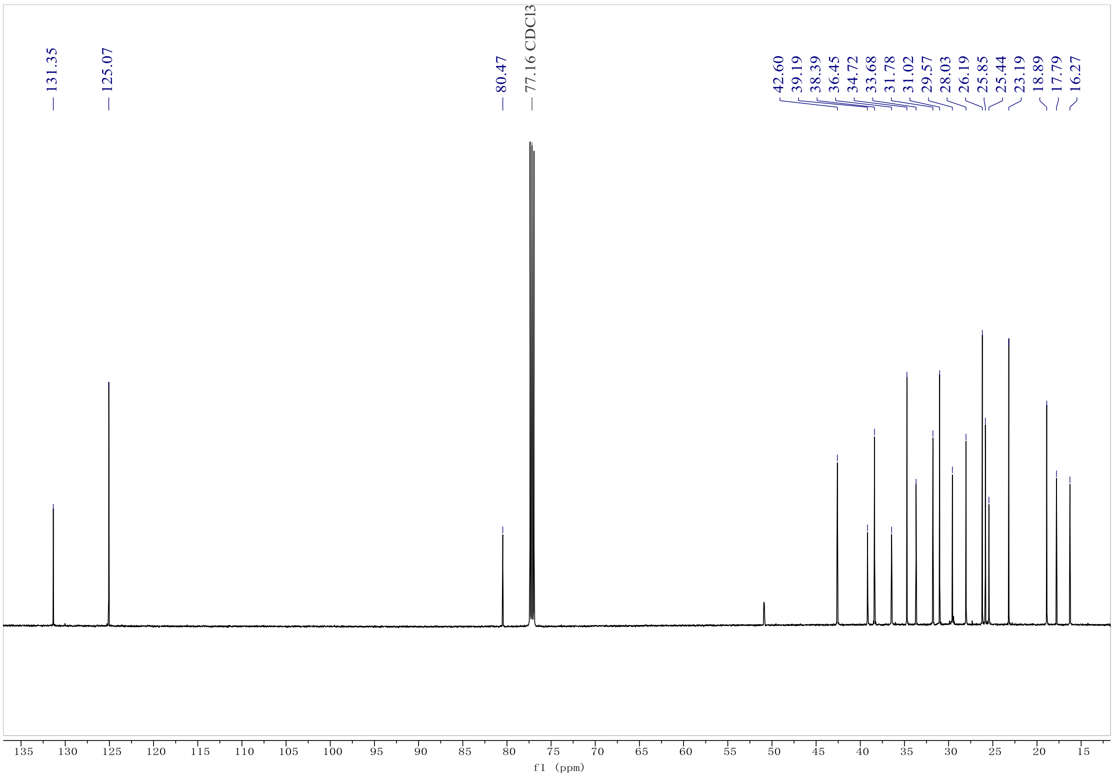

Figure S70. ^{13}C-NMR spectrum of euplexaurene D (**9**) in $CDCl_3$

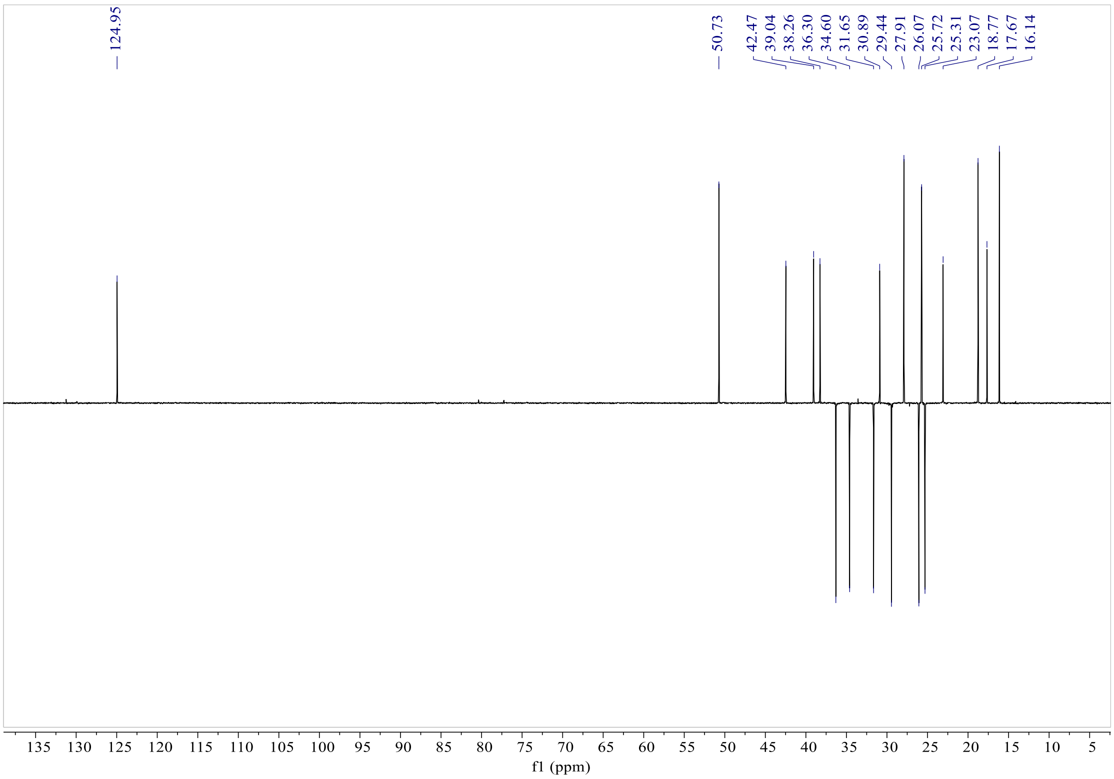

Figure S71. Dept 135° spectrum of euplexaurene D (**9**) in $CDCl_3$

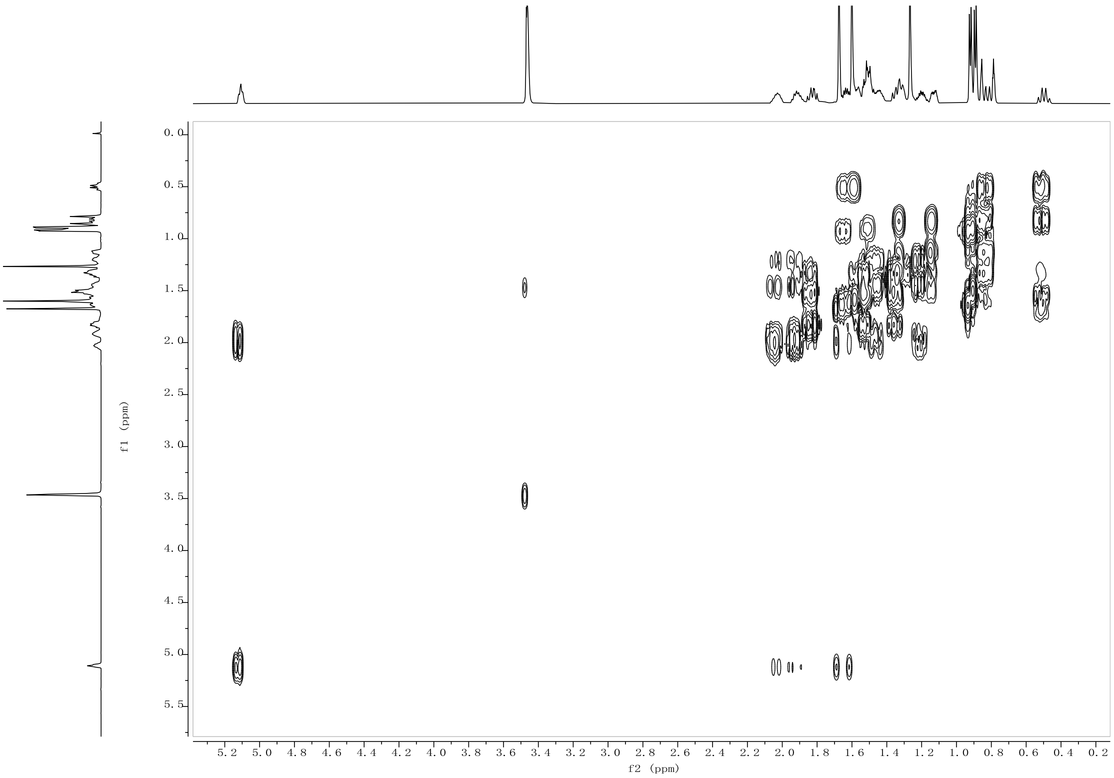

Figure S72. ^{1}H-^{1}H COSY spectrum of euplexaurene D (**9**) in $CDCl_3$

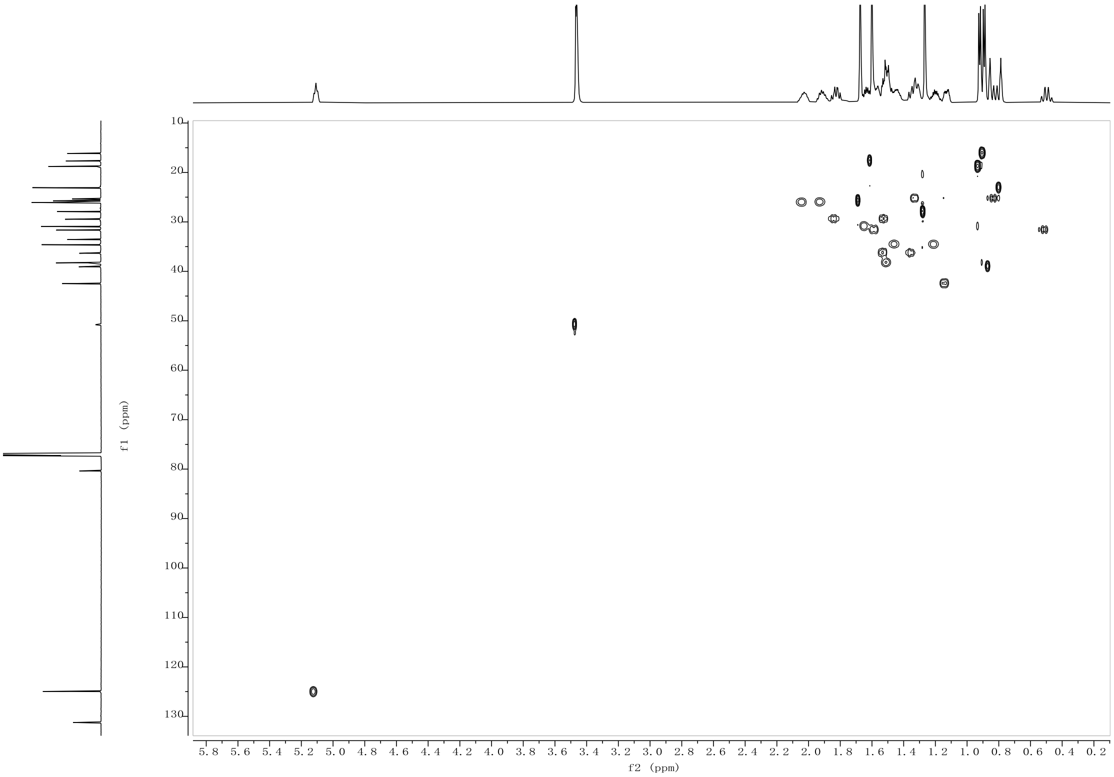

Figure S73. HSQC spectrum of euplexaurene D (**9**) in $CDCl_3$

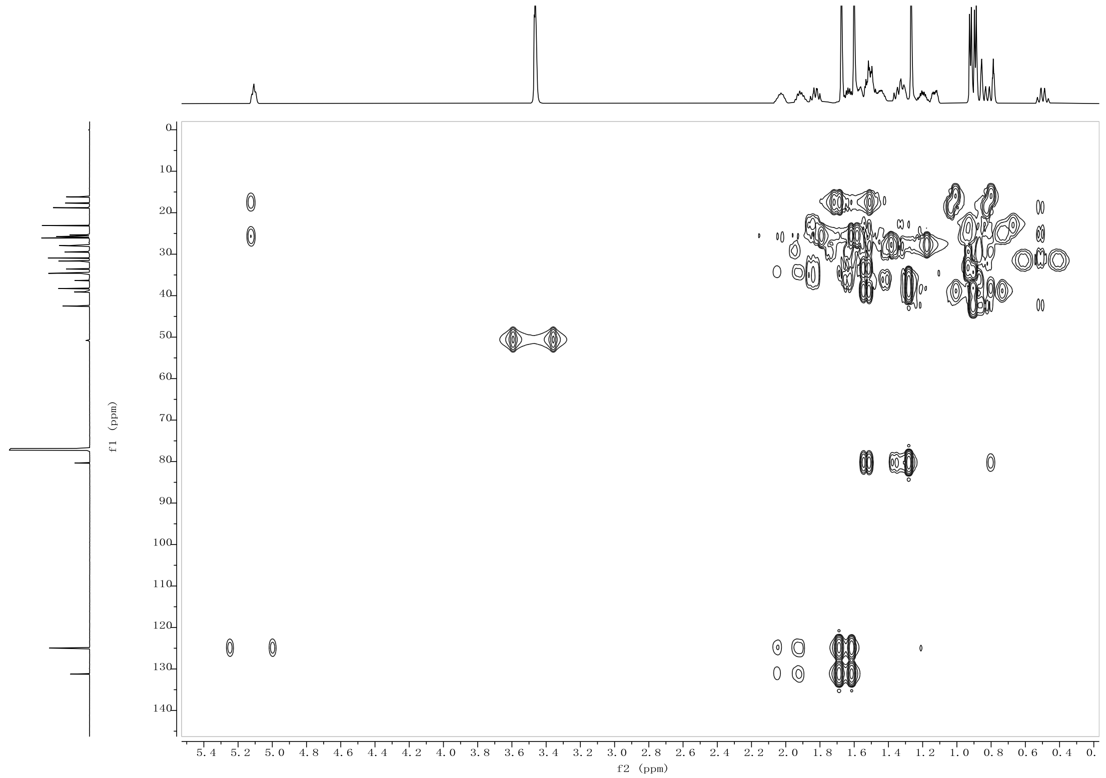

Figure S74. HMBC spectrum of euplexaurene D (**9**) in $CDCl_3$

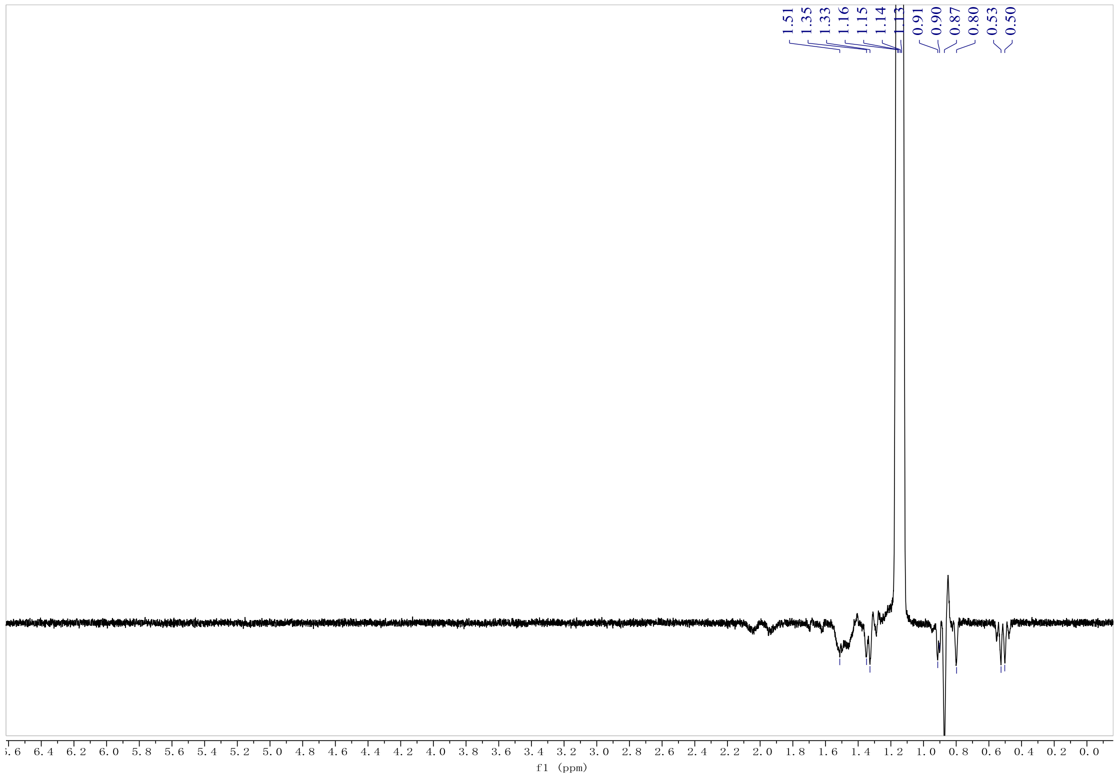

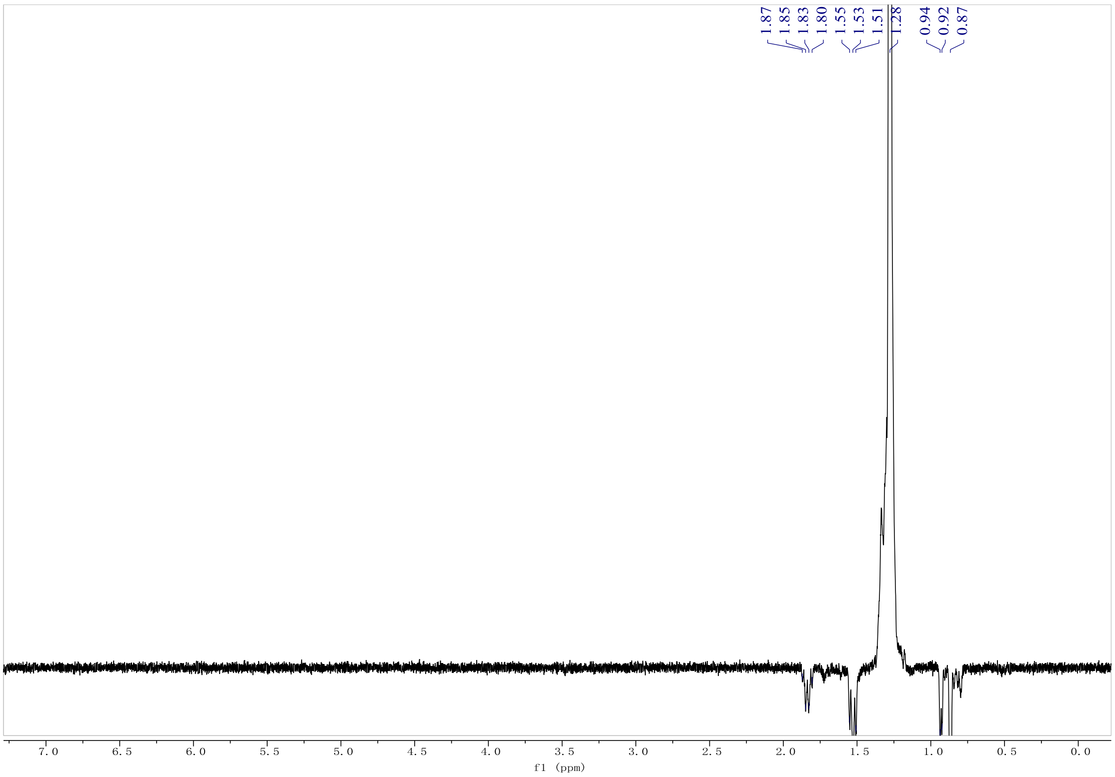

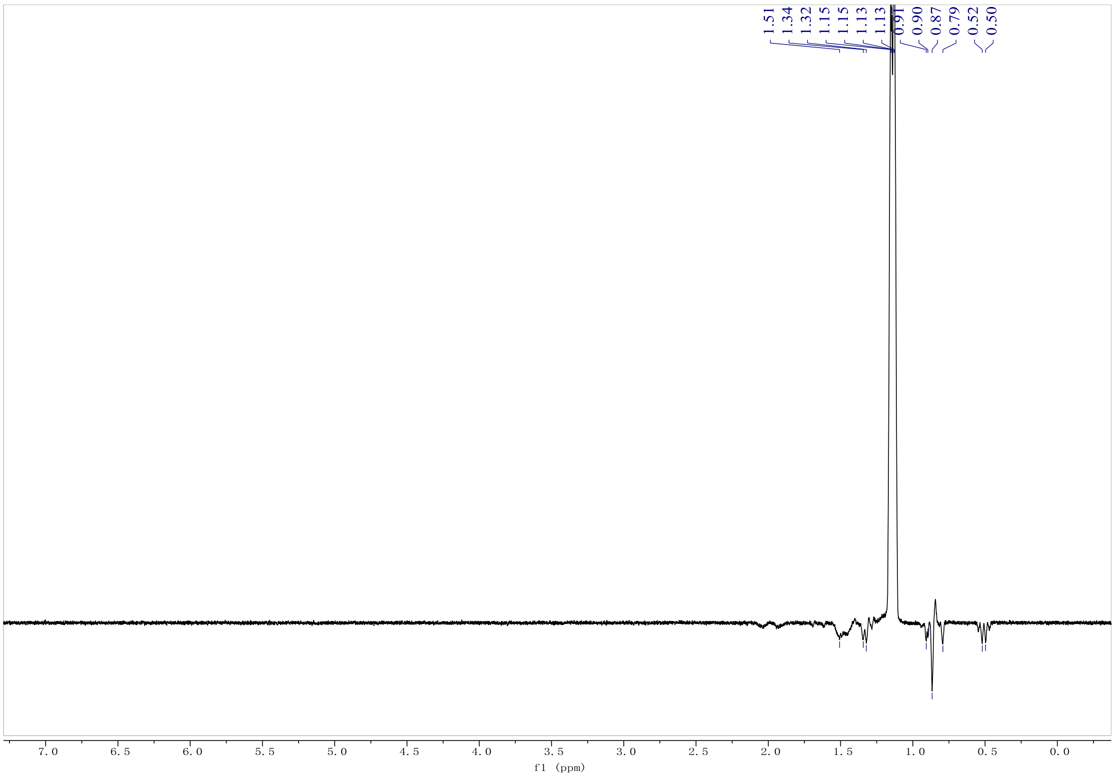

Figure S75. NOESY spectrum of euplexaurene D (**9**) in $CDCl_3$

14. Spectroscopic data for cneorubin K (10)

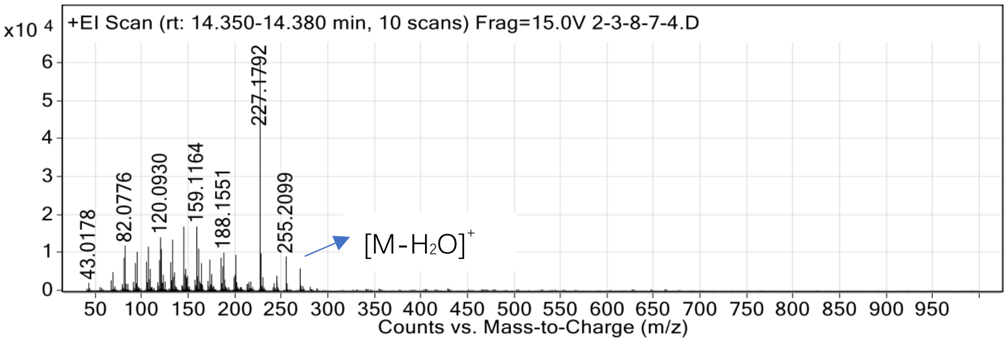

214.1701		1583.18
215.1783		2193.89
217.194		2309.28
219.1739		861.74
227.1792	1	59650.05
228.1823	1	9764.34
229.1869	1	1146.87
230.202	1	3462.92
231.2056	1	835.83
241.1946		797.61
242.2019		1874.33
245.189	1	3847.64
245.2253		2077.21
246.1928	1	802.48
255.2099	1	8896.82
256.2133	1	1813.48
270.2334	1	5782.26
271.2372	1	1305.07
273.2204		1087.19
281.0514		970.24

$[M-H_2O]^+$

Figure S76. HRESIMS spectrum of cneorubin K (**10**)

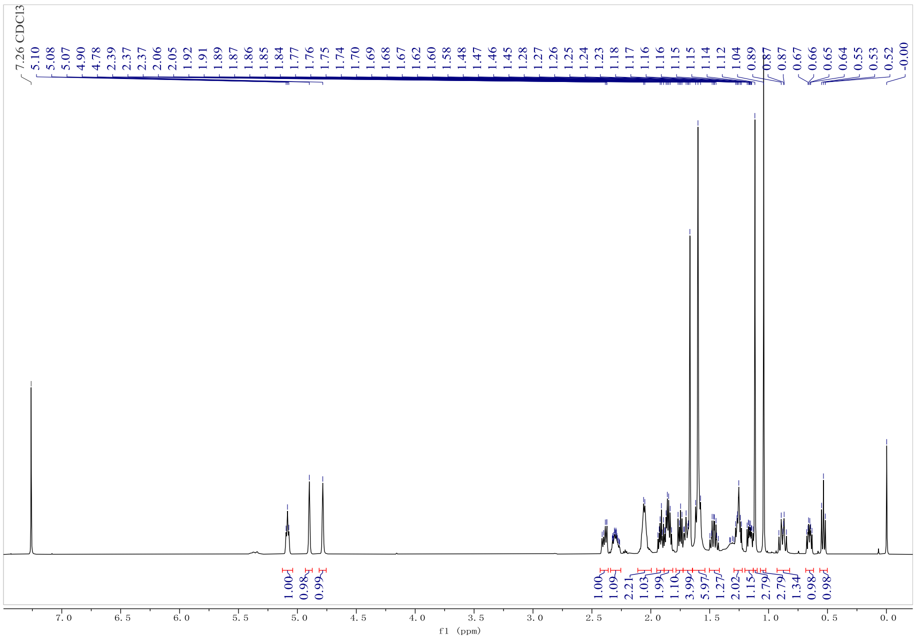

Figure S77. ^{1}H-NMR spectrum of cneorubin K (**10**) in $CDCl_3$

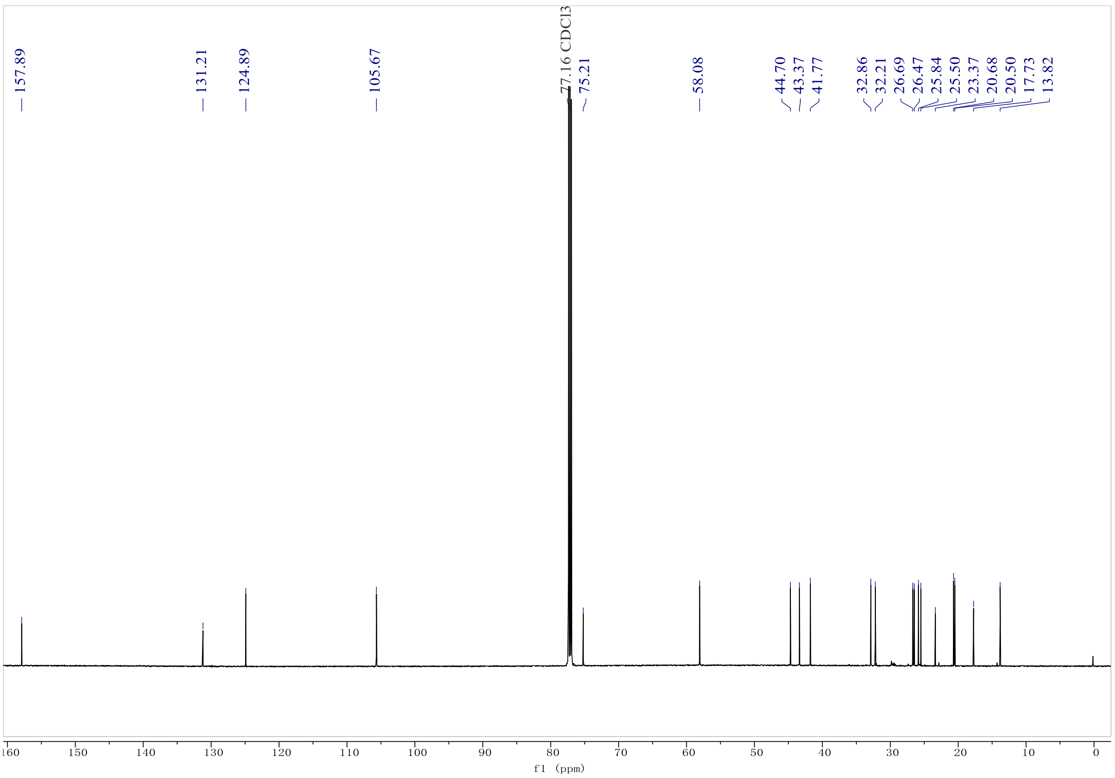

Figure S78. ^{13}C-NMR spectrum of cneorubin K (**10**) in $CDCl_3$

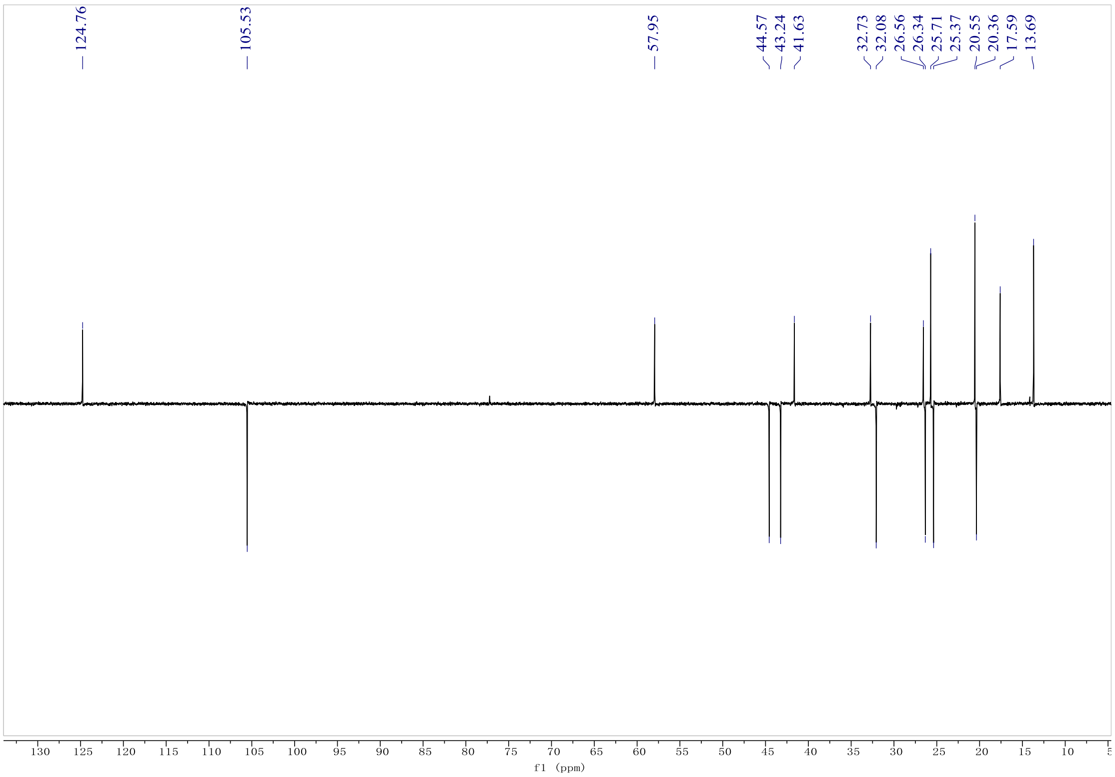

Figure S79. Dept 135° spectrum of cneorubin K (**10**) in $CDCl_3$

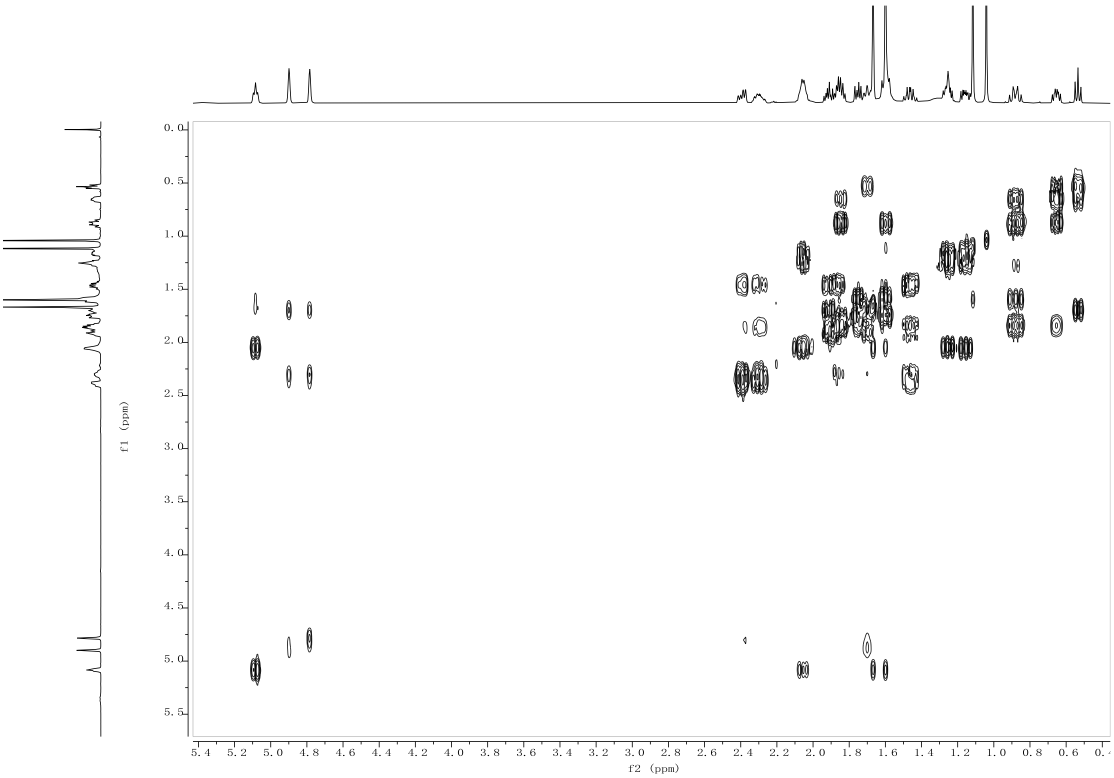

Figure S80. ^{1}H-^{1}H COSY spectrum of cneorubin K (**10**) in $CDCl_3$

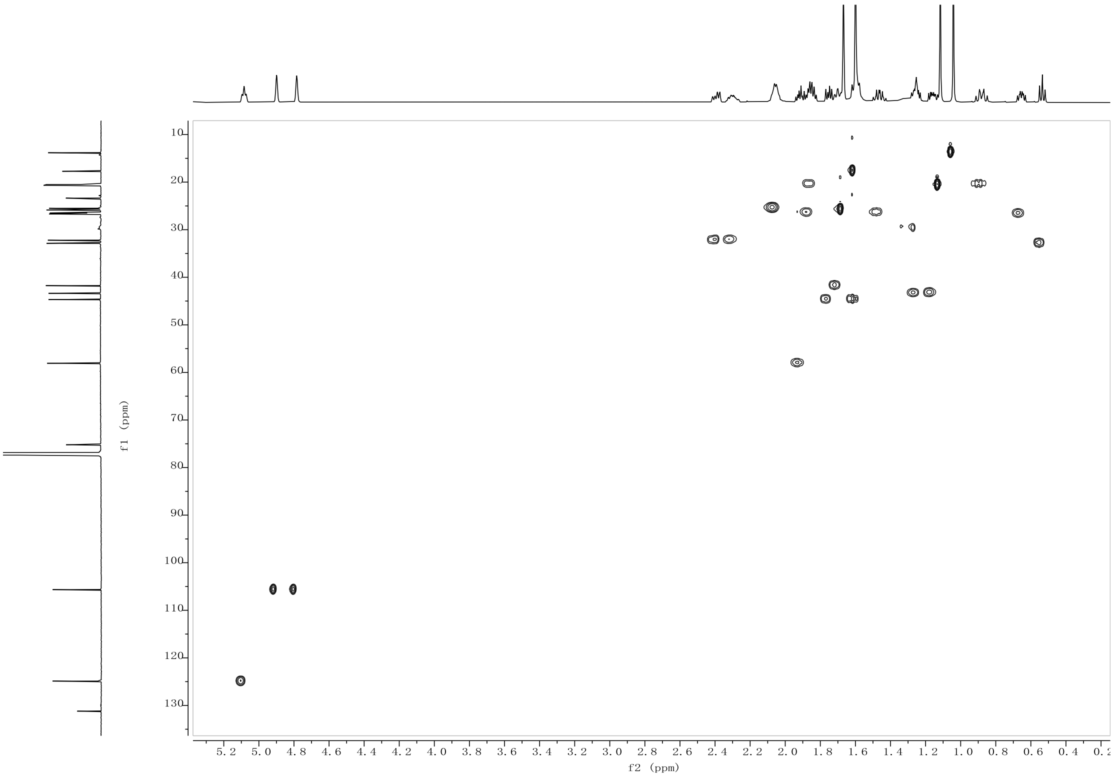

Figure S81. HSQC spectrum of cneorubin K (**10**) in $CDCl_3$

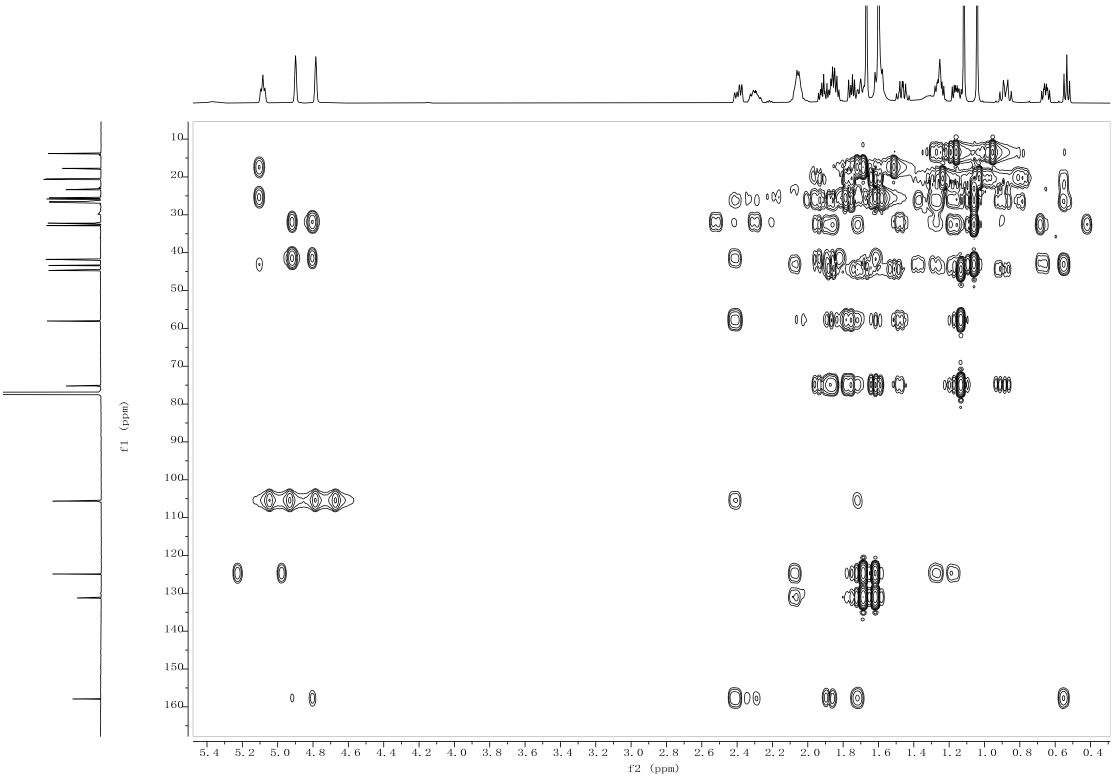

Figure S82. HMBC spectrum of cneorubin K (**10**) in $CDCl_3$

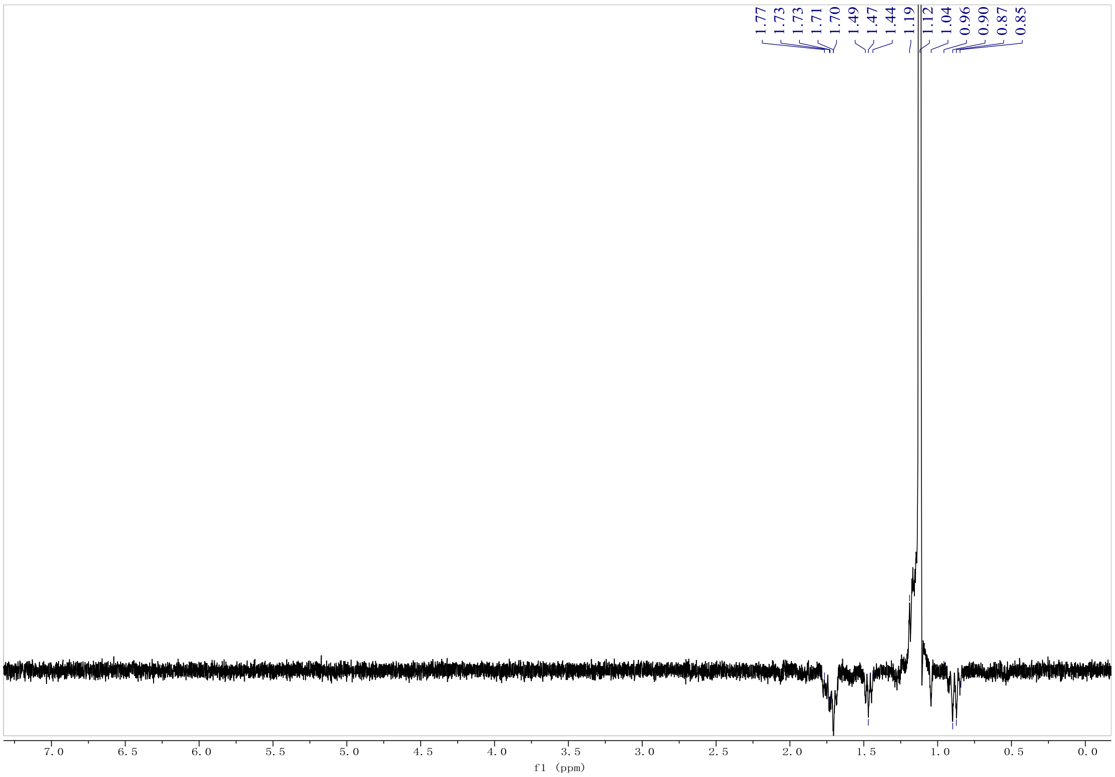

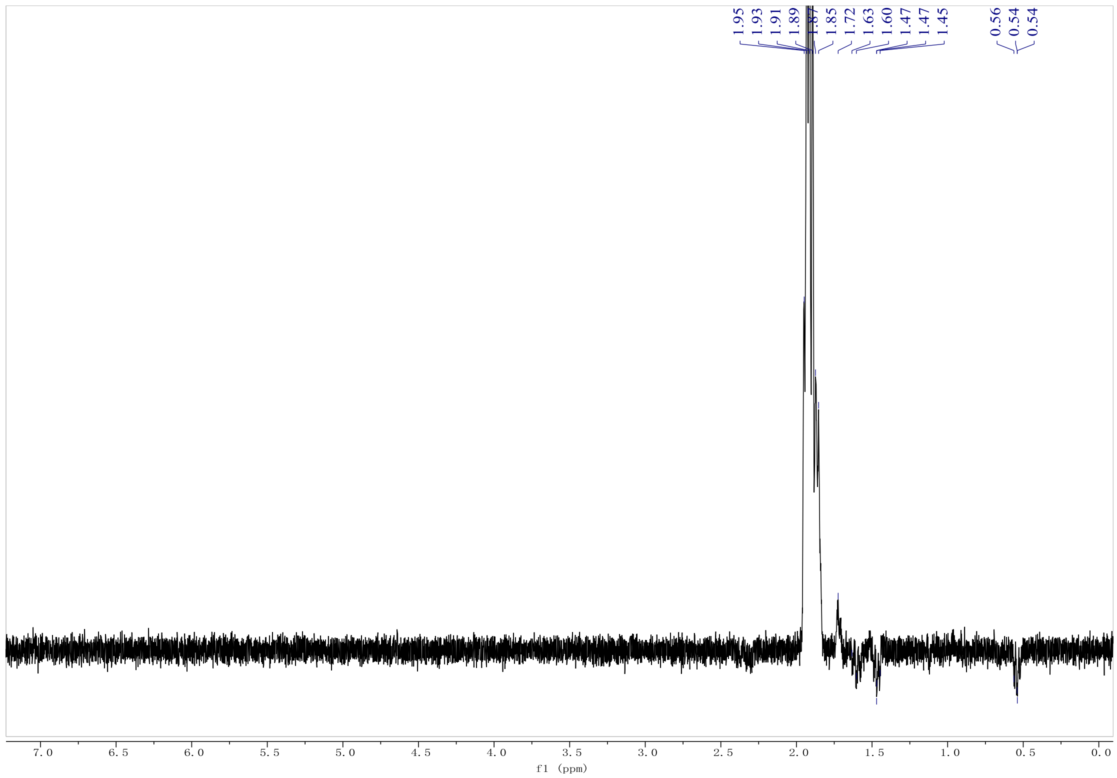
1.95
1.93
1.91
1.89
1.87
1.85
1.72
1.63
1.60
1.47
1.47
1.45
0.56
0.54
0.54
7.0
6.5
6.0
5.5
5.0
4.5
4.0
3.5
3.0
2.5
2.0
1.5
1.0
0.5
0.0
f1 (ppm)

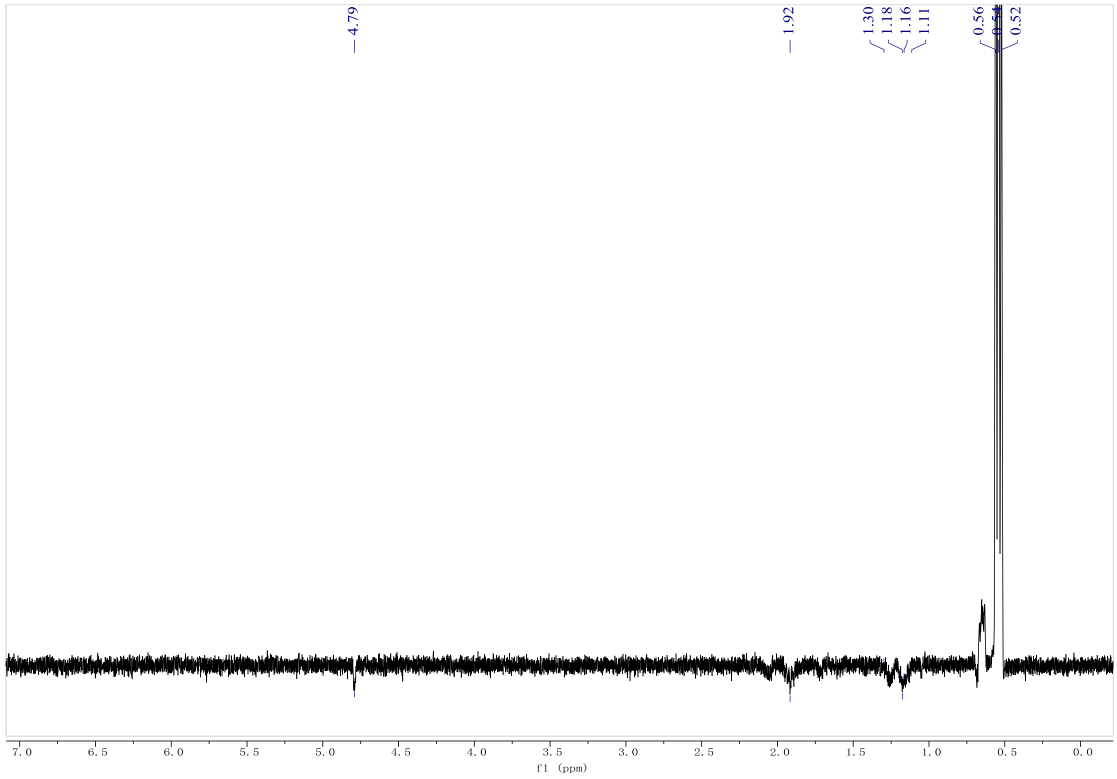
4.79
1.92
1.30
1.18
1.16
1.11
0.56
0.52
7.0
6.5
6.0
5.5
5.0
4.5
4.0
3.5
3.0
2.5
2.0
1.5
1.0
0.5
0.0
f1 (ppm)

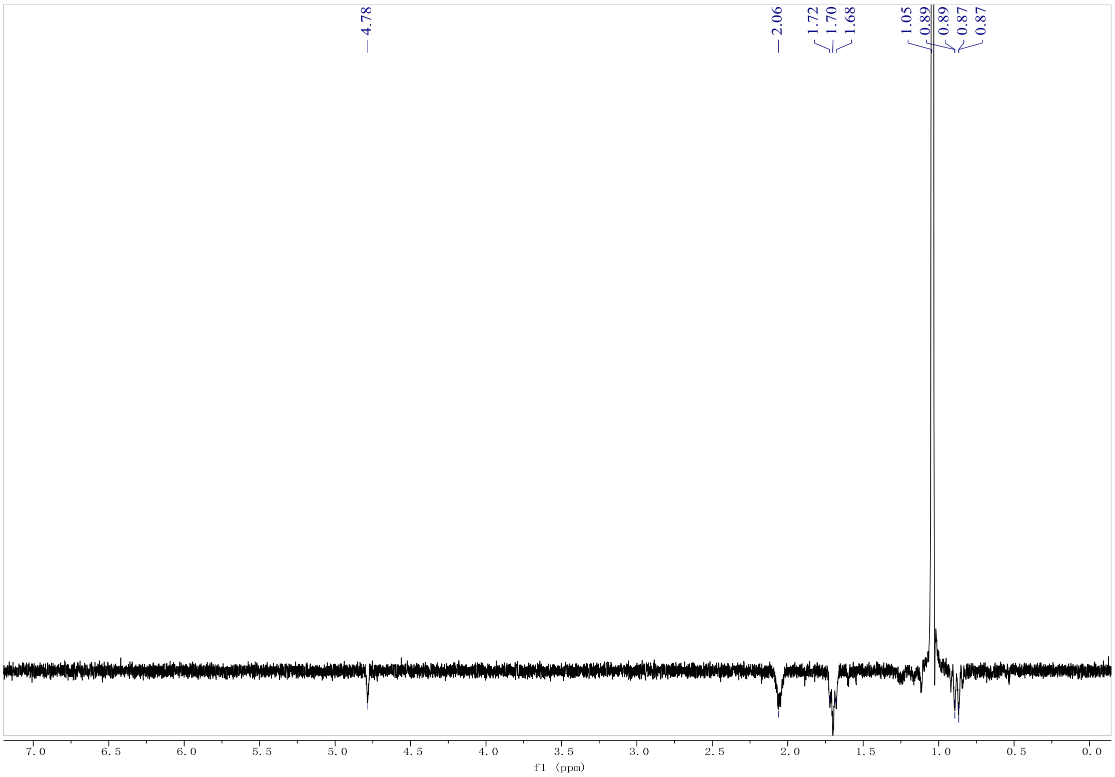

Figure S83. NOESY spectrum of cneorubin K (**10**) in $CDCl_3$

15. ^{1}H-NMR for (*S*)-MTPA and (*R*)-MTPA esters of compound 5 in pyridine-d_5

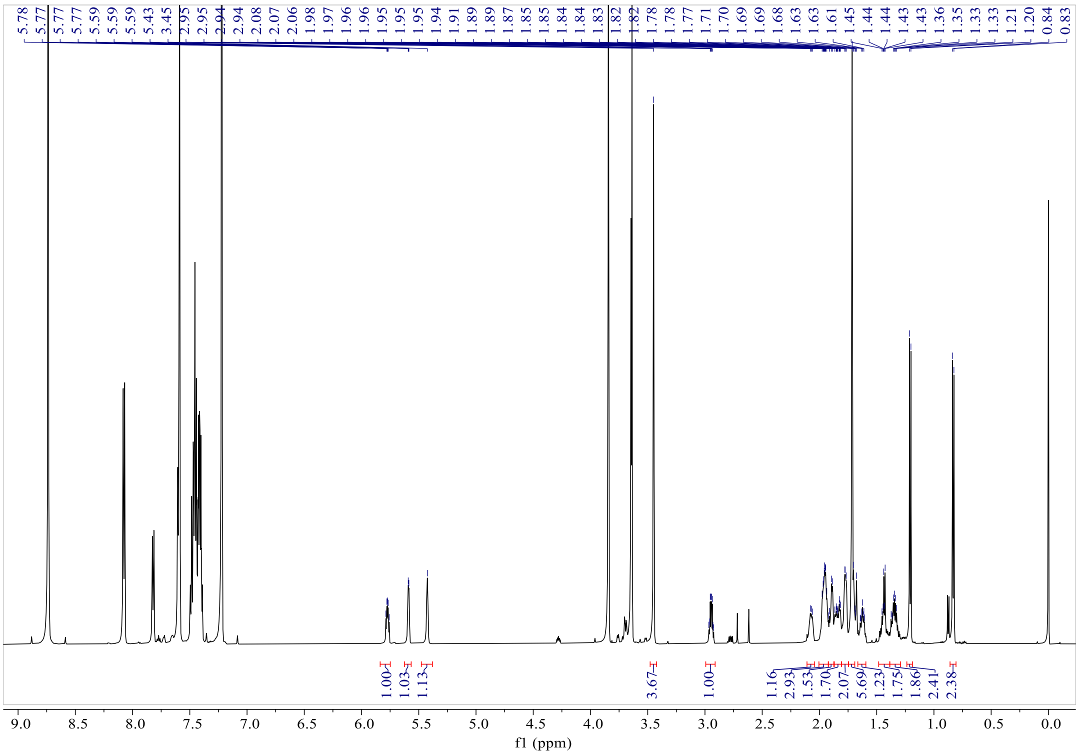

Figure S84. ^{1}H-NMR for (*S*)-MTPA ester of compound **5** in pyridine-d_5

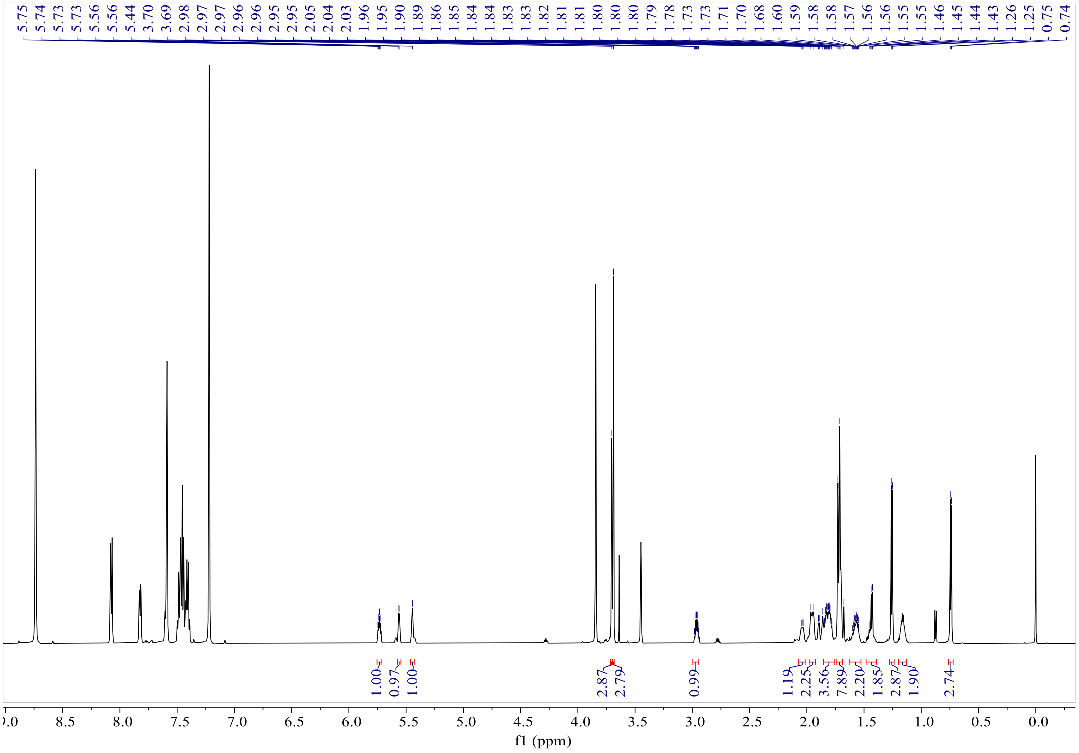

Figure S85. ^{1}H-NMR for (*R*)-MTPA ester of compound **5** in pyridine-d_5

16. Spectroscopic data for known compound cneorubin X (11)

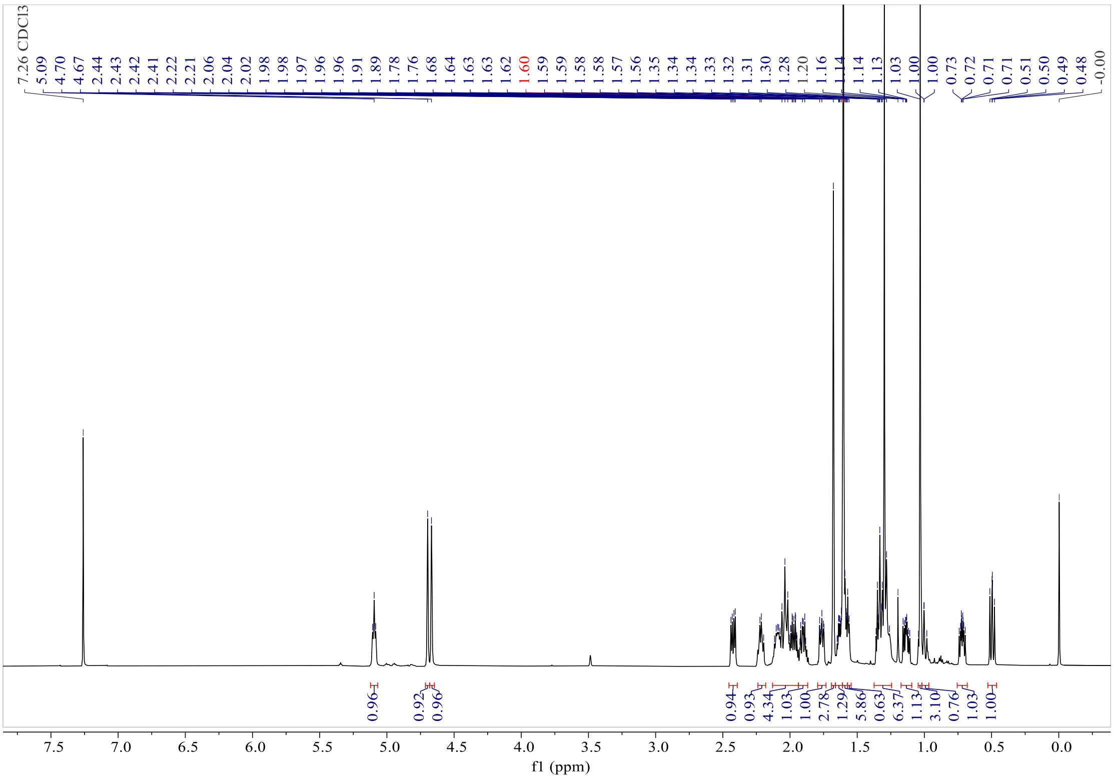

Figure S86. ^{1}H-NMR spectrum of cneorubin X (**11**) in $CDCl_3$

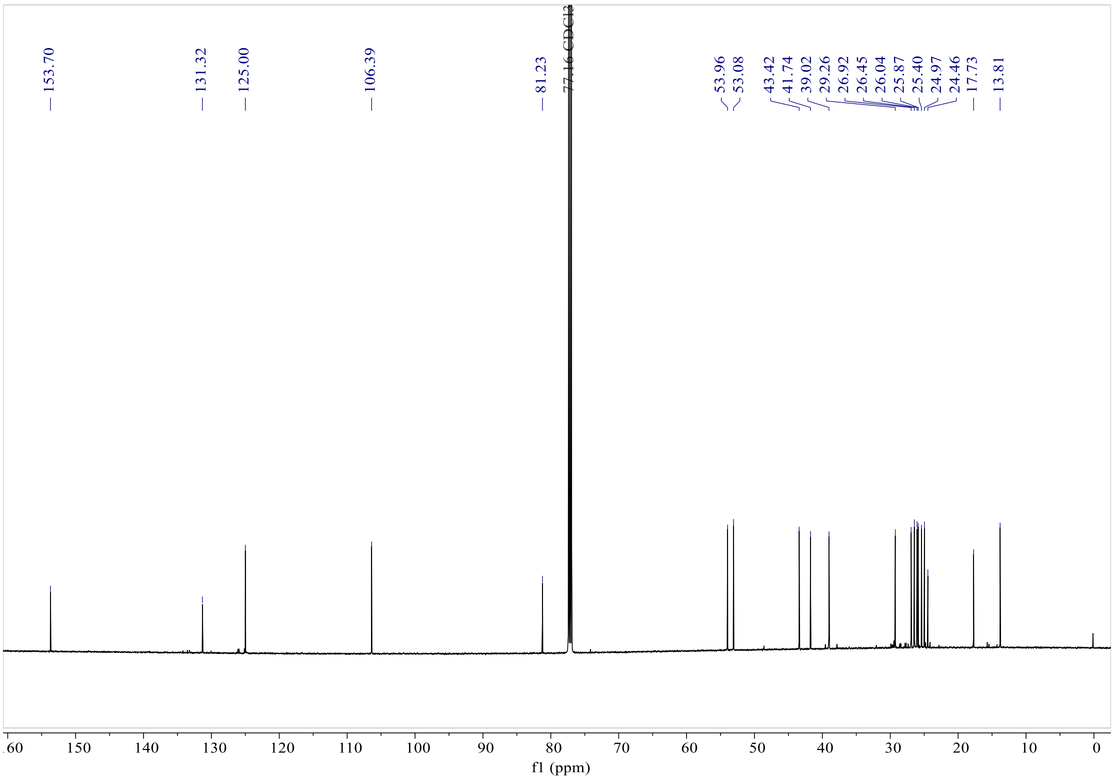

Figure S87. ^{13}C-NMR spectrum of cneorubin X (**11**) in $CDCl_3$

17. Spectroscopic data for known compound obscuronatin (12)

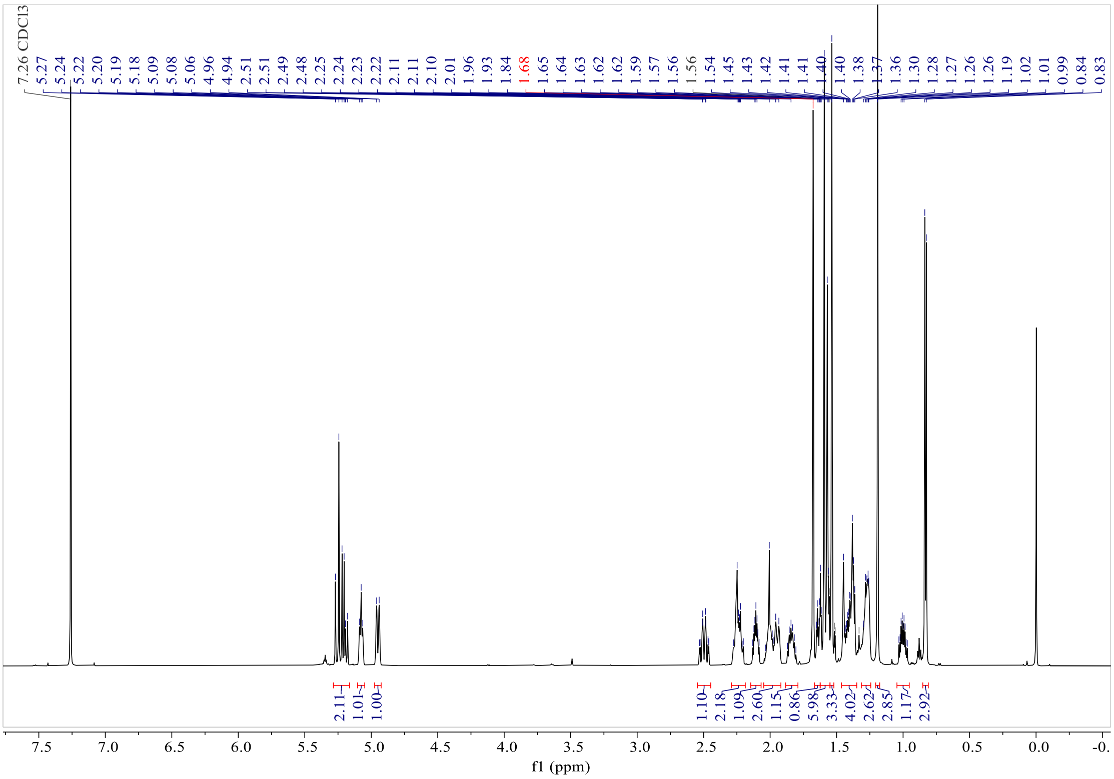

Figure S88. ^{1}H-NMR spectrum of obscuronatin (**12**) in $CDCl_3$

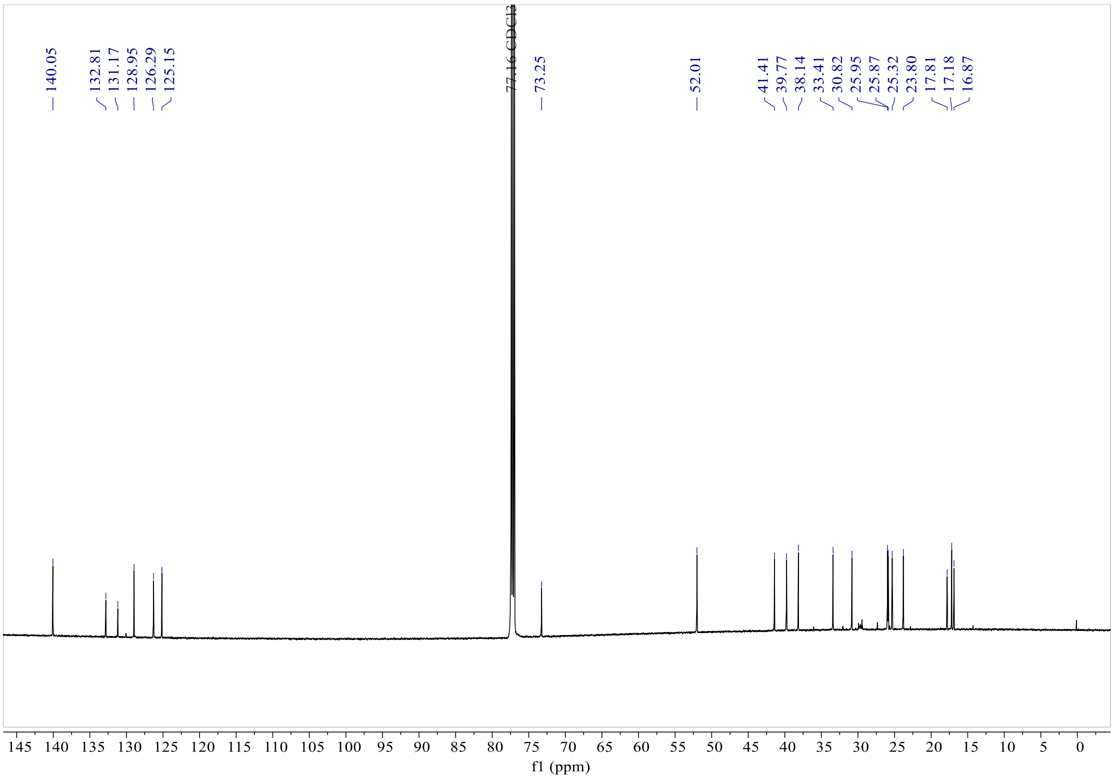

Figure S89. ^{13}C-NMR spectrum of obscuronatin (**12**) in $CDCl_3$

18. Spectroscopic data for known compound dictyotin B (13)

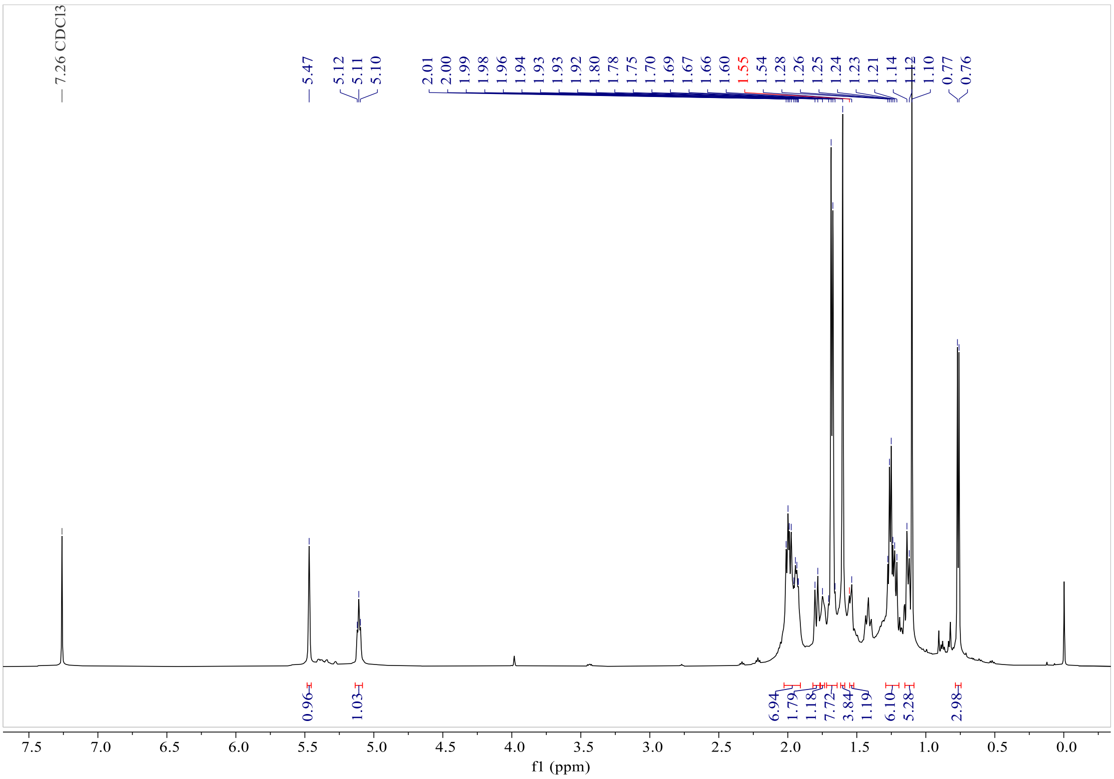

Figure S90. ^{1}H-NMR spectrum of dictyotin B (**13**) in $CDCl_3$

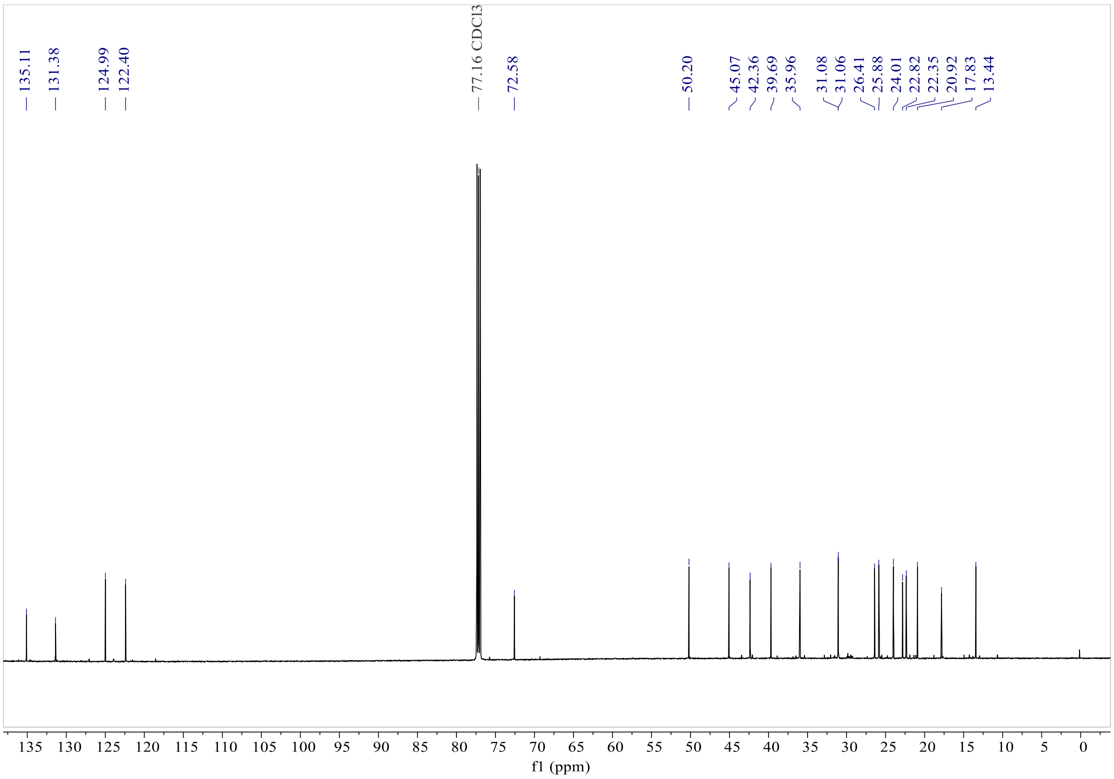

Figure S91. ^{13}C-NMR spectrum of dictyotin B (**13**) in $CDCl_3$
